# Design, Synthesis, and In Vitro Evaluation of the Leishmanicidal Activity of New Aromatic Symmetrical 1,4-Disubstituted 1,2,3-Bistriazoles

**DOI:** 10.1021/acsomega.5c00492

**Published:** 2025-05-12

**Authors:** Maurício Moraes Victor, Gabriel dos Santos Ramos, Bruno Silva Andrade, Patrícia Ferreira Espuri Sepin, Guilherme Álvaro Ferreira-Silva, Marisa Ionta, Amanda Almeida Morais, Vanessa Silva Gontijo, Claudio Viegas, Marcos José Marques

**Affiliations:** a Department of Organic Chemistry, Chemistry Institute, Federal University of Bahia, Salvador, BA 40170-115, Brazil; b National Institute of Science and Technology for Energy and Environmental, Salvador, BA 40170-115, Brazil; c Department of Biological Sciences, State University of Southwest of Bahia, Jequié, BA 45208-091, Brazil; d Institute of Biomedical Sciences, 74347 Federal University of Alfenas, Alfenas, MG 37130-001, Brazil; e PeQuim − Laboratory of Research in Medicinal Chemistry, Institute of Chemistry, 74347 Federal University of Alfenas, Alfenas, MG 37133-840, Brazil

## Abstract

A second generation of symmetrical 1,4-disubstituted 1,2,3-triazoles containing aromatic moieties was designed and synthesized from 2-hydroxy-1,3-bisazido-propane and terminal alkynes by copper­(I)-catalyzed alkyne–azide cycloaddition (CuAAC) as potential inhibitors of protein CYP51. The symmetrical bistriazoles (SBs) were obtained in moderate to excellent yields (49 to 95%). All synthetic nonsymmetric triazoles and the symmetric bistriazole derivatives were in vitro screened for the extracellular promastigote forms of *Leishmania amazonensis*. From this investigation, it emerged that symmetric bistriazole 12c (IC_50_ = 19.24 μM) showed the highest potency against the flagellate form of the parasite followed by compounds 12b (IC_50_ = 34.46 μM), 10 (IC_50_ = 44.13 μM), and 4 (IC_50_ = 42.81 μM). The cytotoxicity evaluation revealed that the most active compounds were also significantly toxic with SI ∼2, except for compounds 4 and 10 that showed SI values of 7.51 and 8.69, respectively. Considering SI > 8 as a selection criterion, only the diketone-bistriazole derivative 10 was submitted to further evaluation against the intracellular amastigote form, showing a significant cytotoxic effect, with an IC_50_ value of 68.38 μM (SI = 5.61). To evaluate its potential toxicity on normal human cells, the most promising compound 10 was also assayed against human fibroblasts culture, showing a significantly smaller cytotoxicity (CC_50_ = 568.09 μM, SI = 8.3) in comparison to amphotericin B (CC_50_ = 22.95 μM, SI = 12.07). An in silico investigation showed that the most promising compound 10 bound inside the active pocket from the protein CYP51 with a binding energy of −9.96 kcal/mol.

## Graphical abstract

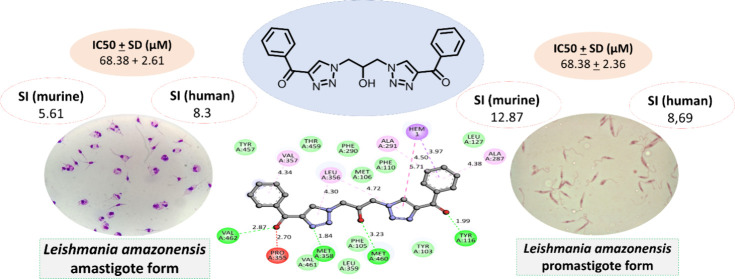

## 1 Introduction

Leishmaniasis is a group of neglected tropical diseases caused by an obligate intracellular protozoan of the genus *Leishmania*.^1,2^ The disease affects the poorest world populations throughout Africa, Asia, Central and South America, and the Middle East. The protozoan infection is mainly transmitted to humans by the female phlebotomine sandflies in the Old World and by *Lutzomyia* spp. in the New World. Approximately 350 million people are estimated to be continuously at infection risk, and about 12–15 million people worldwide are infected.^3^ The incidence of cutaneous leishmaniasis (CL) ranges from 700,000 to 1.2 million new cases per year, while estimates of the annual occurrence of visceral leishmaniasis (VL), also known as kala-azar, are currently less than 100,000. However, despite being a less common disease type, VL accounts for the most severe manifestations and is responsible for 70,000 annual deaths.^4^ Approximately 95% of CL cases occur in the Americas, Mediterranean basin, Middle East, and Central Asia, while VL has the highest incidence in Brazil, East Africa, and India. As a third form of the disease, mucocutaneous leishmaniasis (MCL) has over 90% of prevalence in Bolivia, Brazil, Ethiopia, and Peru and is characterized by affecting the mouth, nose, and throat of patients.^5^ The genus *Leishmania* belongs to the family Trypanosomatidae and covers more than 20 human-infecting species. To date, it is well-known that several types and clinical manifestations of leishmaniasis are related to infections caused by different parasite species, which also vary from one endemic region to another. In this regard, CL is a result of the infection with *L. tropica*, *L. major*, *L. aethiopica*, *L. infantum* (also known as *L. chagasi*), *L. amazonensis*, *L. braziliensis*, *L. mexicana*, *L. peruviana*, *L. guayanensis*, and *L. panamesis*, while VL is mainly caused by *L. donovani* and *L. infantum*. On the other hand, *L. brazilensis*, *L. panamensis*, *L. guyanensis*, *L. infantum*, and *L. donovani* are the parasite species known as responsible for the occurrence of MCL.^1^

To date, the therapeutical arsenal available for the clinics of leishmaniasis is restricted to a few drugs, frequently associated with significant adverse effects and toxicity, chemical resistance, lack of specificity, and prolonged treatment regimen, which contribute to difficulties related to the adhesion of patients to the therapeutical approach.^6^ The main available drugs on the world market are pentamidine, sodium stibogluconate, miltefosine, amphotericin B, and ketoconazole, whose chemical structures are depicted in Figure 1. For examples of side effects, the pentavalent antimony salt sodium stibogluconate causes cardiotoxicity, drug resistance, and pancreatitis, while the adverse effects associated with pentamidine include myalgia, breathing difficulty, and drug resistance. In the same direction, amphotericin B has as its main complications nephrotoxicity, hypotension, and hypokalemia.^7^ To overcome these toxicity issues, research focused on the design and development of newer safe and effective drugs against Leishmania is a challenge.^5–8^

**Figure 1:**
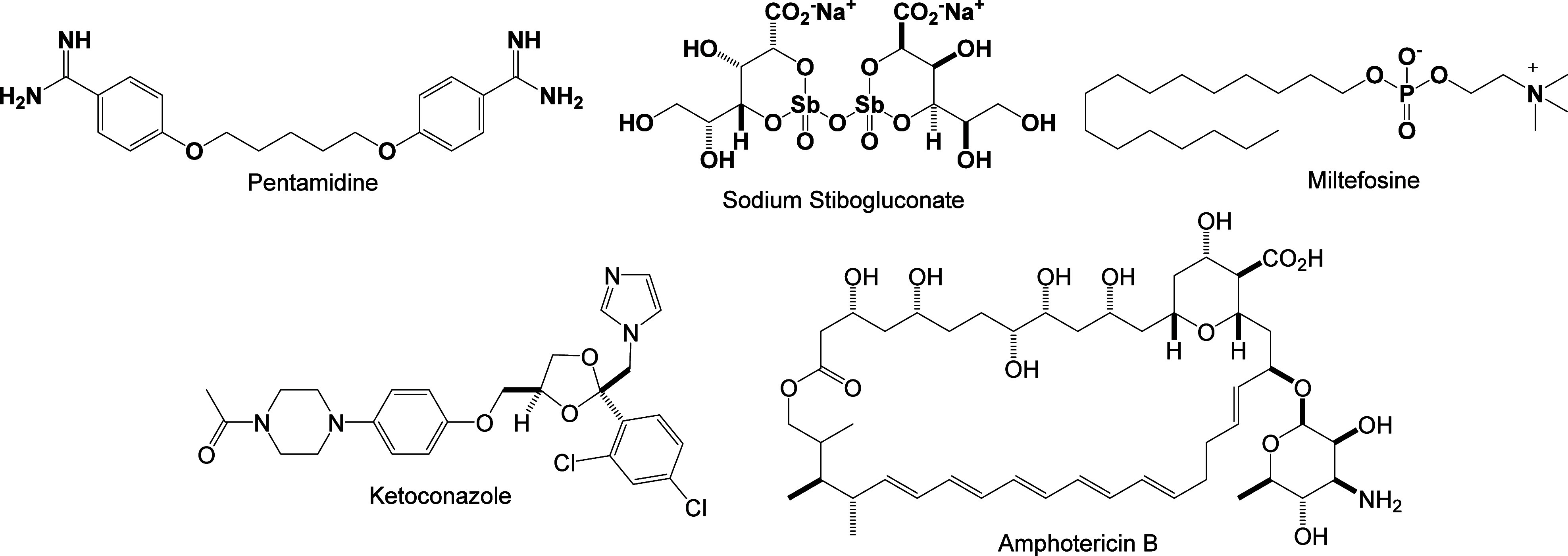
Chemical structures of the main approved drugs used in the treatment of leishmaniasis.

Efforts to design and develop several new antileishmanial agents under development in different phases of clinical trials are urgently needed to develop safer and more effective drugs. A wide range of heterocyclic compounds such as quinolines, chalcones, sulfur-containing products (thiazoles and thiosemicarbazones), coumarins, chromones, sulfonamides, pyrazoles, β-carbolines, indoles, and imidazoles have been explored toward the development of new therapeutic candidates.^8^ The triazole heterocycle has a significant place in medicinal chemistry. Due to the presence of three nitrogens (as 1,2,3- or 1,2,4-triazole) and other structural features, they have been explored to prepare compounds with significant leishmanicidal activity. The triazole derivatives that were investigated included triazole-lapachol and nor-lapachol hybrids,^9^ hydroquinone-triazole hybrids,^10^ triazole-containing amino acid derivatives,^11^ phthalimide-1,2,3-triazole derivatives,^12^ 1,2,4-triazole-3-thiol derivatives containing a triazole fused with pyrazine moiety,^13^ totarol-1,2,3-triazole derivatives,^14^ 1,2,3-triazolium salts,^15,16^ vanilline-triazole derivatives,^17,18^ and 2-nitroimidazole-1,2,3-triazole sulfonamide hybrids,^19^ with the latter showing the most promising antileishmanial activity among all (IC_50_ = 0.50 μM).

The bistriazoles are a promising class of heterocycles. Its versatility can be demonstrated by its use in the synthesis of new materials,^20–24^ for corrosion inhibitors,^25^ for metal-selective carbon paste electrodes (CPEs),^26,27^ and for the copper nanocluster-based metal–organic framework (CuMOF),^28^ as well as for biological investigation as antimicrobial,^29–34^ cytotoxic,^35–37^ antifungal,^38^ luminescent biosensors for DNA detection,^39^ AChE inhibitors,^40^ antidiabetic and antilipidemic,^41^ etc. Recently, we explored the syntheses of bistriazoles as antileishmanial compounds. The symmetrical 1,4-disubstituted 1,2,3-triazoles derivatives were synthesized via the CuAAC reaction using diazides as spacers and were used as leishmanicidal candidates. Among these compounds, the phenylic derivative 1 (Figure 2) was identified as the most active against promastigotes of *L. amazonensis* with an IC_50_ value of 63.3 μg mL^–1^ (182.9 μM).^42^ Others also observed the presence of an aromatic moiety as a favorable structural influence of leishmanicidal activity in N-substituted-phenyl-1,2,3-triazoles.^43^ Due to this, we decided to synthesize symmetrical 1,4-disubstituted 1,2,3-triazoles containing aromatic substituents, promote in silico studies of interaction to investigate docking energies, and evaluate biological essays against amastigotes and promastigotes forms of *Leishmania*.

**Figure 2:**
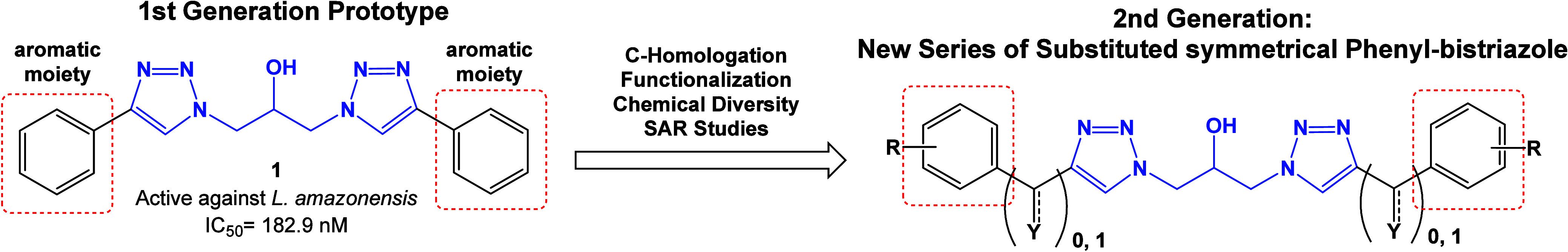
Design of a new series of phenylbistriazoles based on the structure of the leishmanicidal prototype bistriazole 1.

## 2 Results and Discussion

### 2.1 Chemistry

The synthesis of phenylic bistriazoles with a hydroxy-propane moiety as a spacer was planned to be performed following the CuAAC reaction strategy. Also, to evaluate the effect of a lack of symmetry, a simple phenylic triazole was also synthesized. In this direction, the reaction of benzyl azide 2 with a phenylic alkyne 3 mediated by CuSO_4_ in the presence of sodium ascorbate led to the known hydroxy-triazole 4, which was confirmed by the signal referring to the C_4_ and C_5_ of the triazole ring in the ^13^C NMR at 151.7 and 121.1 ppm, respectively. Despite the phenyl groups in the structure, it was not possible to determine the C_5_-H signal at the ^1^H NMR due to the overlapping of the signal in the same region. Then compound 4 was oxidized with MnO_2_ in 1,4-dioxane under reflux to furnish compound 5 in 60% yield over two steps (Scheme 1). The synthesis of the carbonyl derivative was confirmed by the IR band at 1634 cm^–1^ that referred to the C=O stretching and in the ^13^C NMR spectra by a new signal at 185.8 ppm that referred to the carbonyl group.

**Scheme 1:**
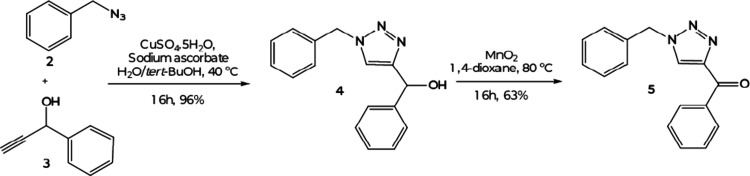
Synthetic Route for the Nonsymmetric Triazoles 4 and 5

Two different strategies were employed for the synthesis of phenylic bis­(triazoles) (Scheme 2). First, ethynyl aromatic alkynes 6a,b were prepared in two steps according to the literature^44^ and then coupled along commercially available 6c with the 1,3-diazo-2-hydroxy-propane (7) by use of standard CuAAC conditions, leading to bistriazoles 8a–c in good to excellent yields (81 to 95%). The preparation of 8a–c was confirmed by new ^1^H NMR signals of C_5_-H hydrogens of 1,2,3-triazole rings at the 8.81–8.49 ppm region. To synthesize phenylic bistriazole 9 containing a hydroxy-methylene spacer, the use of heterogeneous copper–carbon supported conditions^45^ was more appropriate to coupling 7 with alkyne 3, furnishing the desired product in 84% yield. In this case, due to a spacer among rings, the characteristic C_5_-H signal in the ^1^H NMR spectra appeared at 7.81 ppm.

**Scheme 2:**
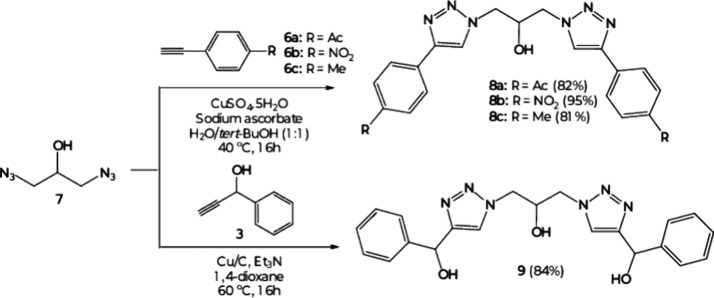
Synthetic Approach to the Symmetric Bistriazoles 8a–c and 9

To create chemical diversity, bistriazole 9 was employed as the substrate to synthesize new compounds. Due to this, selective oxidation with MnO_2_ led to the carbonyl bistriazole 10 in 84% yield. The carbonyl bistriazole 10 was used for the preparation of the oxime derivative 11 (49% yield), which was confirmed by the band at ∼1651 cm^–1^ that referred to the C=N stretching and by the signal at 148.2 ppm at the ^13^C NMR spectra that referred to the C=N group. For the syntheses of the hydrazones 12a–c, with yields ranging from 55 to 93%, different methodologies were applied (Scheme 3). The characterization of the compounds was confirmed due to the bands in the IR spectra at ∼1637 cm^–1^ for 12a, at ∼1670 cm^–1^ for 12b, and at ∼1606 cm^–1^ for 12c, which all referred to the C=N stretching.^46,47^ Since the compounds were synthesized without any diastereocontrol, both isomers were considered to be formed and characterized by the peaks at 153.9 and 153.8 ppm in the ^13^C NMR spectra that referred to the C=N group in both E/Z isomers for 12a. In the case of the hydrazones 12b and 12c, we could only see one signal at 161.2 and 167.7 ppm in the ^13^C NMR spectra, respectively, and the mixture of geometric isomers only can be suggested.

**Scheme 3:**
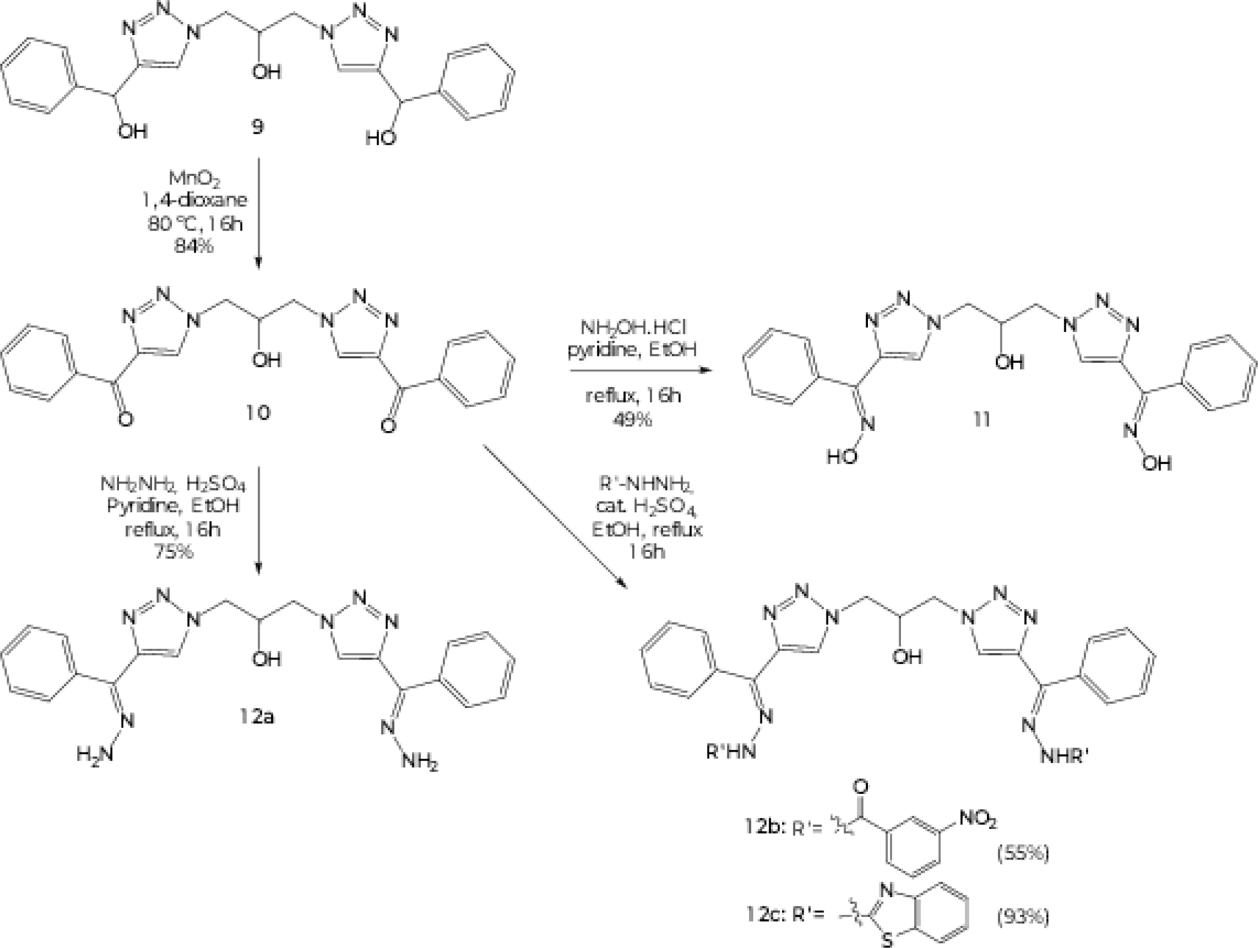
Synthetic Approach to the Symmetric Bistriazoles 10, 11, and 12a–c

### 2.2 Biological Evaluation

The synthetic nonsymmetric triazoles 4 and 5 and the symmetric bistriazole derivatives 8–12 were in vitro screened for the extracellular promastigote forms of *Leishmania amazonensis* compared to amphotericin B used as a standard drug. As depicted in Table 1, compound 12c (IC_50_ = 19.24 μM) showed the highest potency against the flagellate form of the parasite followed by compounds 12b (IC_50_ = 34.46 μM), 10 (IC_50_ = 44.13 μM), and 4 (IC_50_ = 42.81 μM). The cytotoxicity evaluation on murine macrophages revealed that the most active compounds were also significantly toxic with SI ∼2, except for compounds 4 and 10 that showed SI values of 7.51 and 8.69, respectively. Considering an SI higher than 8 as a selection criterion, only diketone-bistriazole derivative 10 was submitted to further evaluation against the intracellular amastigote form, showing a significant cytotoxic effect, with an IC_50_ value of 68.38 μM (SI = 5.61). To evaluate its potential toxicity on normal human cells, the most promising compound 10 was also assayed against the human fibroblast culture (sulforodamine B (SRB) test), showing a significantly smaller cytotoxicity (CC_50_ = 568.09 μM, SI = 8.3) in comparison to amphotericin B (CC_50_ = 22.95 μM, SI = 12.07).^48^

**Table 1:** Biological Data of In Vitro Evaluation of Triazole Derivatives 4, 5, and 8–12 against Promastigote and Amastigote Forms of *L. amazonensis*

comp	IC_50_ ± SD (μM) promastigote^a^	IC_50_ ± SD (μM) amastigote^a^	murine macrophage CC_50_ ± SD (μM)^a^	SI (murine) proma;ama	human fibroblasts CC_50_ ± SD (μM)	SI (human) proma;ama
4	42.81 ± 2.66	--	321.7 ± 10.41	7.51	--	--
5	N.A.	--	--	--	--	--
8a	N.A.	--		--	--	--
8b	48.38 ± 1.3	--	304.73 ± 7.84	6.29	--	--
8c	115.98 ± 2.61	--	282.2 ± 1.65	2.43	--	--
9	62.28 ± 3.29	--	144.27 ± 0.94	2.31	--	--
10	44.13 ± 2.36	68.38 ± 2.61	383.9 ± 3.21	8.69; 5.61	568.09 ± 2.58	12.87; 8.3
11	N.A.	--	--	--	--	--
12a	N.A.	--	--	--	--	--
12b	34.46 ± 0.28	N.A.	118.43 ± 17.15	3.43	--	--
12c	19.24 ± 1.40	N.A.	51.27 ± 8.57	2.66	--	--
amph B	1.40 ± 0.04	1.90 ± 0.07	27.10 ± 3.19	19.35; 14.26	22.95 ± 0.1	16.39; 12.07

a Analysis between the tested compounds by ANOVA (there is no significant difference (*p* < 0.05)). IC_50_: inhibitory concentration of 50% of promastigote and amastigote forms; CC_50_: 50% cytotoxicity concentration toward murine macrophages or human fibrobasts; SI (CC_50_/IC_50_): selectivity index; SD: standard deviation; -- not tested; N.A.= not active; amph B= amphotericin B.

Overall, these data highlight the effectiveness of compound 10 in inhibiting the growth and survival of both promastigote and amastigote forms of *L. amazonensis* at relatively low concentrations and with moderate cytotoxicity when compared to amphotericin B, one of the first-choice drugs for the clinics of leishmaniasis worldwide. Interestingly, a comparative structure–activity analysis among all tested compounds suggests that the bistriazole core is important for antileishmanicidal activity, as evidenced for four (8b, 10, 12b, and 12c) of the six most potent compounds. In addition, polar and H-bond donor substituents at the benzyl position, such as hydroxy, oxyme, and hydrazone (=NNH_2_, =NNHR) groups, seem to contribute to higher inhibitory potencies against promastigote forms but are unfavorable to the cytotoxicity toward the intracellular amastigote form as evidenced by the highlighted dual effect of the keto-analogue 10. The ability to act on both extracellular and intracellular forms of the parasite is a very relevant attribute for a new bioactive ligand as a promising drug candidate prototype for the treatment of leishmaniasis.

Considering that physicochemical properties, pharmacokinetic (PK) properties, and toxicity parameters are essential for the hit-to-lead process and the selection of potential new drug candidates,^49–51^ we also performed an in silico study with compound 10 to predict some key ADMET and PK parameters as depicted in Tables 2 and 3. The computational data suggested adequate physicochemical properties for compound 10, including good water solubility, adequate lipophilicity (clogP < 5), as well as no violations related to rotatable bonds and the number of H-bond acceptor/donor sites (Table 2).

**Table 2:** Physicochemical Properties of Compounds by the PKCSM Software^a^

	physicochemical properties					
compound	W.S.	M.W.	cLogP	R.B.	HB acceptors	HB donors
10	–3.999	402.414	1.3927	8	9	1

a W.S = water solubility; log mol/L (insoluble W.S < −10; poorly soluble −10 < W.S < −6; moderately soluble −6 < W.S < −4; soluble −4 < W.S < −2; very soluble −2 < W.S < 0; highly soluble W.S > 0.); M.W. = molecular weight; g/mol (acceptable range: <500); cLogP = high lipophilicity (expressed as LogP, acceptable range: <5); R.B = rotatable bounds (acceptable range: <10); H-bond acceptors (acceptable range: >10); and H-bond donors (acceptable range: >5).

**Table 3:** Predicted ADMET Properties of Compound 10 by the PKCSM Software^a^

ADMET properties	predicted value
CaCO-2 permeability	0.165
intestinal absorption (I.A.)	76.084
VDss	–0.632
CNS permeability	–3.869
total cleareance (T.C.)	0.416
AMES toxicity	no
DMT	0.603
hERG I inhibitor	no
hERG II inhibitor	yes
H.P.	yes

a CaCO-2 permeability; 10^–6^ cm/s (considered high: >0.90); I.A. = intestinal absorption; % (acceptable range: >30); VDss = steady state of distribution; log L/kg (considered high: >0.45); CNS permeability = central nervous system permeability; log PS (penetrate CNS: >−2); T.C. = total clearance; log mL/min/kg (acceptable range: <1.28); AMES toxicity (acceptable: no); DMT = maximum tolerated dose in humans; log mg/kg/day (considered high: >0.477); hERG I and II inhibitor (acceptable: no); H.P. = hepatotoxicity (acceptable: no).

On the other hand, despite its good antileishmanicidal effects, the prediction of ADMET properties of compound 10 suggested some unfavorable PK parameters that should be considered in a hit-to-lead optimization step (Table 3), such as poor cell permeability and low bioavailability expressed by the low steady state of distribution, besides the potential toxicity related to hERG II channel inhibition and hepatotoxicity.

### 2.3 Computational Studies

Docking calculations indicated that ligand 10 bound inside the active pocket from protein CYP51 with a binding energy of −9.96 kcal/mol. This negative energy signalizes the formation of a favorable complex, reinforced by the inhibition constant value of 50 nM, suggesting a high binding affinity at nanomolar concentrations. The ligand efficiency of 0.31 was also indicative of its stability and strong interactions considering its size and structure.

The key interacting residues between the ligand and the protein (Figure 3) predominantly involved Pi and van der Waals interactions, including ALA33, ILE41, PHE44, HIS137, and ILE119, suggesting that the stability of the complex is largely governed by interactions involving aromatic rings and nonpolar side chains. The Pi–Sigma interaction with ALA A:33 and ILE A:41 indicates that the ligand establishes strong contacts, contributing to a high affinity in the binding site.

**Figure 3:**
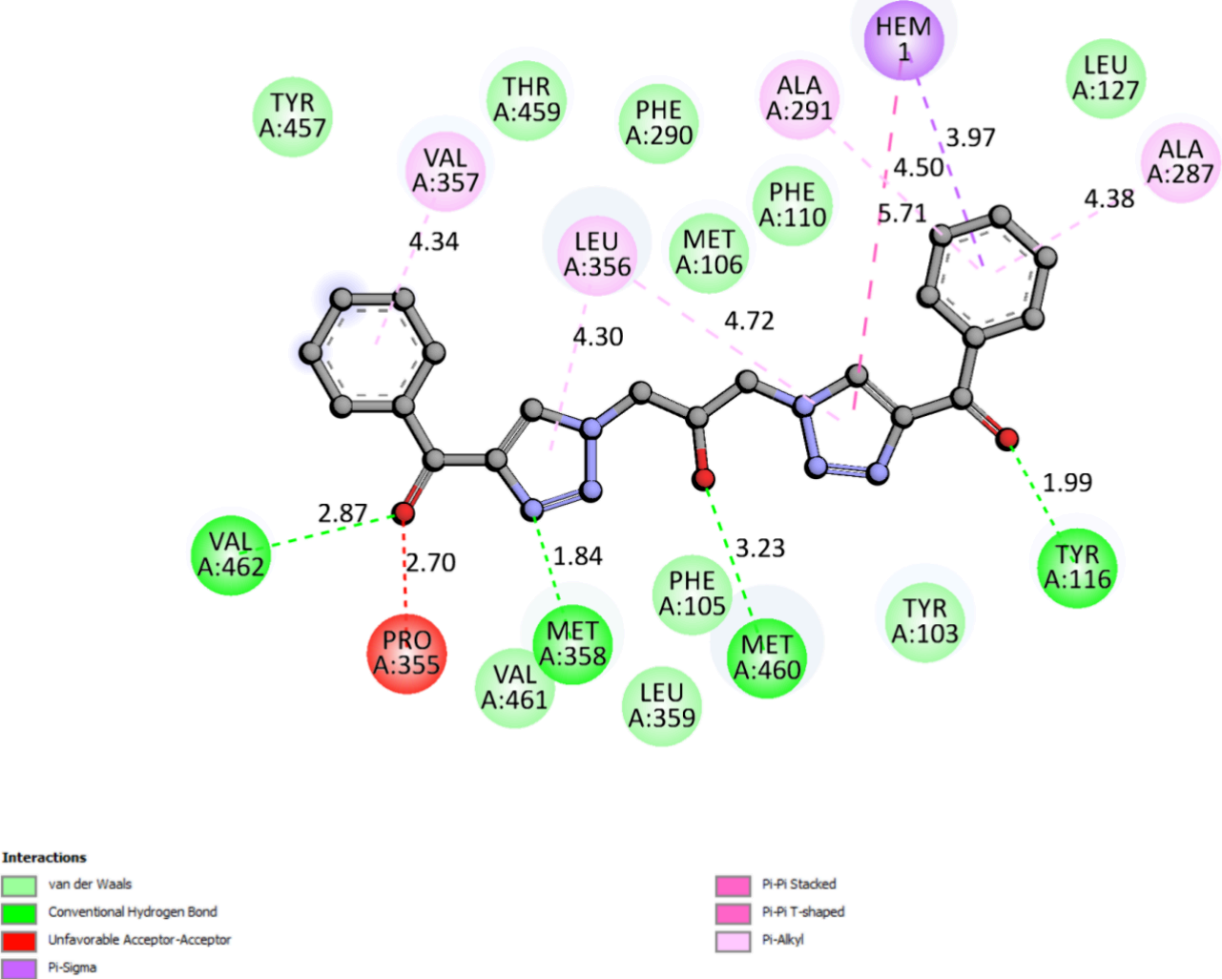
Two-dimensional interaction map for the complex between ligand 10 and CYP51 from *L. amazonensis*.

The residue ASP43 interacted through a Pi–anion interaction, which provided an additional electrostatic contribution to the stability of the complex. Pi–anion interactions typically involve charge transfer, which can be crucial for the conformational modulation of the protein.

Residues such as VAL214, LEU86, and LEU88 exhibited Pi–alkyl interactions, which are representative of hydrophobic interactions with aliphatic side chains. The presence of these interactions indicates that the ligand fits well into the hydrophobic regions of the protein, enhancing the stability of the complex. The van der Waals interactions involving residues PRO42, LEU88, ARG45, VAL181, VAL180, PHE183, and GLN117 suggest that the ligand also formed nonpolar contact regions. Although individually weak, these interactions, when combined, provide significant cooperative stabilization of the ligand–protein complex. The combination of Pi–Sigma, Pi–anion, Pi–alkyl, and van der Waals interactions provides a robust network of stabilizing interactions. Furthermore, the highlighted interactions suggest that the ligand occupies the binding site’s hydrophobic and aromatic regions. The addition of complementary charge interactions, as observed with ASP43 and HIS137, adds a level of specificity to the ligand’s affinity.

## 3 Conclusions

The search for new drugs that increase the therapeutic arsenal against leishmaniasis is one of the current challenges in medicinal chemistry. With the aim of expanding the list of compounds with leishmanicidal activity, a second generation of symmetrical 1,4-disubstituted 1,2,3-triazoles containing aromatic moieties was designed and synthesized from 2-hydroxy-1,3-bisazido-propane and terminal alkynes by copper­(I)-catalyzed alkyne–azide cycloaddition (CuAAC). The symmetrical bistriazoles were obtained in moderate to excellent yields (49 to 95%). All synthetic nonsymmetric triazoles and the symmetric bistriazole derivatives were in vitro screened for the extracellular promastigote forms of *Leishmania amazonensis*. From this investigation, it emerged that symmetric bistriazole 12c showed the highest potency against the flagellate form of the parasite followed by compounds 12b, 10, and 4. The cytotoxicity evaluation revealed that the most active compounds were also significantly toxic with SI ∼2, except for compounds 4 and 10 that showed SI values of 7.51 and 8.69, respectively. Considering SI > 8 as a selection criterion, only the dicarbonyl-bistriazole derivative 10 was submitted to further evaluation against the intracellular amastigote form, showing a significant cytotoxic effect, with an IC_50_ value of 68.38 μM (SI = 5.61). To evaluate its potential toxicity on normal human cells, the most promising compound 10 was also assayed against the human fibroblast culture, showing a significantly smaller cytotoxicity in comparison to amphotericin B. To correlate biological activity with a therapeutic target, in silico studies of the affinity of compound 10 with the active pocket from the protein CYP51 showed a binding energy of −9.96 kcal/mol, confirming the potential of compound 10 as a reference for studies of new compounds with leishmanicidal activity.

## 4 Experimental Details

### 4.1 Chemicals

All reagents were purchased from Sigma-Aldrich and Acros Chemicals and used without further purification. Dichloromethane was refluxed with CaH_2_ and distilled prior to use. Acetonitrile was used in HPLC grade. All reactions were performed under an argon atmosphere. Analytical thin layer chromatography (TLC) was performed on E. Merck TLC plates precoated with silica gel 60 F_254_ (250 μm thickness). Visualization was accomplished using UV light and potassium permanganate solution. Column chromatography was performed on a silica gel (60–120 Å pore size). The melting points were uncorrected and determined on an MQAPF-302 apparatus. IR spectra were measured using a Shimadzu IR-Affinity 1 spectrophotometer. Nuclear magnetic resonance spectra were recorded on Varian spectrometer mod. Inova-500, Bruker Avance DRX 300 MHz, and Bruker Avance III 400 in deuterated solvents, and chemical shifts are reported in ppm (d). Coupling constants (J) are reported in hertz. High-resolution mass spectra (HRMS) were obtained by electrospray using an Agilent 6550 Q-TOF MS instrument in positive mode.

### 4.2 Synthesis of the Series of Mono- and Bistriazole Derivatives

#### 4.2.1 Synthesis of Monotriazole Derivatives 4 and 5

##### 4.2.1.1 1-Benzyl-1*H*-1,2,3-triazol-4-yl)­(phenyl)­methanol (4)

Alkyne 3 (249.5 mg, 0.230 mL, 1.88 mmol) was added to benzyl azide 2 (214.0 mg, 1.61 mmol), and then the reaction mixture was added to 4 mL of distilled water. Then copper­(II) sulfate pentahydrate (12.0 mg, 0.05 mmol) and sodium ascorbate (17.1 mg, 0.1 mmol) were added. The reaction temperature was adjusted to 40 °C, and the reaction was followed by TLC. After 16 h, 30 mL of distilled water was added, and the mixture was extracted with ethyl acetate (3 × 10 mL). The organic combined layers were dried with magnesium sulfate and filtered. Finally, the solvent was evaporated by the rotatory evaporator, and hydroxy triazole 4 was obtained as a white solid (410.1 mg, 96%). Mp 125.5–128.3 °C (lit. data^52^ 120–123 °C); IR (KBr disc): 3236.5, 3165.2, 1543.1, 1496.8, 1450.5, 1122.6, 1047.4, 723.3, 694.4 cm^–1^; TLC rf 0.2 (eluent hexane/ethyl acetate 1:1); ^1^H NMR (300 MHz, CDCl_3_): δ 7.51–7.32 (m, 11H), 6.08 (d, *J* = 3.0 Hz, 1H), 5.55 (s, 2H), 2.94 ppm (d, *J* = 3.0 Hz, 1H); ^13^C NMR (75 MHz, CDCl_3_): δ 151.7, 141.9, 134.4, 129.0, 128.7, 128.5, 128.0, 127.9, 126.4, 121.0, 69.1, 54.2 ppm.

##### 4.2.1.2 1-Benzyl-1*H*-1,2,3-triazol-4-yl)­(phenyl)­methanone (5)

To a dry flask were added manganese dioxide (878.1 mg, 10.1 mmol) and a freshly prepared solution of alcohol (1.0 mmol) in 1,4-dioxane (4.00 mL). The reaction mixture was warmed to 80 °C and monitored by TLC until the total conversion of the starting materials. The mixture was purified in Celite to remove the manganese residue. Finally, the solvent was evaporated by a rotatory evaporator, and the crude product was purified by a chromatography column using a mixture of hexane/ethyl acetate as eluent to furnish a crystalline solid with 63% yield (165.8 mg). Mp 101–103 °C (lit. data^53^ 108–110 °C); IR (KBr disc): 3120.6, 3078.1, 1634.5, 1599.7, 1574.6, 1518.7, 1458.8, 1447.2, 1348.8, 1231.1, 1049.7, 903.0 cm^–1^; TLC Rf 0.3 (eluent hexane/ethyl acetate 1:1); ^1^H NMR (500 MHz, CDCl_3_): δ 8.34 (d, *J* = 7.4 Hz, 2H), 8.09 (s, 1H), 7.56–7.18 (m, 11H), 5.53 (s, 2H); ^13^C NMR (125 MHz, CDCl_3_): δ 185.6, 148.3, 136.4, 133.6, 133.2, 130.6, 129.3, 129.1, 128.4, 128.2, 54.4 ppm.

#### 4.2.2 General Procedure to Synthesize Symmetrical Bistriazoles 8a–c Using Copper­(II) Sulfate Pentahydrate

The CuAAC reaction of alkynes 6a–c (1.92 mmol) was carried out with the treatment of 1,3-diazidopropan-2-ol (7) (0.64 mmol), copper­(II) sulfate pentahydrate (4.8 mg, 0.019 mmol), and sodium ascorbate (38.0 mg, 0.192 mmol) in 4.0 mL of a 1:1 mixture of *tert*-butyl alcohol and distilled water. The reaction mixture was warmed to 40 °C and monitored by TLC until total conversion of the starting materials. At the end of the reaction, 30 mL of distilled water was added, and the reaction was extracted with ethyl acetate (3 × 10 mL); the organic combined layers were dried with dry magnesium sulfate and filtered. Finally, the solvent was evaporated by a rotatory evaporator.

##### 4.2.2.1 1,1’-(((2-Hydroxypropane-1,3-diyl)­bis­(1*H*-1,2,3-triazole-1,4-diyl))­bis­(4,1-phenylene))­bis­(ethan-1-one) (8a)

Yellow solid; yield 82% (225.8 mg); mp 257 °C (darkened); IR (KBr) 3385.1, 3132.4, 3099.6, 1672.3, 1612.5, 1570.1, 1458.2, 1271.1 cm^–1^; ^1^H NMR (300 MHz, DMSO-*d*_6_): δ 8.67 (s, 2H), 8.04–7.97 (m, 8H), 5.89 (d, *J* = 3.0 Hz, 1H), 4.68–4.65 (m, 2H), 4.46–4.43 (m, 3H), 2.58 (s, 6H); ^13^C NMR (75 MHz, DMSO-*d*_6_): δ 198.0, 145.7, 136.3, 135.5, 129.5, 125.6, 124.2, 68.7, 53.8, 27.1 ppm; HRMS (ESI) calculated for C_23_H_22_N_6_O_3_ [M + Na]+ 453.1651, found 453.1646.

##### 4.2.2.2 1,3-Bis­(4-(4-nitrophenyl)-1*H*-1,2,3-triazol-1-yl)­propan-2-ol (8b)

Yellow solid; yield 95% (265.2 mg); mp 262–264 °C; IR (KBr): 3414.0, 3136.2, 3105.4, 1604.8, 1519.9, 1462.0, 1354.0, 1334.7, 1234.4 cm^–1^; TLC Rf 0.4 (eluent ethyl acetate/methanol 9:1); ^1^H NMR (500 MHz, DMSO-*d*_6_): δ 8.81 (s, 2H), 8.31 (d, *J* = 8.8 Hz, 4H), 8.13 (d, *J* = 8.8 Hz, 4H), 5.85 (d, J= 5.0 Hz, 1H), 4.71–4.66 (m, 2H), 4.50–4.44 (m, 3H); ^13^C NMR (125 MHz, DMSO-*d*_6_): δ 147.0, 144.7, 137.7, 126.4, 125.1, 124.9, 68.6, 53.9 ppm; HRMS (ESI) calculated for C_19_H_16_N_8_O_5_ [M + Na]+ 291.0991, found 291.0987.

##### 4.2.2.3 1,3-Bis­(4-(*p*-tolyl)-1*H*-1,2,3-triazol-1-yl)­propan-2-ol (8c)

White solid; yield 81% (194.0 mg); mp 250–253 °C (lit. data^54^ 242–244 °C); IR (KBr disc): 3105.4, 3020.5, 1500.6, 1462.0, 1234.4, 1149.6, 1118.7, 817.8 cm^–1^; TLC Rf 0.3 (eluent hexanes/ethyl acetate 1:1); ^1^H NMR (300 MHz, DMSO-*d*_6_): δ 8.49 (s, 2H), 7.74 (d, *J* = 8.0 Hz, 4H), 7.24 (d, *J* = 8.0 Hz, 4H), 5.79 (s, 1H), 4.62–4.59 (m, 2H), 4.41–4.38 (m, 3H), 2.31 (s, 6H); ^13^C NMR (75 MHz, DMSO-*d*_6_): δ 146.2, 137.1, 129.5, 128.1, 125.1, 122.1, 68.3, 53.2, 20.8 ppm.

#### 4.2.3 Synthesis oF Symmetrical Bistriazoles 9 and 10

##### 4.2.3.1 ((2-Hydroxypropane-1,3-diyl)­bis­(1*H*-1,2,3-triazole-1,4-diyl))­bis­(phenylmethanol) (9)

To a flask were added the Cu/C catalyst (100 mg), 1,4-dioxane (4.0 mL), triethylamine (2.40 mmol), 1-phenylprop-2-yn-1-ole (2.40 mmol), and finally 1,3-diazidopropan-2-ole (1.12 mmol). The reaction mixture was warmed to 60 °C and stirred. The reaction was followed by TLC, and after 16 h, the reaction was cooled at room temperature. The crude product was purified first by Celite and sequentially by chromatography column using a mixture of hexane/ethyl acetate/ethanol as eluent to give the product as a yellow solid (395.7 mg, 87%); mp 25–28 °C; IR (KBr disc): 3290.6, 3144.0, 3063.0, 1894.1, 1824.7, 1770.6, 1550.8, 1492.9, 1454.3, 1384.9, 1226.7, 1049.3 cm^–1^; TLC Rf 0.6 (eluent ethyl acetate/methanol 9:1). ^1^H NMR (300 MHz, DMSO-*d*_6_): δ 7.81 (s, 2H), 7.38 (d, *J* = 7.2 Hz, 4H), 7.30 (t, *J* = 7,2 Hz, 4H), 7.24–7,19 (m, 2H), 5.96 (d, *J* = 4.3 Hz, 2H), 5.78 (d, *J* = 4.3 Hz, 2H), 5.62 (s, 1H), 4.43–4.38 (m, 2H), 4.26–4.22 ppm (m, 3H). ^13^C NMR (75 MHz, DMSO-*d*_6_): δ 151.3, 144.1, 128.1, 126.5, 122.9, 68.0, 52.9 ppm; HRMS (ESI) calculated for C_21_H_22_N_6_O_3_ [M + Na]+ 429.1651, found 429.1646.

##### 4.2.3.2 ((2-Hydroxypropane-1,3-diyl)­bis­(1*H*-1,2,3-triazole-1,4-diyl))­bis­(phenylmethanone) (10)

To synthesize compound 10, the same procedure applied to compound 5 was used. Yellow solid; yield 87% (349.9 mg); mp 142–144 °C; IR (KBr disc): 3406.3, 3140.1, 3066.8, 1649.1, 1637.6, 1598.9, 1518.0, 1489.1, 1180.4, cm^–1^; TLC Rf 0.7 (eluent ethyl acetate/methanol 9:1); ^1^H NMR (500 MHz, DMSO-*d*_6_): δ 8.83 (s, 2H), 8.22 (d, *J* = 7.2 Hz, 4H), 7.68 (t, *J* = 7.2 Hz, 2H), 7.57 (t, *J* = 7.7 Hz, 4H), 5.80 (d, *J* = 4.4 Hz, 1H), 4.75–4.71 (m, 2H), 4.52 – 4.49 ppm (m, 3H); ^13^C NMR (125 MHz, DMSO-*d*_6_): δ 185.2, 146.3, 136.7, 133.3, 131.0, 129.9, 128.6, 67.9, 53.3 ppm. HRMS (ESI) calcd for C_21_H_18_N_6_O_3_ [M + Na]+ 425.1338, found 425.1330.

#### 4.2.4 Synthesis of Oxime (11) and Hydrazone (12a)

The mixture of the ketone (185.1 mg, 0.46 mmol), NH_2_OH·HCl or NH_2_NH_2_·H_2_SO_4_ (1.22 mmol), pyridine (1.16 mmol for oxime 11 and 2.44 to hydrazone 12a), and ethyl alcohol 2,5 mL was heated under reflux overnight. The reaction mixture was diluted with crushed ice, and the precipitate was filtered and washed with cold ethanol to yield the product.

##### 4.2.4.1 ((2-Hydroxypropane-1,3-diyl)­bis­(1*H*-1,2,3-triazole-1,4-diyl))­bis­(phenylmethanone) dioxime (11)

White solid; yield 75% (149.1 mg); mp 118–121 °C; IR (KBr disc):): 3334.9, 3063.0, 1651.1, 1635.6, 1531.5, 1496.8, 1444.7, 1265.3, 1047.4 cm^–1^; ^1^H NMR (300 MHz, DMSO-*d*_6_): δ 12.07 (s, 2H), 8.87–8.85 (m, 2H), 7.63–7.62 (m, 3H), 7.46–7.42 (m, 7H), 5.76–5.73 (m, 1H), 4.69–4.38 (m, 5H); ^13^C NMR (75 MHz, DMSO-*d*_6_): δ 148.2, 147.6, 144.2, 137.4, 135.5, 130.0, 132.2, 129.2, 128.9, 128.6, 128.6, 127.8, 124.4, 68.4, 68.3, 53.1, 53.0 ppm; HRMS (ESI) calculated for C_21_H_20_N_8_O_3_ [M + Na]+ 455.1556, found 455.1548.

##### 4.2.4.2 1,3-Bis­(4-(hydrazineylidene­(phenyl)­methyl)-1*H*-1,2,3-triazol-1-yl)­propan-2-ol (12a)

Yellow solid; yield 48% (95.0 mg); mp 331 °C (darkened); IR (KBr disc): 3406.3, 3144.0, 3057.2, 1637.6, 1589.3, 1568.1, 1491.0, 1442.8, 1238.3 cm^–1^; ^1^H NMR (300 MHz, DMSO-*d*_6_): δ 8.62–8.24 (m, 2H), 7.83–7.44 (m, 10H), 5.75–5.66 (m, 1H), 4.82–4.40 ppm (m, 5H); ^13^C NMR (75 MHz, DMSO-*d*_6_): δ 153.9, 153.8, 146.0, 139.8, 137.0, 134.0, 131.5, 130.1, 129.9, 129.7, 129.6, 129.6, 129.5, 128.5, 128.4, 128.1, 127.1, 127.0, 68.7, 68.6, 53.9, 53.6 ppm; HRMS (ESI) calculated for C_21_H_22_N_10_O [M + Na]+ 453.1876, found 453.1668.

#### 4.2.5 General Procedure for the Synthesis of Hydrazones (12b–c)

To a flask were added ketone (0.25 mmol), hydrazide (0.62 mmol), a freshly prepared solution of sulfuric acid in ethanol (0.5 mol L^–1^, 0.1 mL, 0.05 mmol), and finally absolute ethyl alcohol (0.9 mL). The reaction mixture was warmed to reflux and stirred. The reaction was followed by TLC, and after 16 h, the mixture was cooled at room temperature, and the precipitate was filtered and washed with cold ethanol to give the product.

##### 4.2.5.1 *N*′,*N*‴-(((2-Hydroxypropane-1,3-diyl)­bis­(1*H*-1,2,3-triazole-1,4-diyl))­bis­(phenylmethaneylylidene))­di­(benzohydrazide) (12b)

Yellow solid; yield 55% (100.1 mg); mp 143–146 °C; IR (KBr disc): 3414.0, 3084.2, 1670.4, 1616.4, 1558.5, 1533.4, 1477.5, 1444.7, 1348.2, 1273.0 cm^–1^; ^1^H NMR (300 MHz, DMSO-*d*_6_): δ 13.75 (s, 2H), 8.85–8.70 (m, 2H), 8.48–8.23 (m, 6H), 7.90 (m, 2H), 7.68–7.53 (m, 10H), 5.77–5.76 (m, 1H), 4.78–4.56 (m, 5H); ^13^C NMR (75 MHz, DMSO-*d*_6_): δ 161.2, 143.0, 141.9, 136.9, 135.0, 133.6, 133.5, 131.2, 130.2, 128.9, 128.68, 68.2, 53.8 ppm; HRMS (ESI) calculated for C_35_H_30_N_12_O_3_ [M + Na]+ 751.2102, found 751.2095.

##### 4.2.5.2 1,3-Bis­(4-((2-(benzo­[*d*]­thiazol-2-yl)­hydrazineylidene)­(phenyl)­methyl)-1*H*-1,2,3-triazol-1-yl)­propan-2-ol (12c)

Yellow solid; yield 93% (161.9 mg); mp 252–254 °C; IR (KBr disc): 3136.2, 3057.2, 1606.7, 1556.6, 1494.8, 1442.7, 1276.9, 1163.1, 1111.0 cm^–1^; major diastereoisomer ^1^H NMR (300 MHz, DMSO-*d*_6_): δ 12.38 (s, 2H), 8.34 (s, 2H), 7.74–7.73 (m, 6H), 7.49–7.46 (m, 8H), 7.31 (t, *J* = 7.7 Hz, 2H), 7.12 (t, *J* = 7.7 Hz, 2H), 5.82 (s, 1H), 4.79–4.74 (m, 2H), 4,58–4.54 (m, 3H). Minor diastereoisomer ^1^H NMR (300 MHz, DMSO-*d*_6_): δ 12.38 (s, 2H), 8.65 (s, 2H), 7.60 (d, *J* = 8.1 Hz, 1H), 7.44–7.42 (m, 1H), 7.31 (t, *J* = 7.7 Hz, 2H), 7.12 (t, *J* = 7.7 Hz, 2H), 5.82 (s, 1H), 4.79–4.74 (m, 2H), 4,58–4.54 (m, 3H); ^13^C NMR (75 MHz, DMSO-*d*_6_): δ 168.2, 129.9, 129.6, 128.8, 126.7, 125.3, 122.4, 121.9, 68.6, 53.8, 53.5 ppm; HRMS (ESI) calculated for C_35_H_28_N_12_O_3_S_2_ [M + Na]+ 719.1848, found 719.1838.

Characterization data (^1^H NMR, ^13^C NMR, and infrared spectra) for compounds (4–12c) are provided in the Supporting Information (Figures 1S–48S).

### 4.3 Biological Assays

#### 4.3.1 Cytotoxicity

Murine cytotoxicity was assessed by the 3-(4,5-dimethylthiazol-2-yl)-2,5-diphenyltetrazolium bromide (MTT) method using murine peritoneal macrophages to determine the CC_50_.^55^ For this purpose, murine peritoneal macrophages were maintained in RPMI 1640 medium at 37 °C and 5% CO_2_, arranged in 96-well plates at a ratio of 8 × 10^6^ per well, where the compounds to be evaluated were added at concentrations of 500, 250, 125, 62.5, 31.25, 15.625, 7.8125, and 3.90 μg mL^–1^ and incubated for 24, 48, and 72 h. After incubation, 10 μL of MTT (at a concentration of 5 mg mL^–1^) was added per well, with a further 4 h incubation. The cells were lysed with dimethyl sulfoxide (DMSO) and evaluated in a Shimadzu UV/vis spectrophotometer (double beam, model 2550) at 570 nm to determine the CC_50_ compared to the control without the addition of compounds or drugs.^56,57^ The optical density obtained was then converted to the percentage of inhibition using eq 1 so that the CC_50_ could then be determined by regression analysis of the data.

%inhibition=[(DOcontrol−DOcompound)/DOcontrol]×100 1

The percentage of viable cells was calculated considering the cell culture control (medium + cells + DMSO = 0.6% v/v).

#### 4.3.2 Leishmanicidal Activity against Promastigote Forms of *L. amazonensis*

Promastigotes of *L. amazonensis* (MHOM/BR/71973/M2269) were grown on 24-well plates in Schneider’s *Drosophila* medium (Sigma, USA) supplemented with 10.0% (v/v) heat-inactivated fetal bovine serum and 1.0% penicillin (10000 UI mL^–1^)/streptomycin (10.0 mg mL^–1^) (Sigma, USA). Cells were harvested in the log phase, suspended in a fresh medium, counted in Neubauer’s chamber, and adjusted to a concentration of 1 × 106 cells mL^–1^ using 24-well plates. The compounds were added to promastigote cultures (1 × 10^6^ cells mL^–1^) in the range of 0.10–40.00 μg mL^–1^, solubilized in dimethyl sulfoxide (DMSO) (0.6%, v/v in all wells), and incubated at 25 °C. After 72 h of incubation, resazurin was used to detect cellular metabolic activity for the determination of 50.0% inhibitory growth concentration (IC_50_-promastigote). All tests were performed in triplicate at three different times, and amphotericin B (Sigma) was used as the reference drug.^57^

#### 4.3.3 Leishmanicidal Activity against Amastigote Forms of *L. amazonensis*

To evaluate the leishmanicidal activity in amastigotes, murine peritoneal macrophages were used and maintained in RPMI-1640 medium at 37 °C and 5% CO_2_ in 24-well plates. After 24 h, the nonadherent macrophages were removed, and these cells were infected with *Leishmania (L.) amazonensis* promastigotes in the stationary phase of the proliferation curve at a ratio of 10:1. The day after infection, the noninternalized parasites were removed, and the compounds to be evaluated were added at concentrations of 40, 10, 5, and 0.1 μg mL^–1^ and incubated for 72 h at 37 °C and 5% CO_2_; they were then fixed in methanol and stained with 10% Giemsa (MERCK) for subsequent analysis by light microscopy. To assess the effect of the compounds, the number of amastigotes/macrophages and the number of infected macrophages were evaluated. The percentage of infected cells was considered to illustrate the results since these are expressed as CI_50_-amastigote.^55,57^

#### 4.3.4 Computational Experiments

To explain the possible mechanism of action of ligand 10, we used the ligand structure to predict its possible target in *L. amazonensis*. For this, we selected the amino acid sequence of the sterol 14-alpha demethylase (CYP51) from *Leishmania amazonensis* (Uniprot ID Q2HWD2) once it has been described as a drug target for CYP51 in different eukaryotic microorganisms including fungi^58,59^ and also the azoles as potent molecules against CYP51 in *Trypanosoma* species.^60,61^ The sequence was then submitted to SwissModel server^62^ for 3D homology modeling. After template searching, we selected the PDB structure 3L4D from *Leishmania infantum* ^63^ as the best one, as it conserves 97.32% identity and 99% coverture with *L. amazonensis* CYP51. In addition, we restricted our template selection as crystallographic with resolution below 3Å and R-free ≥ 0.2, as well as the presence of a cocrystallized ligand. In addition, this is the only *Leishmania* CYP51 crystallographic structure available on the PDB. The active site for docking calculations was defined based on the template reference.

Ligand 10 was prepared for docking by converting its 3D structure from sdf to pdbqt using the Autodock Raccoon plugin.^64^ Then, the protein receptor was prepared with Autodock Tools^65^ by running Autogrid to generate its corresponding docking grid map (glg) with the active site positioning, and the docking parameter file (dpf) was adjusted for using the Lamarckian Genetic Algorithm with 25,000 evaluations and 50,000 generations. In addition, the receptor file was saved in a pdbqt format. In a second step, the ligand and receptor were subjected to docking calculations with the Autodock 4.2,^65^ and the estimated affinity energies were ranked by their negative values. Finally, the inhibition constant (KI) was predicted as well as the ligand positioning inside the protein active site. The 2D interaction map was built using Discovery Studio.^66^

## Supplementary Material



## Data Availability

All data generated or analyzed during this study are included in this published article.

## Notes

Experimental procedures for the syntheses of compounds 2, 6a and b, and 7 and IR, ^1^H, and ^13^C NMR spectra of synthetic compounds (4–12c) (PDF)
