# Supplementary material for: Design, Synthesis, and In Vitro Evaluation of the Leishmanicidal Activity of New Aromatic Symmetrical 1,4-Disubstituted 1,2,3-Bistriazoles
Source: ACS Omega. 2025 May 12;10(26):27819–29. doi: 10.1021/acsomega.5c00492 (PMC12242654; doi:10.1021/acsomega.5c00492)
Supplement: Supplementary file 1 [file ao5c00492_si_001.pdf]

Supporting Information

Design, Synthesis, and In Vitro Evaluation of the Leishmanicidal Activity of New Aromatic Symmetrical 1,4-Disubstituted-1,2,3-Bistriazoles

Maurício Moraes Victor[a,b]*, Gabriel dos Santos Ramos[a,b], Bruno Silva Andrade[c], Patrícia Ferreira Espuri Sepin[d], Guilherme Álvaro Ferreira-Silva[d], Marisa Ionta[d], Amanda Almeida Morais[e], Vanessa Silva Gontijo[e], Claudio Viegas Jr.[e]*, and Marcos José Marques[d]

[a]Department of Organic Chemistry, Chemistry Institute, Federal University of Bahia, Salvador 40170-115, BA, Brazil

[b]National Institute of Science and Technology for Energy and Environmental, Salvador 40170-115, BA, Brazil

[c]Department of Biological Sciences, State University of Southwest of Bahia, Jequié 45208-091, BA, Brazil

[d]Institute of Biomedical Sciences, Federal University of Alfenas, Alfenas 37130-001, MG, Brazil

[e]PeQuim – Laboratory of Research in Medicinal Chemistry, Institute of Chemistry, Federal University of Alfenas, Alfenas 37133-840, MG, Brazil

*Author to whom correspondence should be addressed.

E-mail: mmvictor@ufba.br, and cvjviegas@gmail.com

Table of contents

1. Experimental procedures S2-S4
2. References S5
3. Spectra S6-S53

1. Experimental procedures:

1.1 - Benzyl azide 2 – ($C_7H_7N_3$)

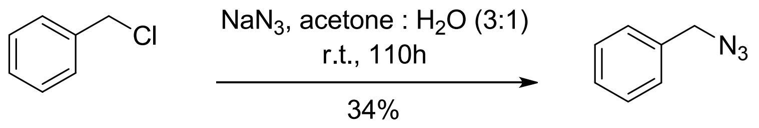

To a solution of sodium azide (3.04 g, 46.8 mmol) in (3:1 acetone/water, 90 mL) was added dropwise benzyl chloride (2.8 mL, 3.08 g, 24.3 mmol). The reaction mixture was stirred at room temperature for 110 h, diluted with water, and extracted with ethyl acetate (6 X 15 mL). The combined layers were washed with brine (15 mL), dried over anhydrous sodium sulfate, and concentrated in vacuum to give benzyl azide 2 (1.2 g, 35%) as crude light-yellow oil.

IR (film): 2088.7 cm^{-1}; rf 0.8 (eluent hexanes/ethyl acetate 1:1).[1]

1.2 - General procedure for syntheses of alkynes 6a and 6b

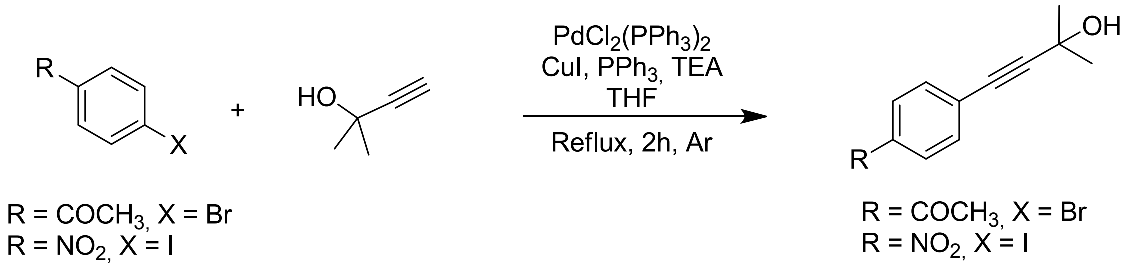

To a dry flask connected to a condenser were added aryl halide (11.07 mmol), $PdCl_2(PPh_3)_2$ (163.3 mg, 0.225 mmol, 2 mol%), triphenylphosphine (159.3 mg, 0.576 mmol), copper (I) iodide (46.6 mg, 0.229 mmol), triethylamine (6.5, 44.28 mmol) and dry THF (28 mL). The reaction mixture was warmed to 50 °C and stirred in argon atmosphere. After 15 minutes drop-to-drop was added a solution of mebynol (2.3 mL, 22.14 mmol) in 2 mL of THF, and the reaction mixture was warmed to reflux and protected from the light. The reaction was followed by TLC, and after 2 hours, the solvent evaporated on the rotatory evaporator. The crude product was purified in a chromatography column using hexane/ethyl acetate as eluent, an analytical sample was separated and the alkynol was used immediately in the next step [R = $COCH_3$: ^1H NMR (300 MHz, $CDCl_3$): δ 7.90 – 7.87 (d, J= 8.2 Hz, 2H), 7.49 – 7-47 (d, J= 8.2 Hz, 2H), 2.59 (s, 3H), 2.35 (s, 1H) e 1.63 (s, 6H); ^{13}C NMR (75 MHz, $CDCl_3$): δ 197.4, 136.2, 131.7,

128.2, 127.7, 97.1, 81.4, 65.6, 31.3, 26.6; R = NO_2: ^{1}H NMR (500 MHz, $CDCl_3$): δ 8.16 (d, J= 8.7 Hz, 2H), 7.55 Hz (d, J= 8.7 Hz, 2H), 2.44 (s, 1H), 1.64 (s, 6H); ^{13}C NMR (125 MHz, $CDCl_3$): δ 147.1, 132.4, 129.8, 123.5, 99.2, 80.4, 65.6, 31.2. To a dry flask connected to a condenser potassium hydroxide were added (296.8 mg, 4.1 mmol), potassium phosphate (1.35g, 6.4 mmol), and dry toluene (10 mL). After some minutes was added a freshly prepared solution of the alkynol (5.49 mmol) in dry toluene (4.0 mL). The system was covered with aluminum foil, heated to reflux, and stirred in an argon atmosphere. The reaction was followed by TLC, and after 30 minutes, the reaction mixture was cooled at room temperature and washed with a hydrochloric acid solution 5% (3 x 10 mL) and distilled water (3 x 10 mL). The organic layers were combined and dried with magnesium sulfate, and the solvent was evaporated on a rotatory evaporator to give the product.

1.2.1- 1-(4-Ethynylphenyl)etanone (6a) – ($C_{10}H_8O$)

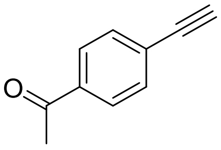

Yield: 75% (443.3 mg). IR (KBr): 3218.0, 2102.0 cm^{-1}; rf 0.7 (eluent hexanes/ethyl acetate 1:1).[2]

1.2.2 - 1-Ethynyl-4-nitrobenzene (6b) – ($C_8H_5NO_2$)

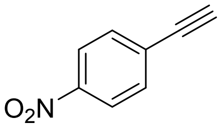

Yield: 53% (427.8 mg), IR (KBr): 3252.0, 2106.3 cm^{-1}; rf 0.5 (eluent: hexanes/ethyl acetate 1:1).[3]

1.2.3 - 1,3-Diazidopropan-2-ol (7) ($C_3H_6N_6O$)

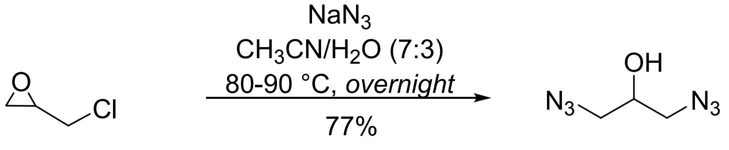

To a stirred solution of epichlorohydrin (2.36 g, 2.00 mL, 25.5 mmol) in a mixture of acetonitrile and water (70:30) sodium azide (4.02 g, 61.6 mmol) was added at room temperature and the mixture was refluxed overnight at 90 °C. The solvent was removed under reduced pressure, and the aqueous layer was extracted with ethyl acetate (3 X 10 mL). The combined organic layer was dried over anhydrous sodium sulfate. The pure compound (20.9 mmol) was obtained after removing the solvent under reduced pressure as a transparent viscous liquid. The crude product was purified by chromatography column using a mixture of hexanes/ethyl acetate as eluent to yield 2.8g (77%) of 1,3-diazidopropan-2-ol (7).

IR (film): 2085.0 cm^{-1}; rf 0.8 (eluent: hexanes/ethyl acetate 1:1).[4]

2 - References

1. Rono CK, Darkwa J, Meyer D, Makhubela BCE. A Novel Series of N-aryltriazole and N-acridinyltriazole Hybrids as Potential Anticancer Agents. Curr Org Synth. 2019;16(6):900-912. doi:10.2174/1570179416666190704112904

2. Gallagher WP, Maleczka RE. PMHS-mediated couplings of alkynes or benzothiazoles with various electrophiles: Application to the synthesis of (-)-akolactone A. J Org Chem. 2003;68(17):6775-6779. doi:10.1021/jo034463+

3. Wang S, Li Y, Liu H, et al. Topochemical polymerization of unsymmetrical aryldiacetylene supramolecules with nitrophenyl substituents utilizing C-H···π interactions. Org Biomol Chem. 2015;13(19):5467-5474. doi:10.1039/c5ob00435g

4. Priyanka KG, Mishra AK, Kantheti S, Narayan R, Raju KVSN. Synthesis of Triazole Ring-Containing Pentol Chain Extender and Its Effect on the Properties of Hyperbranched Polyurethane-Urea Coatings. J A Polym Scien. 2012;126:2024-2034.

3 - Spectra

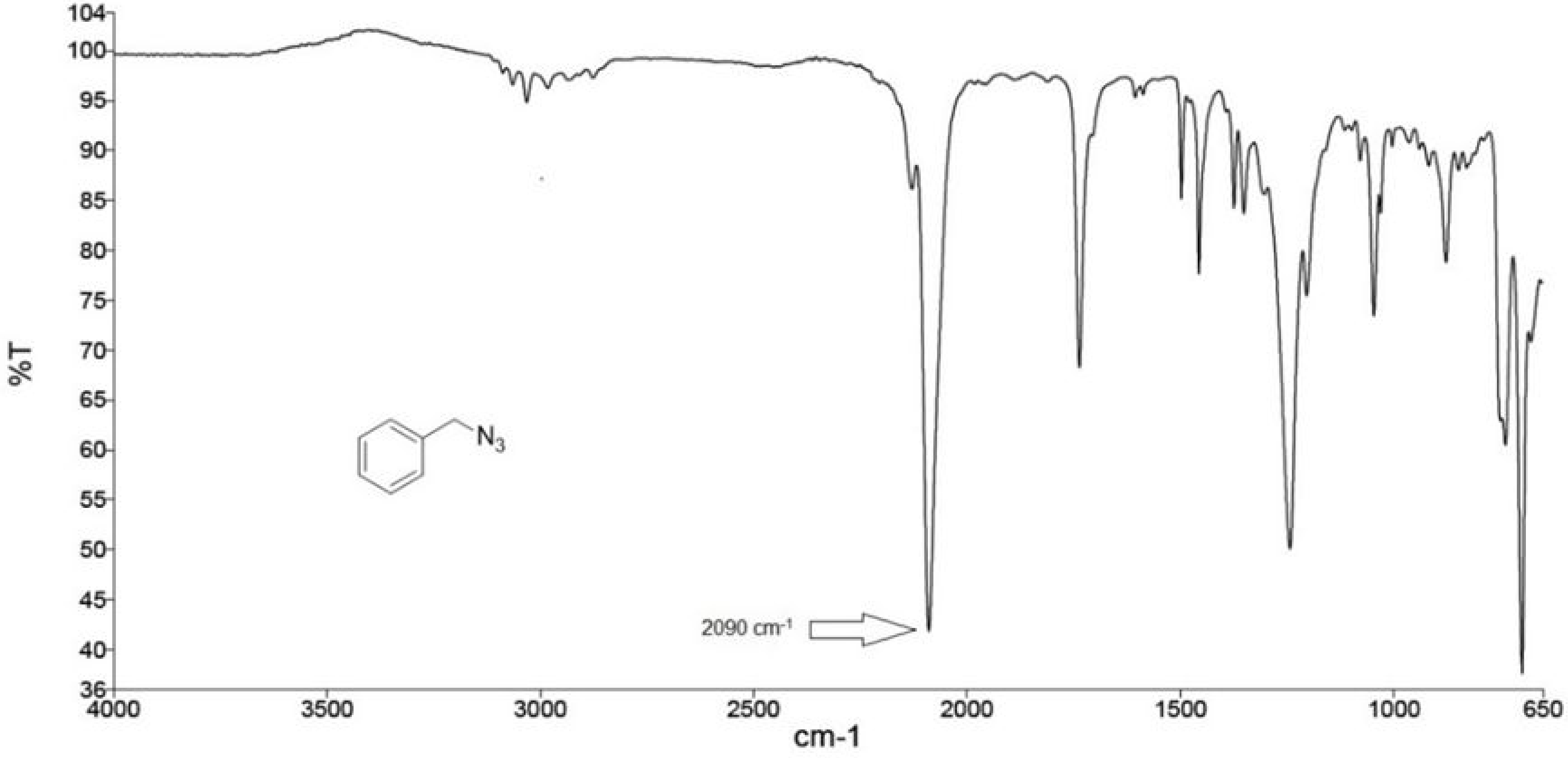

Figure 1S. FT-IR spectra (ATR) of 2.

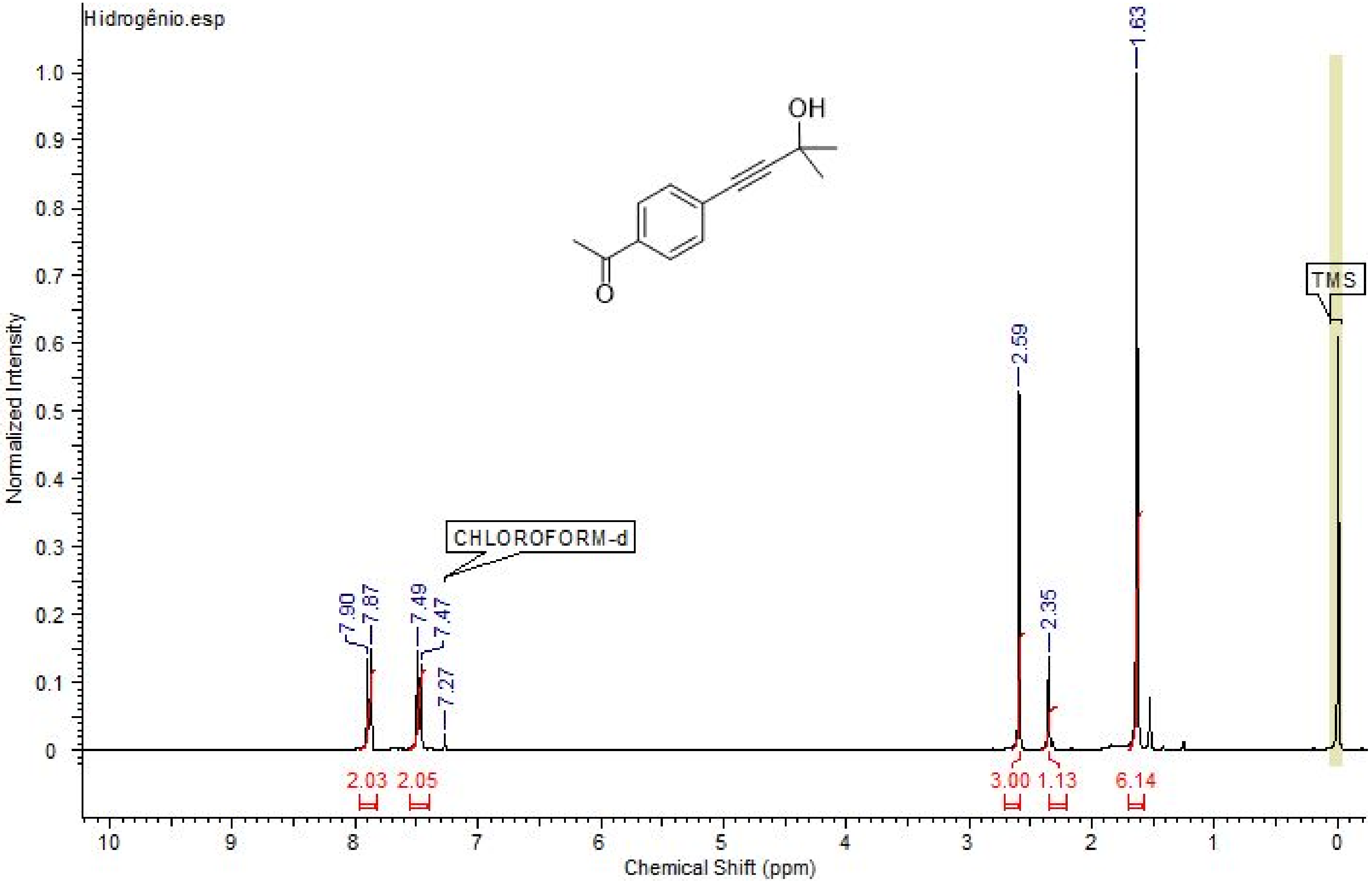

Figure 2S. ^{1}H NMR spectra (300 MHz, $CDCl_3$) of intermediate to the synthesis of 6a.

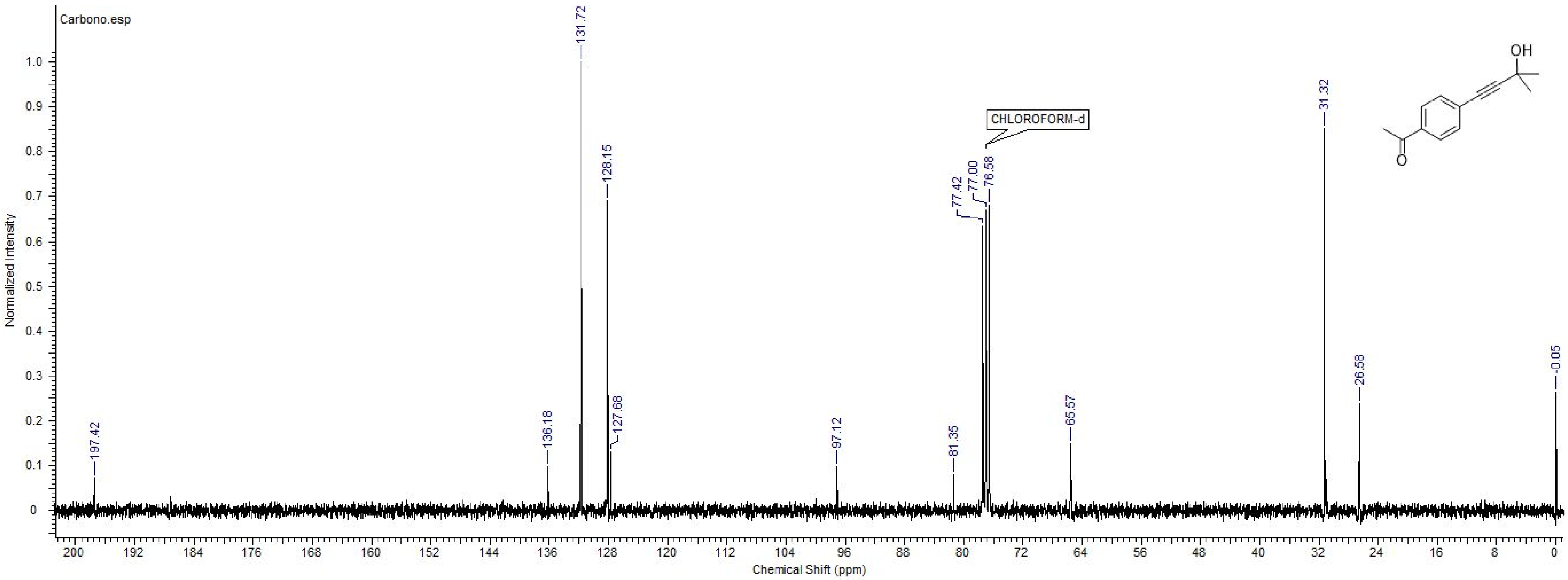

Figure 3S. ^{13}C NMR spectra (75 MHz, $CDCl_3$) of intermediate to the synthesis of 6a.

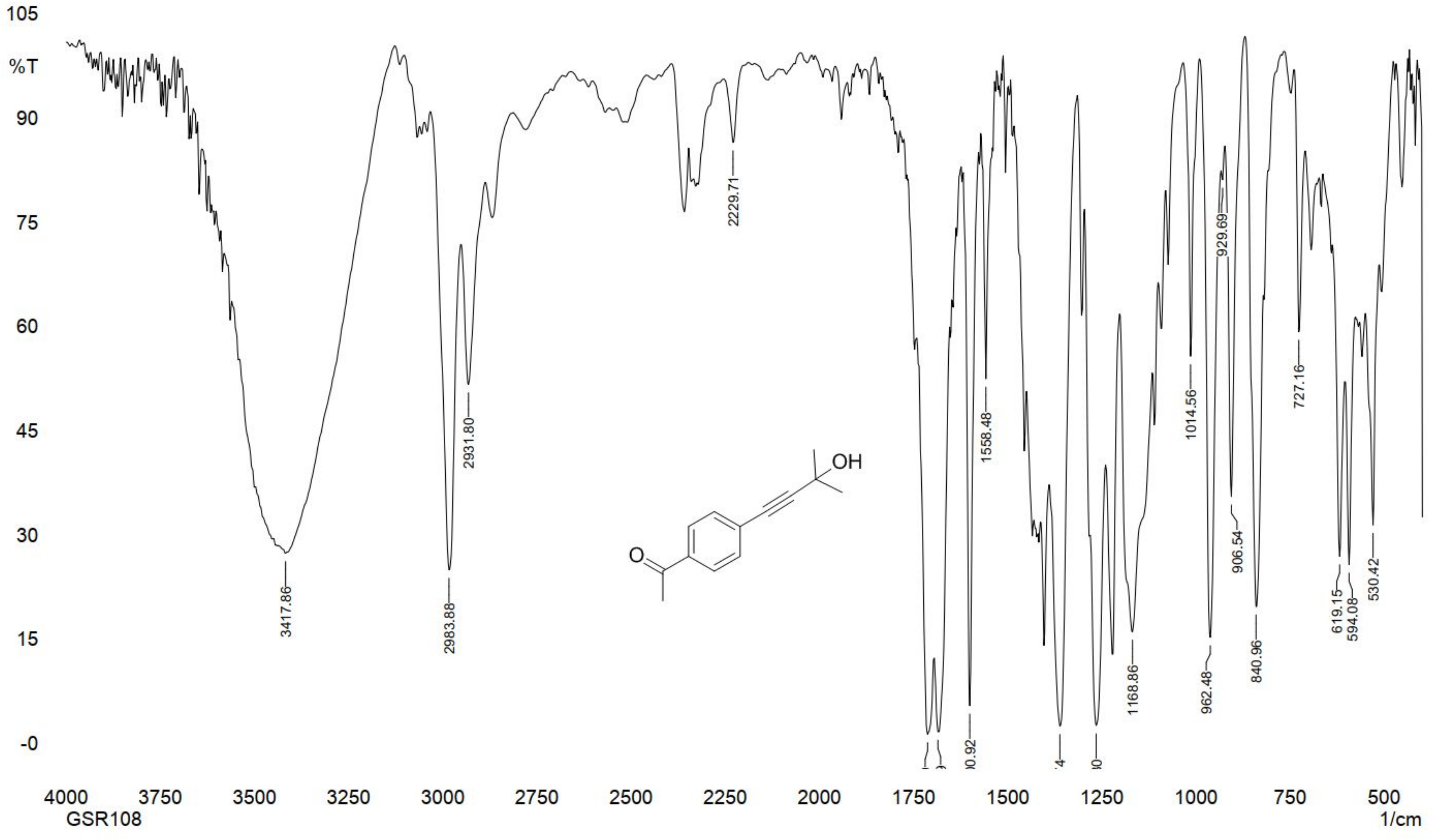

Figure 4S. FT-IR spectra (KBr disk) of intermediate to the synthesis of 6a.

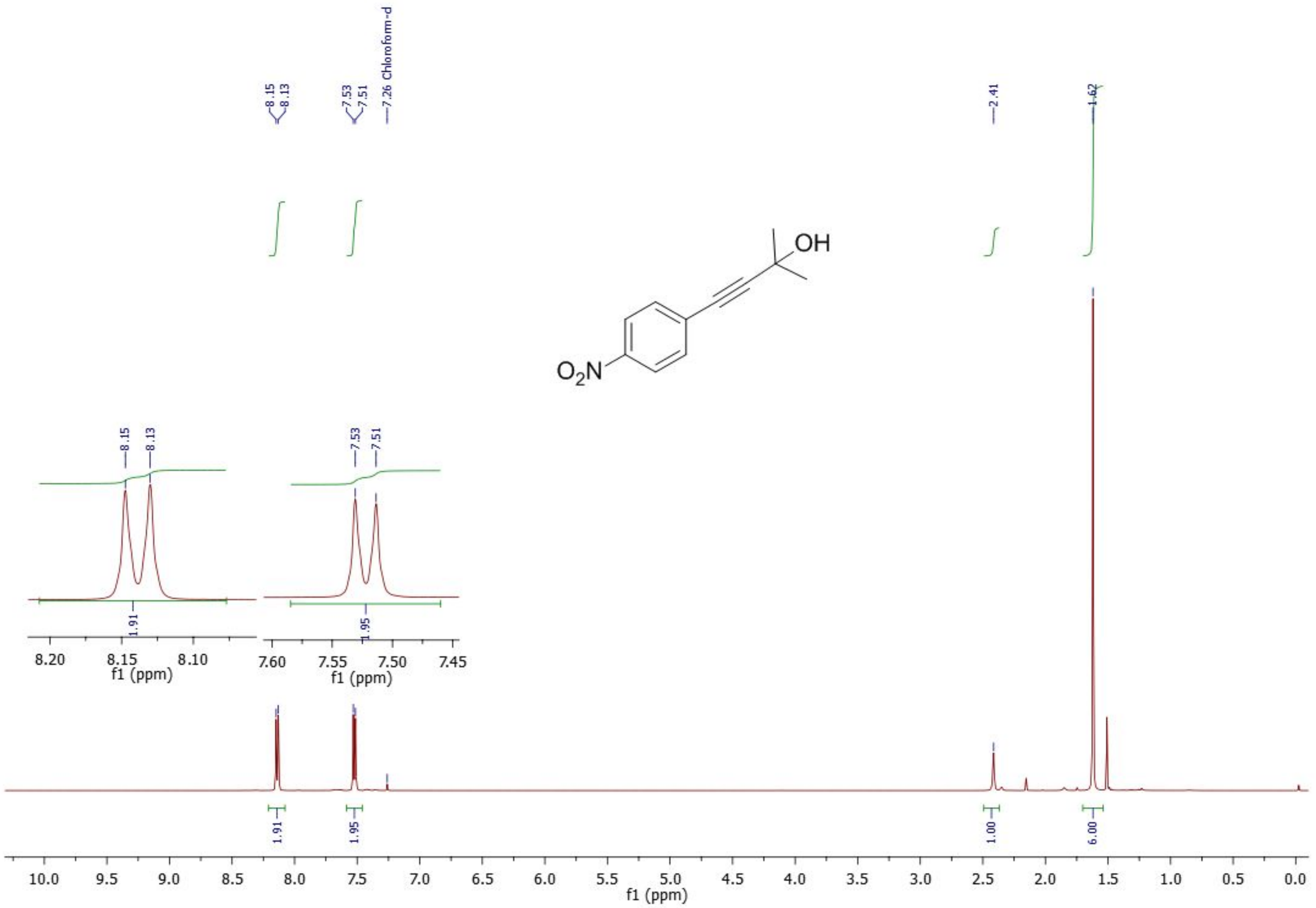

Figure 5S. ^{1}H NMR spectra (500 MHz, $CDCl_3$) of intermediate to the synthesis of 6b.

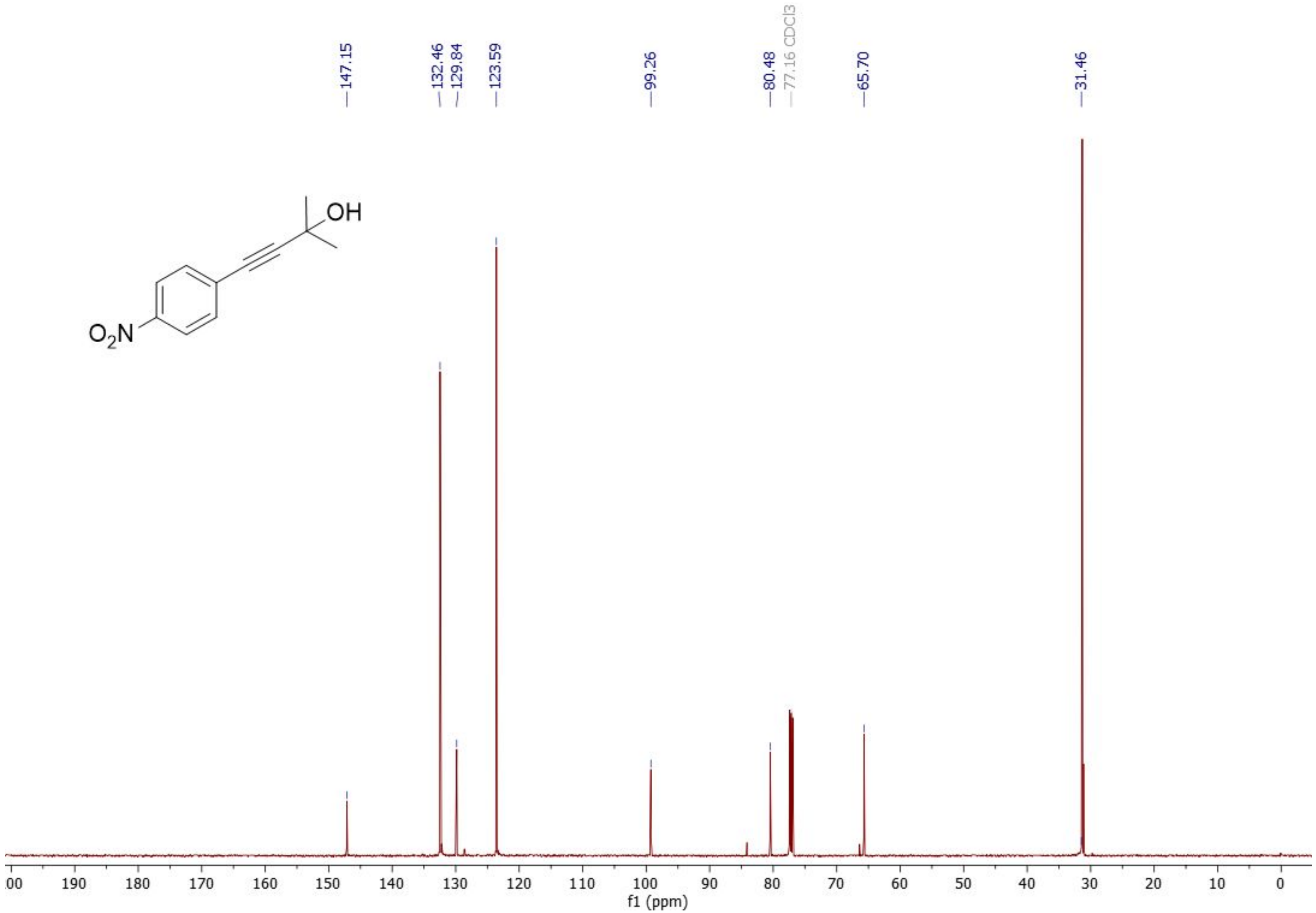

Figure 6S. ^{13}C NMR spectra (125 MHz, $CDCl_3$) of intermediate to the synthesis of 6b.

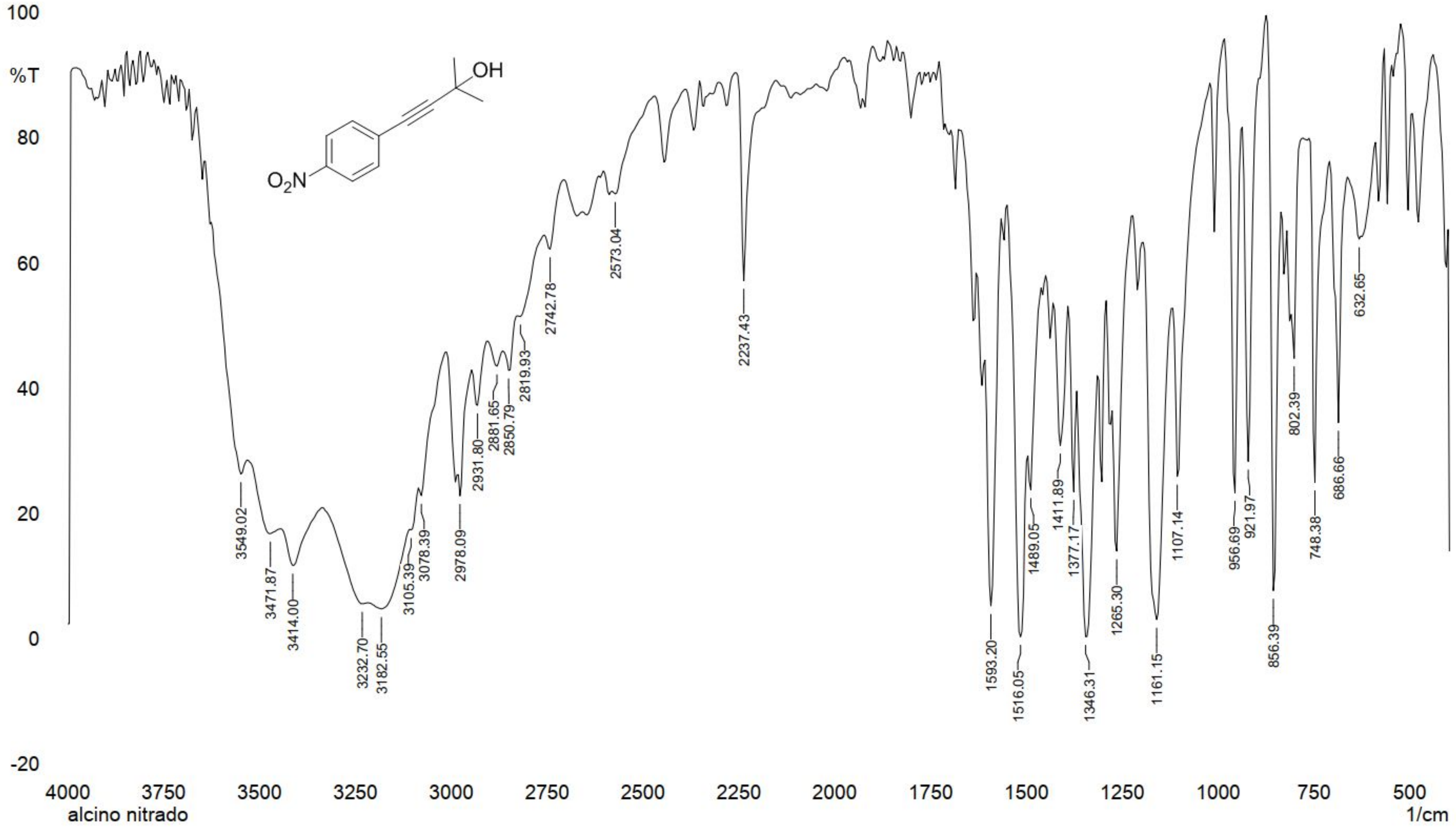

Figure 7S. FT-IR spectra (KBr disk) of intermediate to the synthesis of 6b.

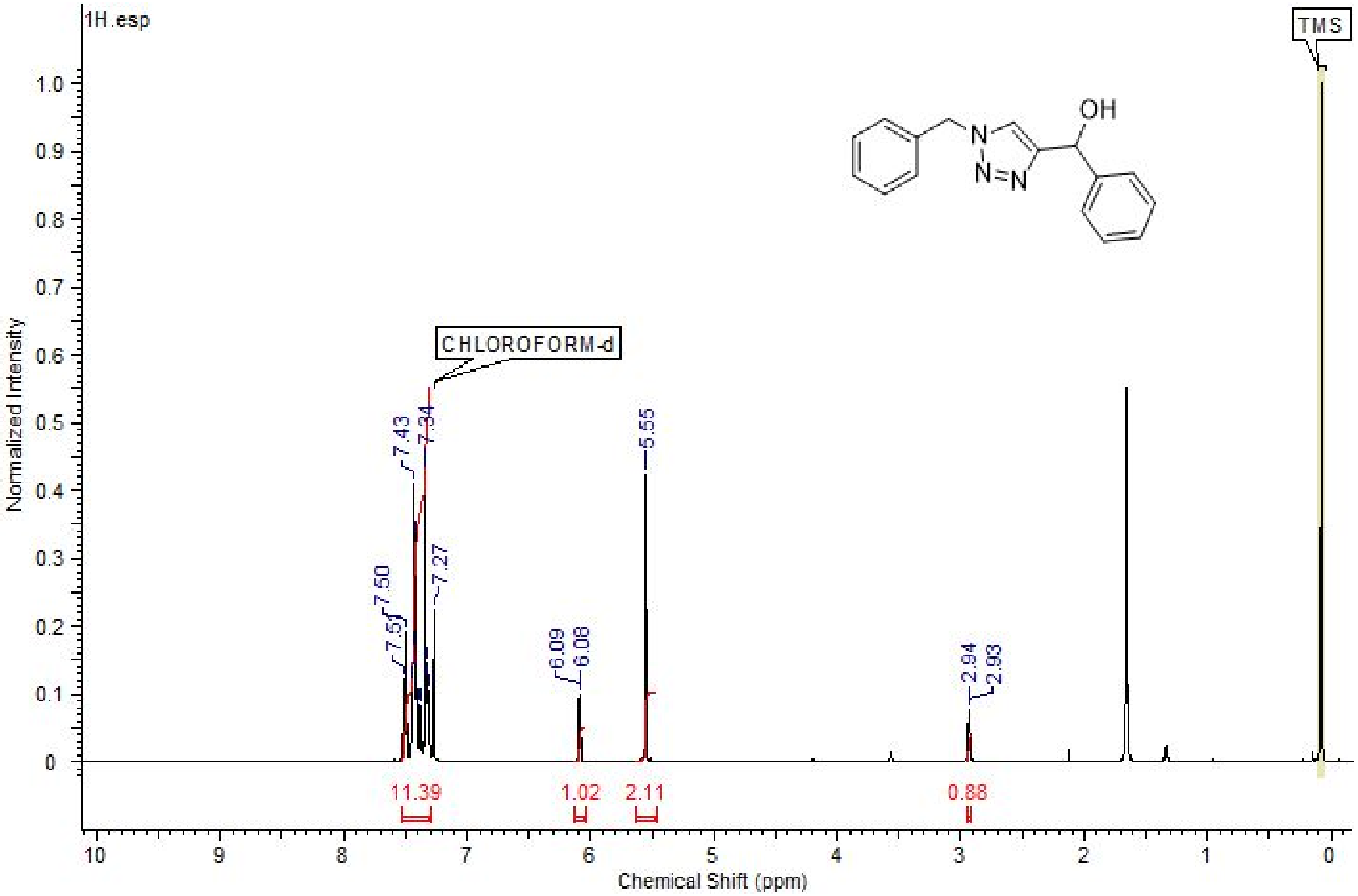

Figure 8S. ^{1}H NMR spectra (300 MHz, $CDCl_3$) of 4.

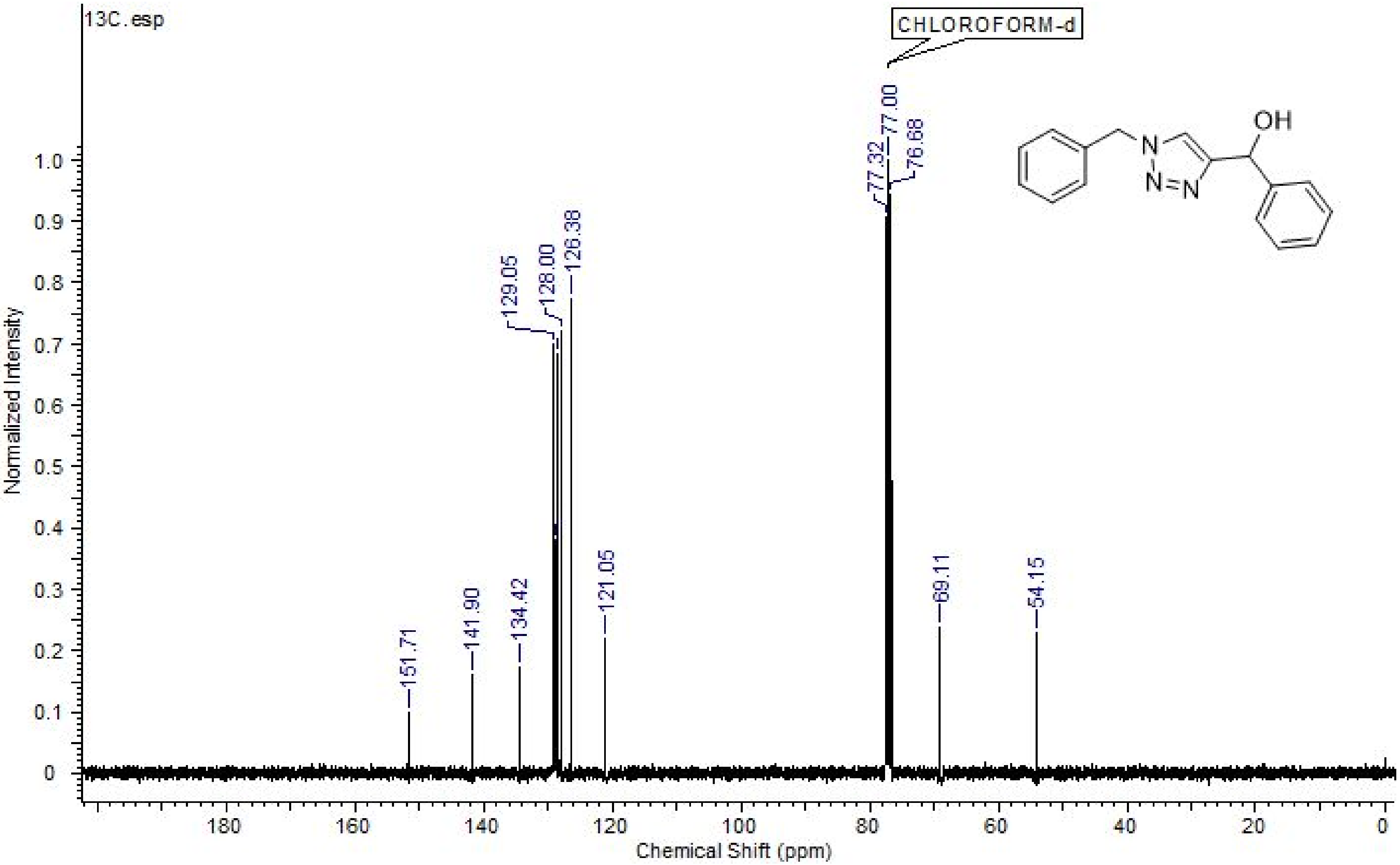

Figure 9S. ^{13}C NMR spectra (75 MHz, $CDCl_3$) of 4.

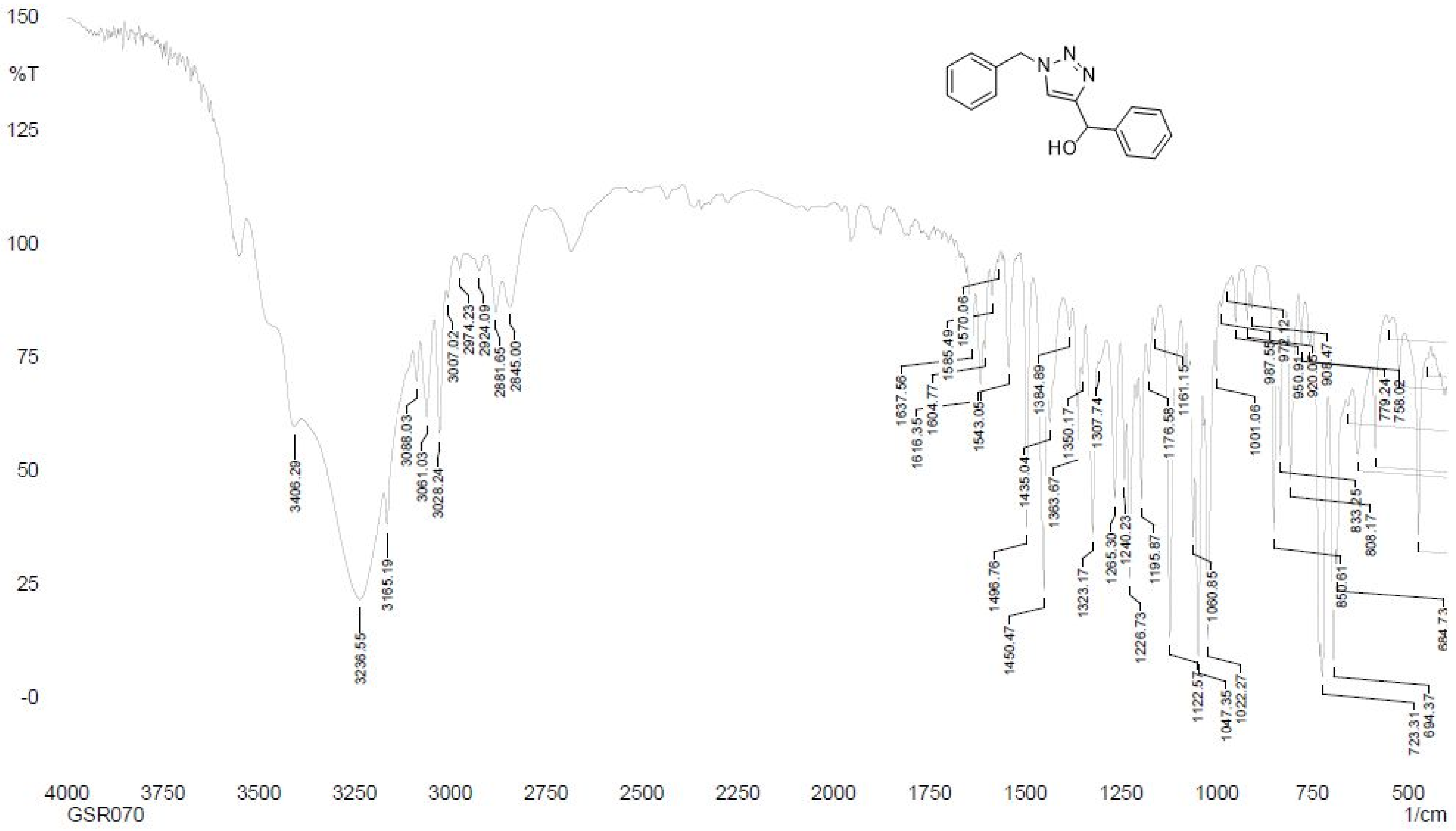

Figure 10S. FT-IR spectra (KBr disk) of 4.

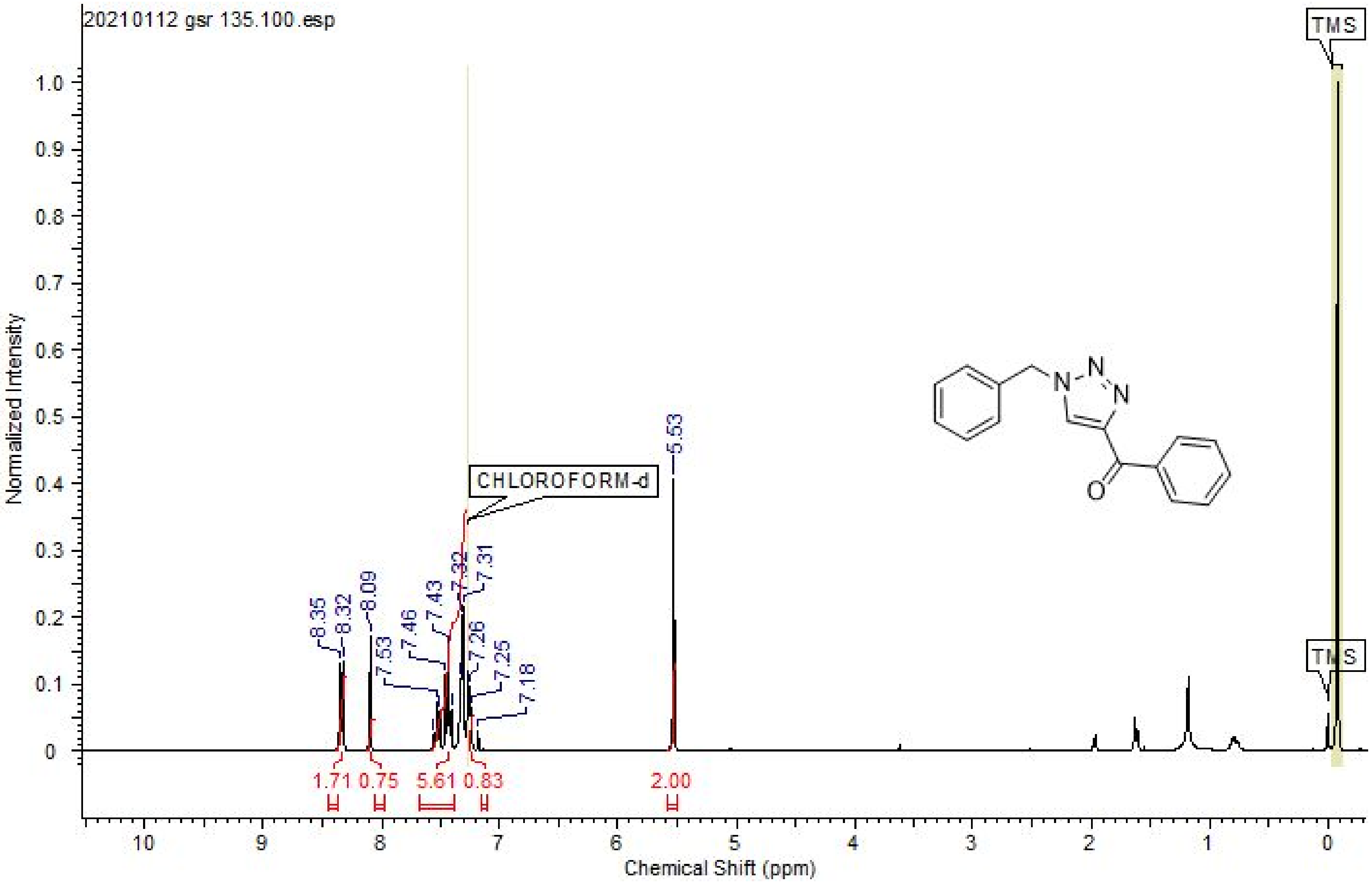

Figure 11S. ^{1}H NMR spectra (300 MHz, $CDCl_3$) of 5

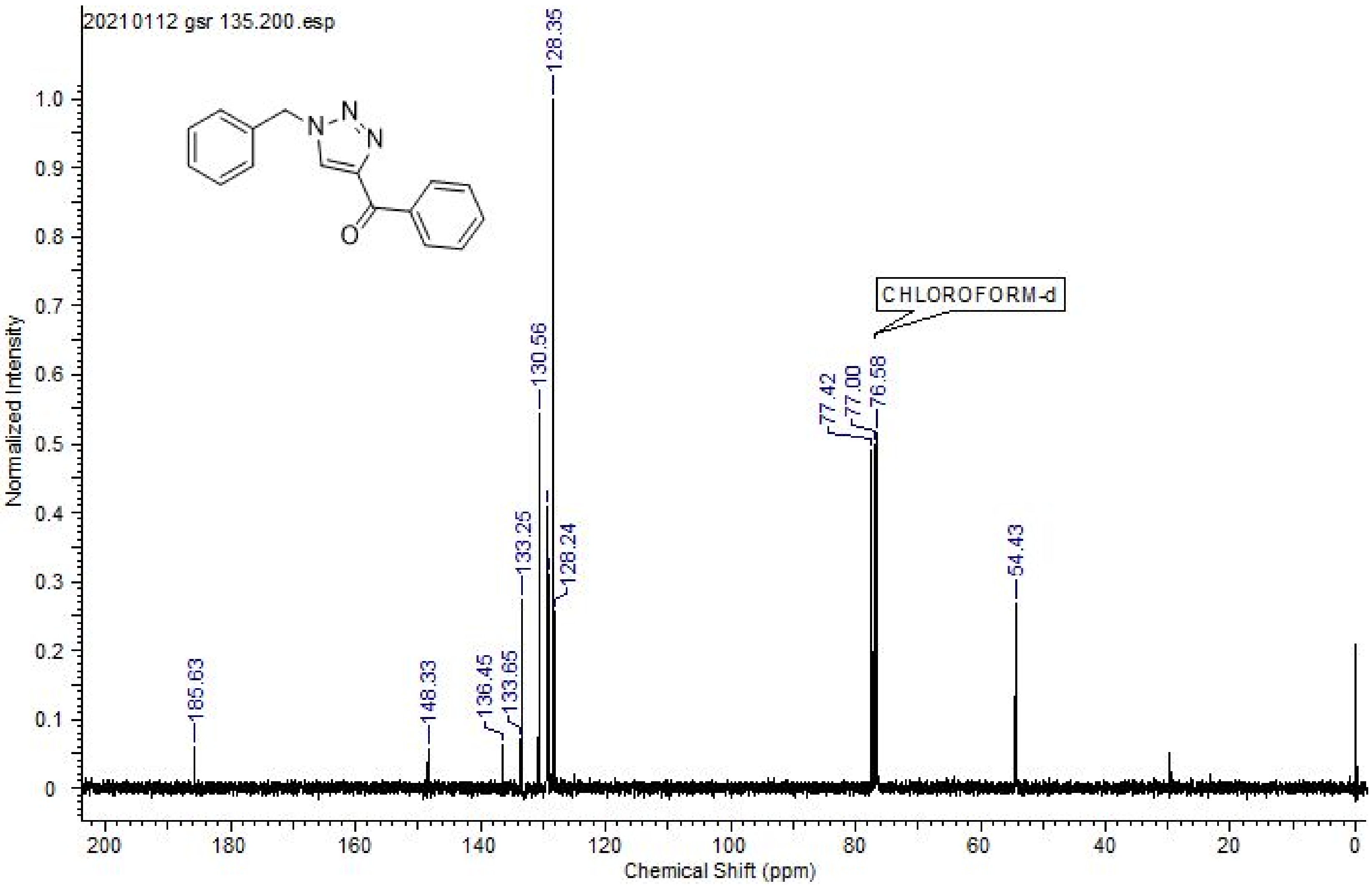

Figure 12S. ^{13}C NMR spectra (75 MHz, $CDCl_3$) of 5.

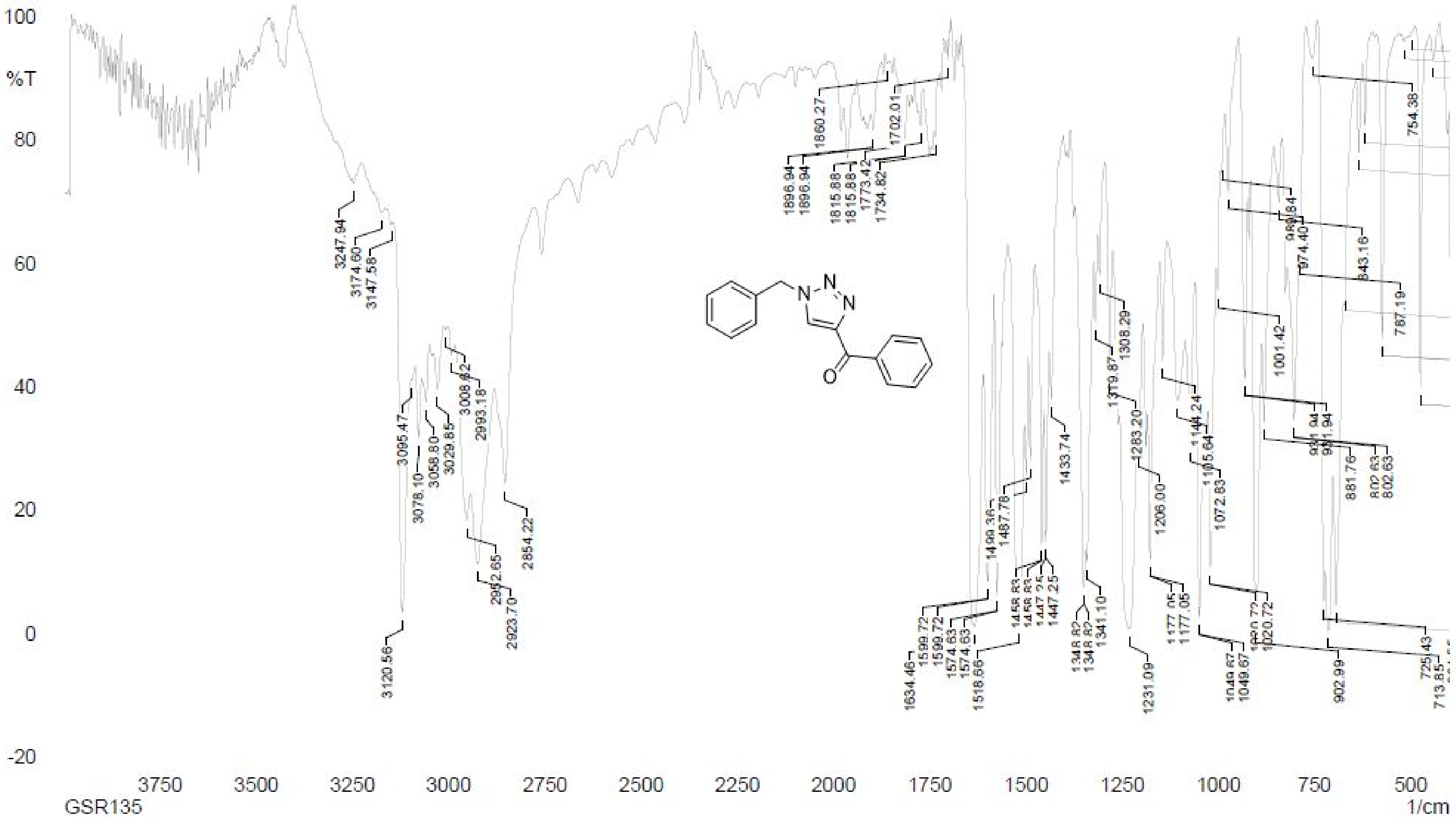

Figure 13S. FT-IR spectra (KBr disk) of 5.

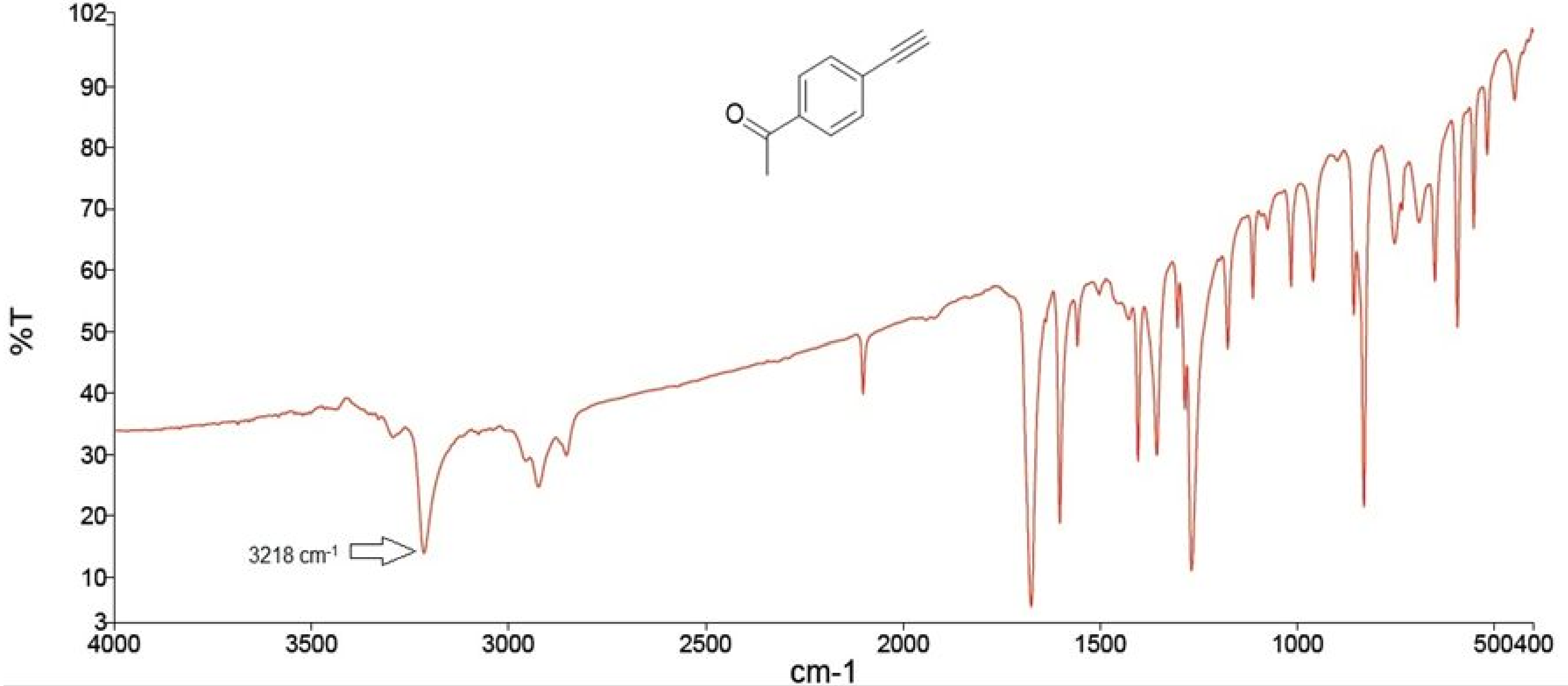

Figure 14S. FT-IR spectra (KBr disk) of 6a.

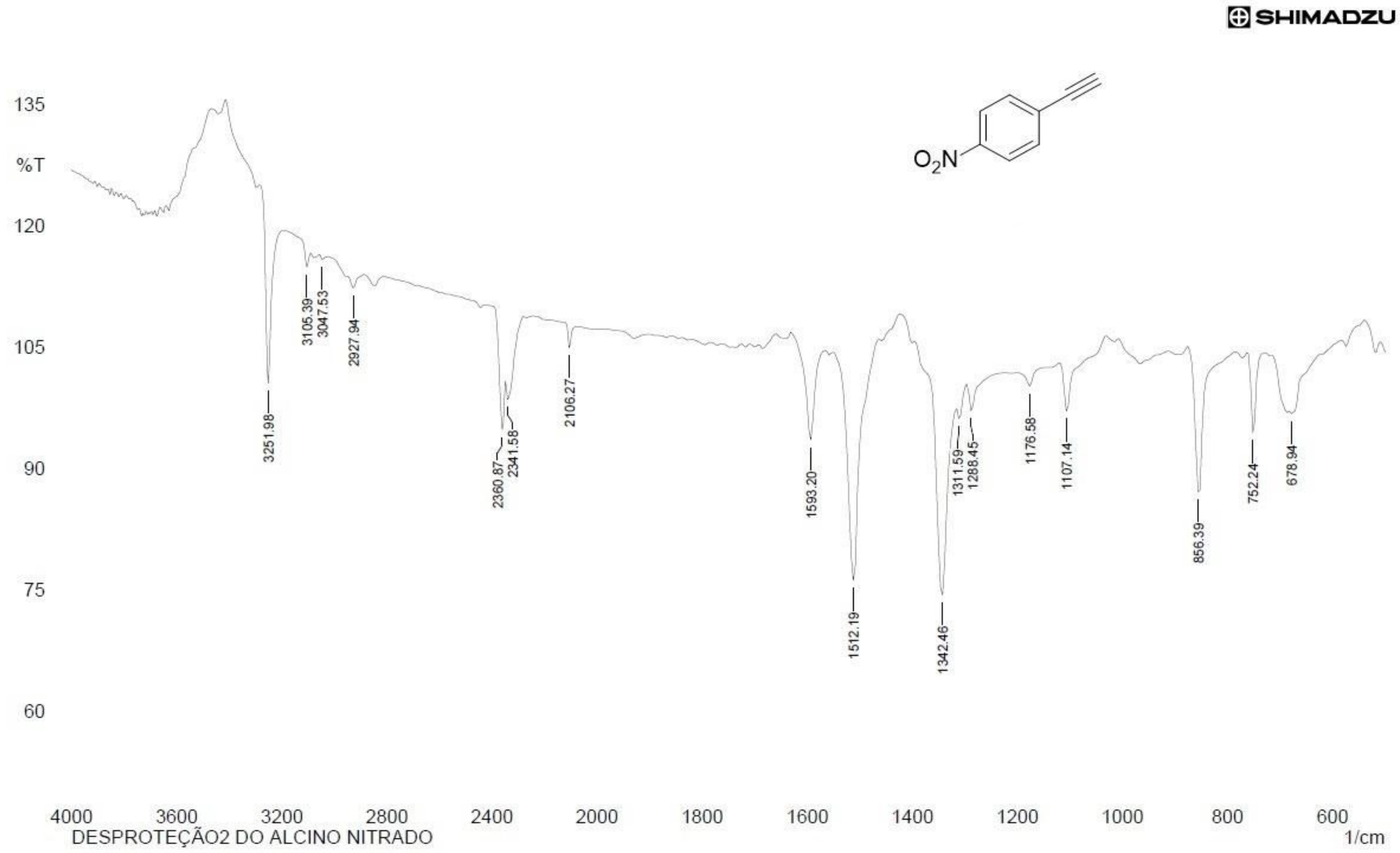

Figure 15S. FT-IR spectra (KBr disk) of 6b.

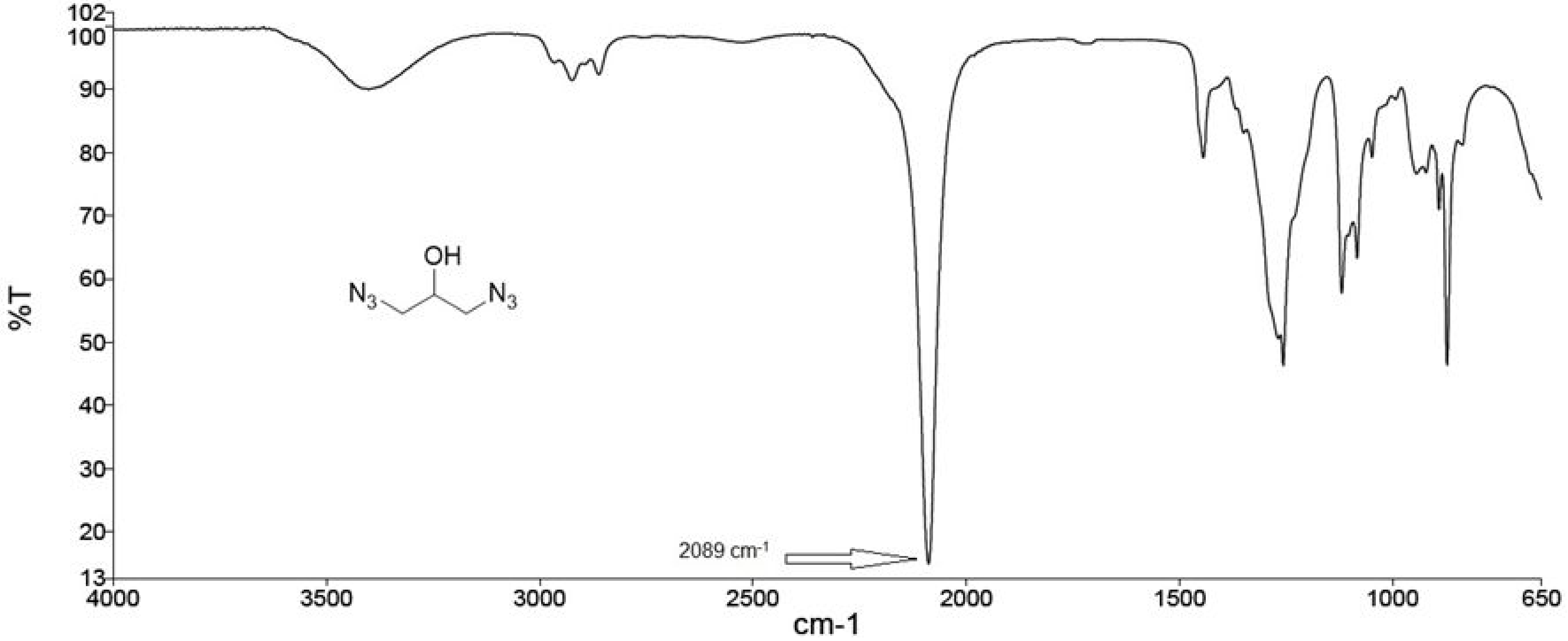

Figure 16S. FT-IR spectra (KBr disk) of 7.

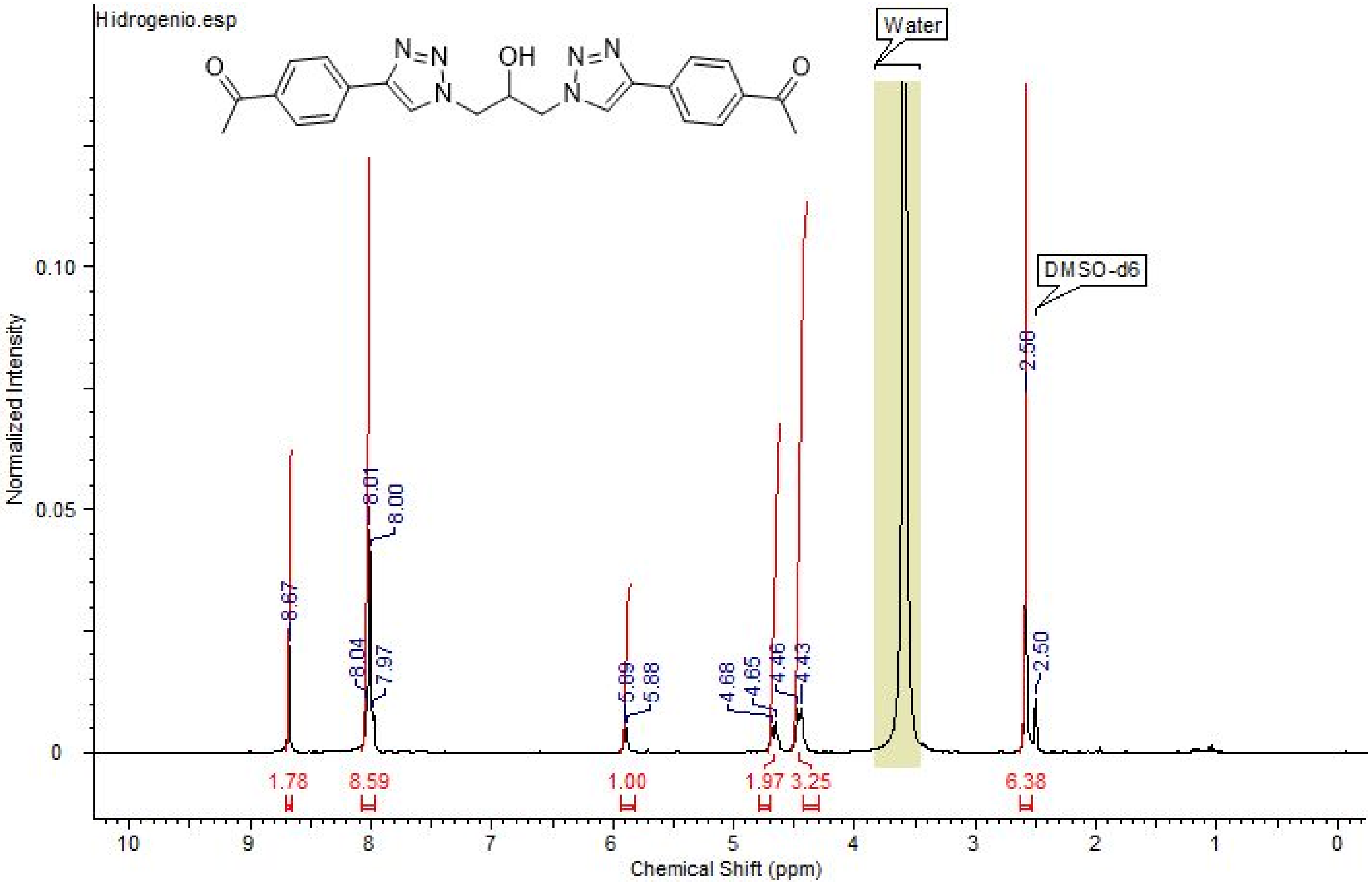

Figure 17S. ^{1}H NMR spectra (300 MHz, $CDCl_3$) of 8a

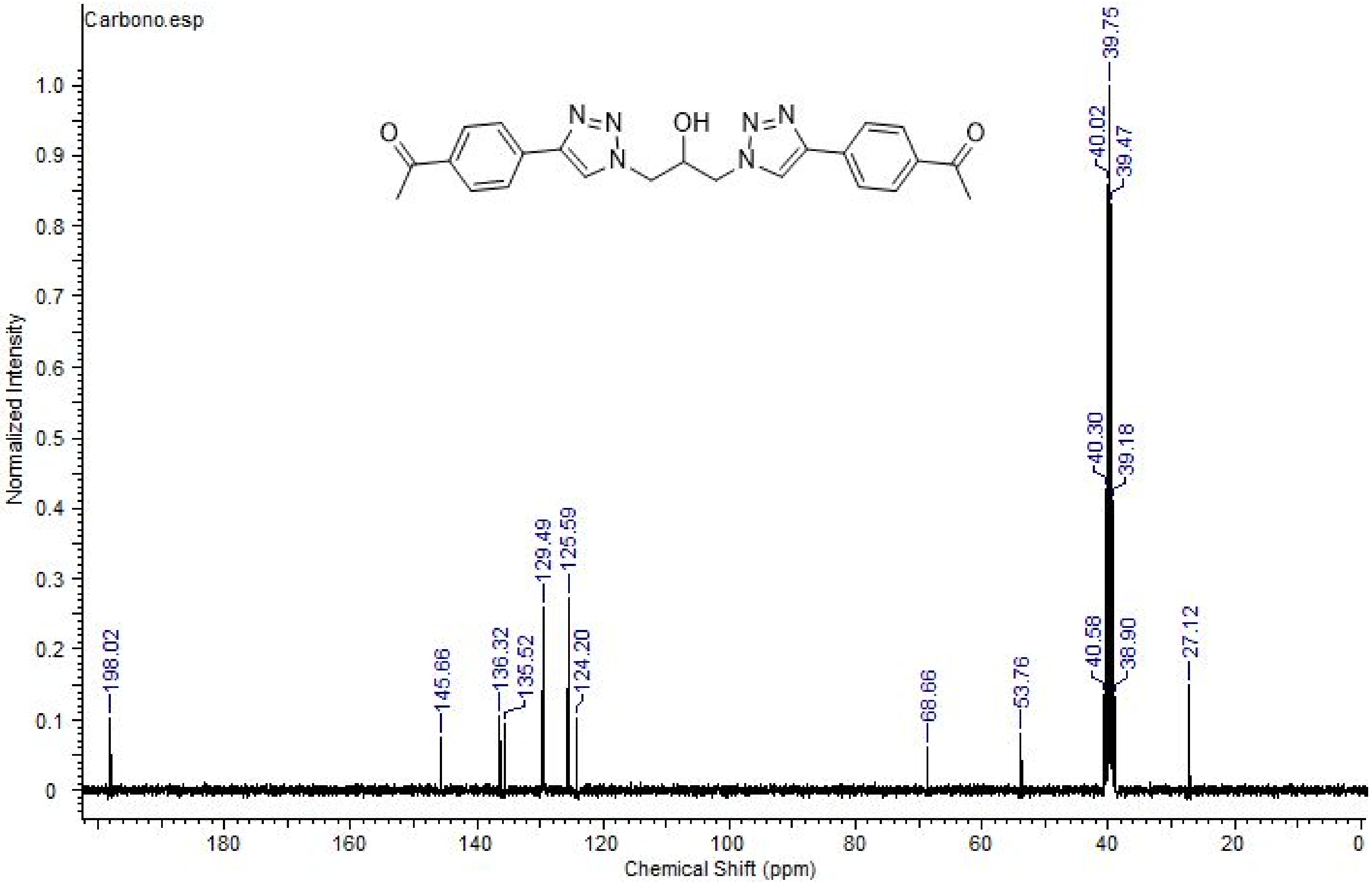

Figure 18S. ^{13}C NMR spectra (75 MHz, $CDCl_3$) of 8a.

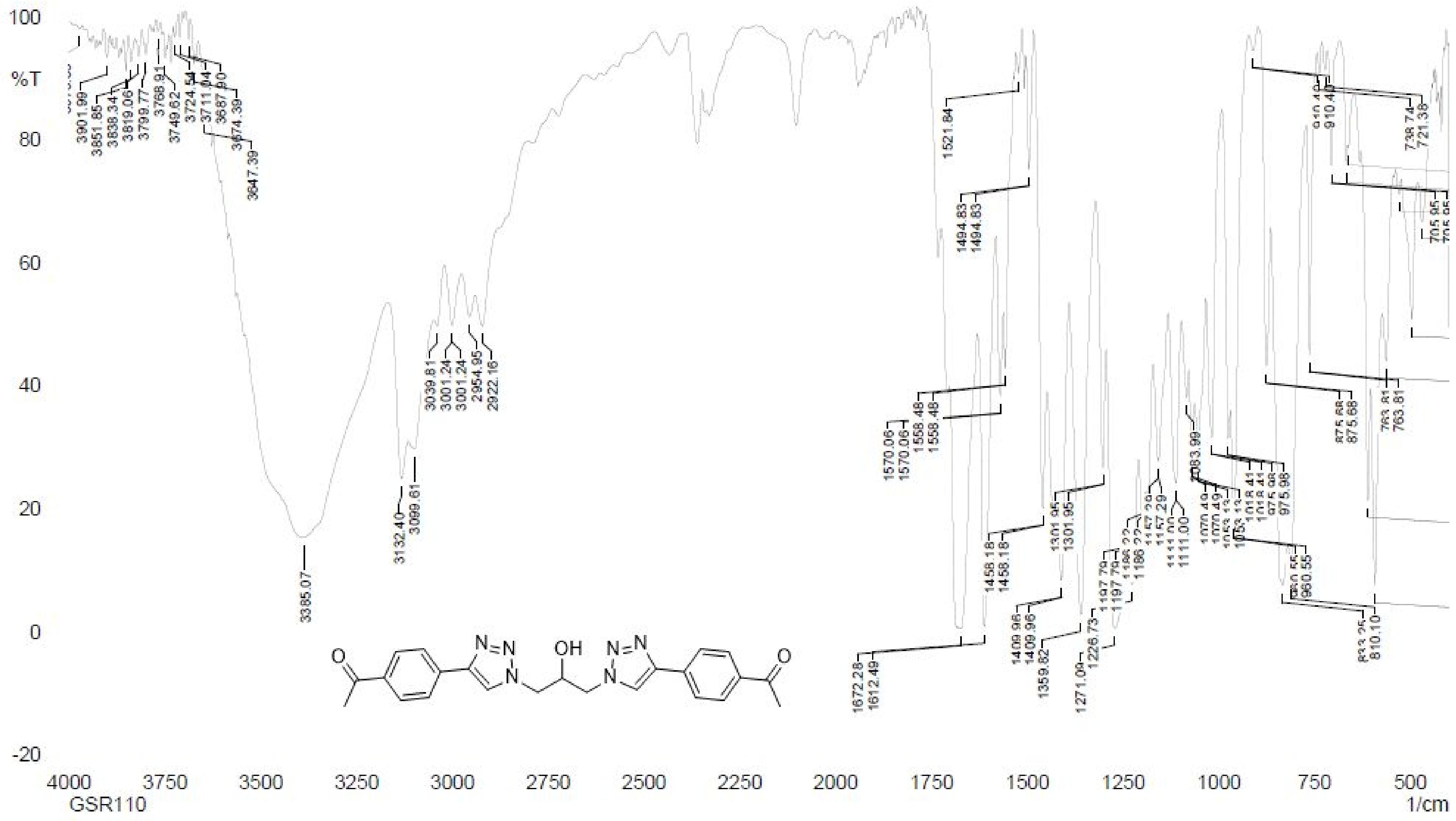

Figure 19S. FT-IR spectra (KBr disk) of 8a.

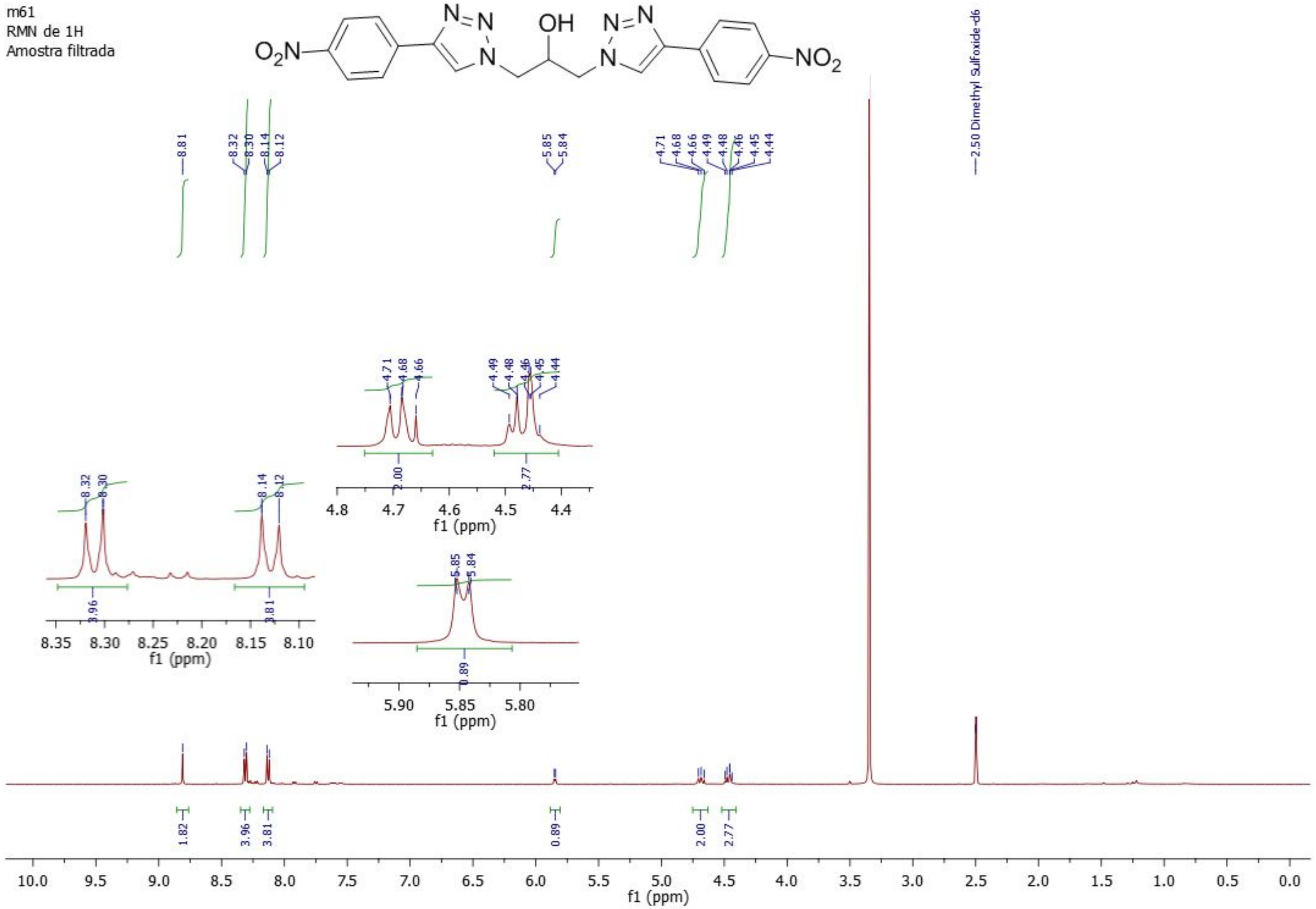

Figure 20S. ^{1}H NMR spectra (500 MHz, DMSO-d_6) of 8b

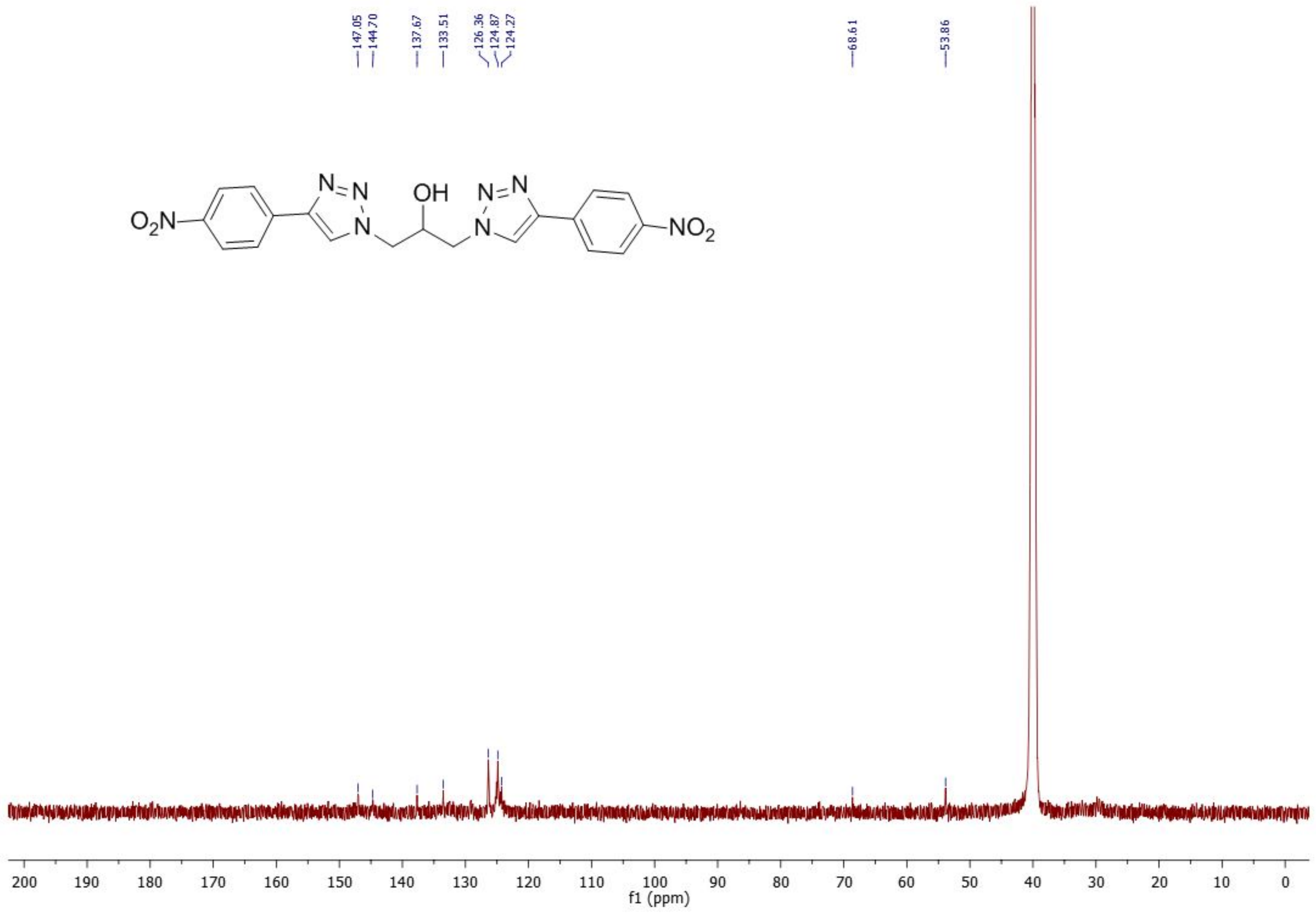

Figure 21S. ^{13}C NMR spectra (125 MHz, DMSO-d_6) of 8b.

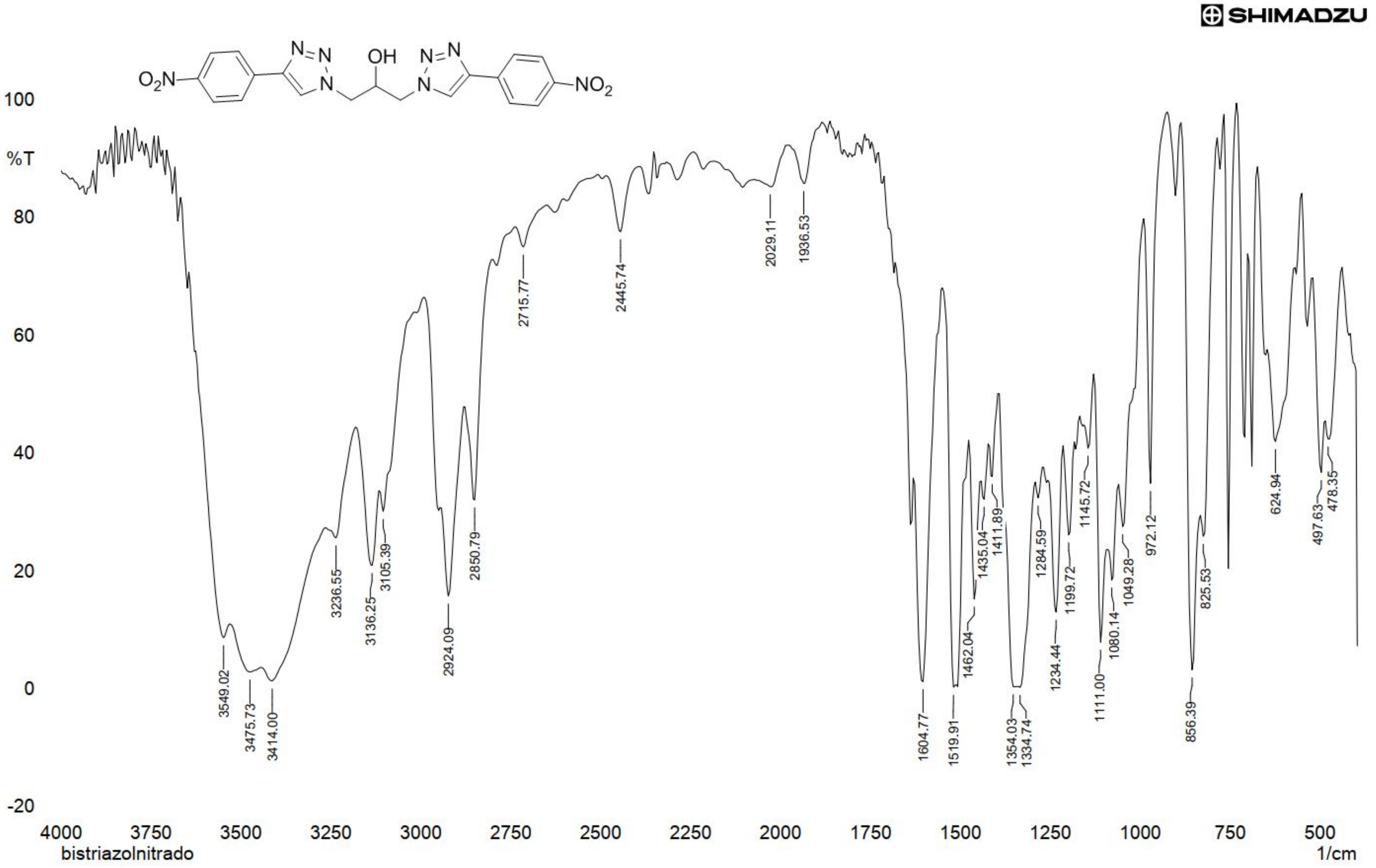

Figure 22S. FT-IR spectra (KBr disk) of 8b.

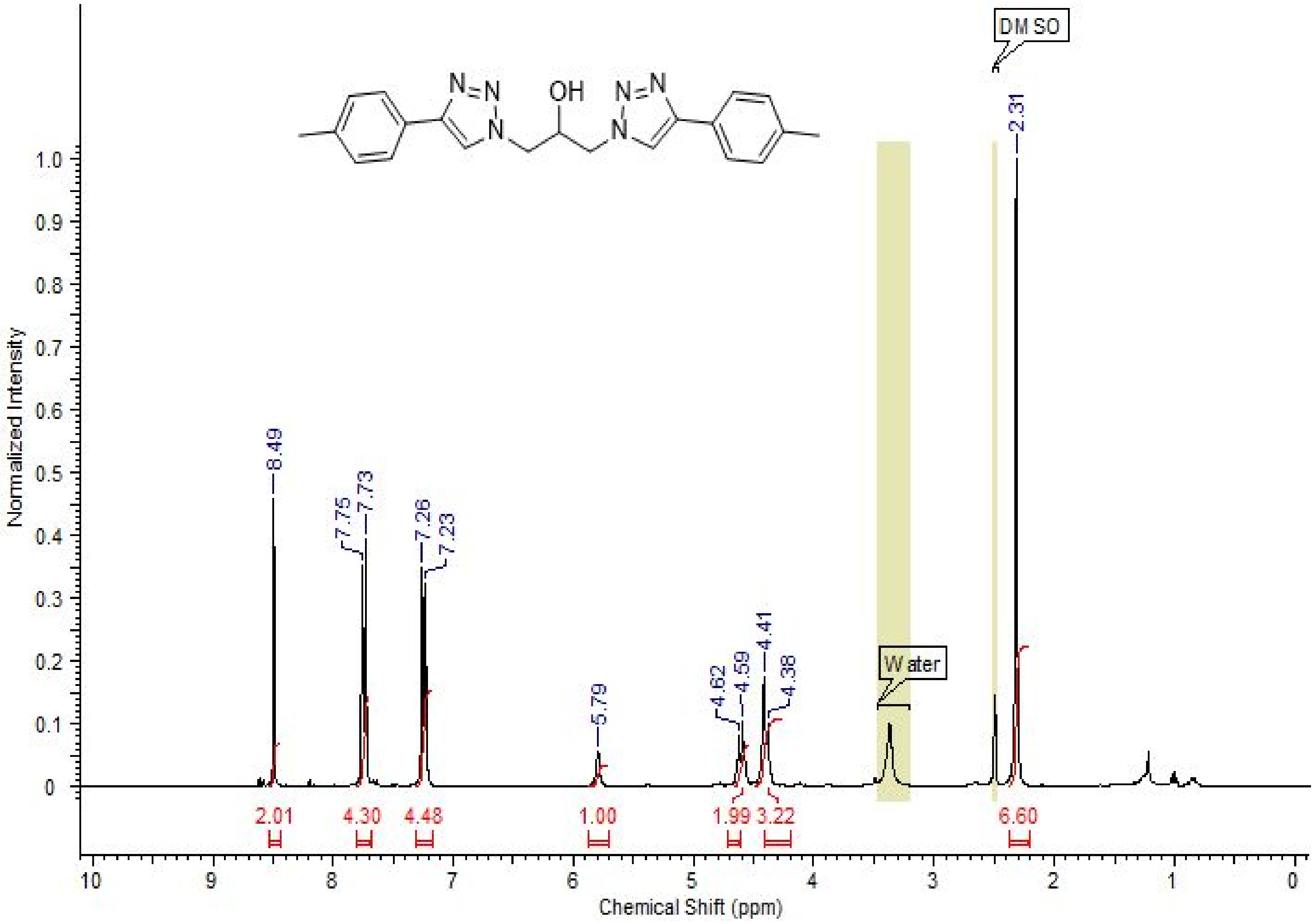

Figure 23S. ^{1}H NMR spectra (300 MHz, DMSO-d_6) of 8c

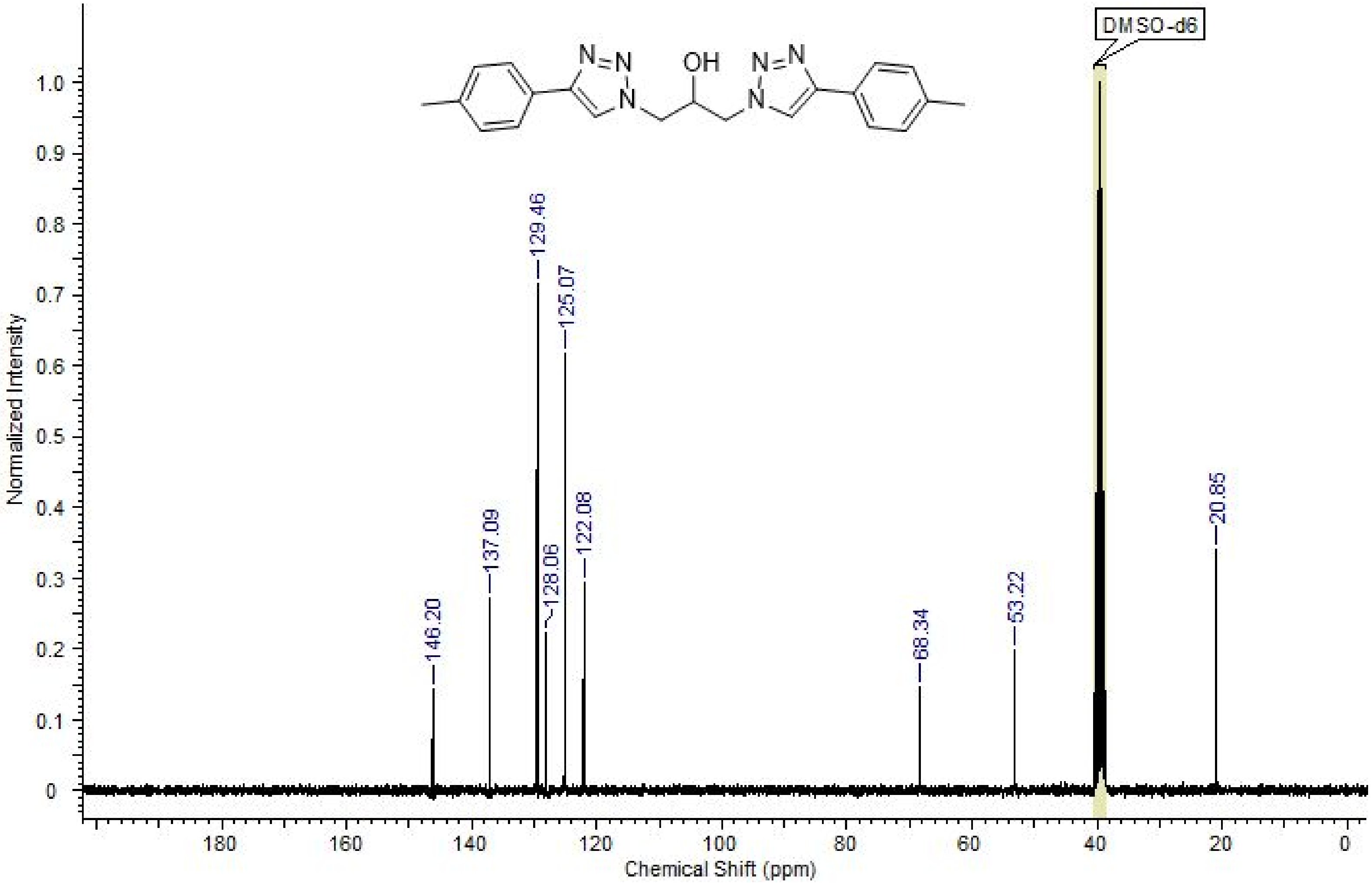

Figure 24S. ^{13}C NMR spectra (75 MHz, DMSO-d_6) of 8c

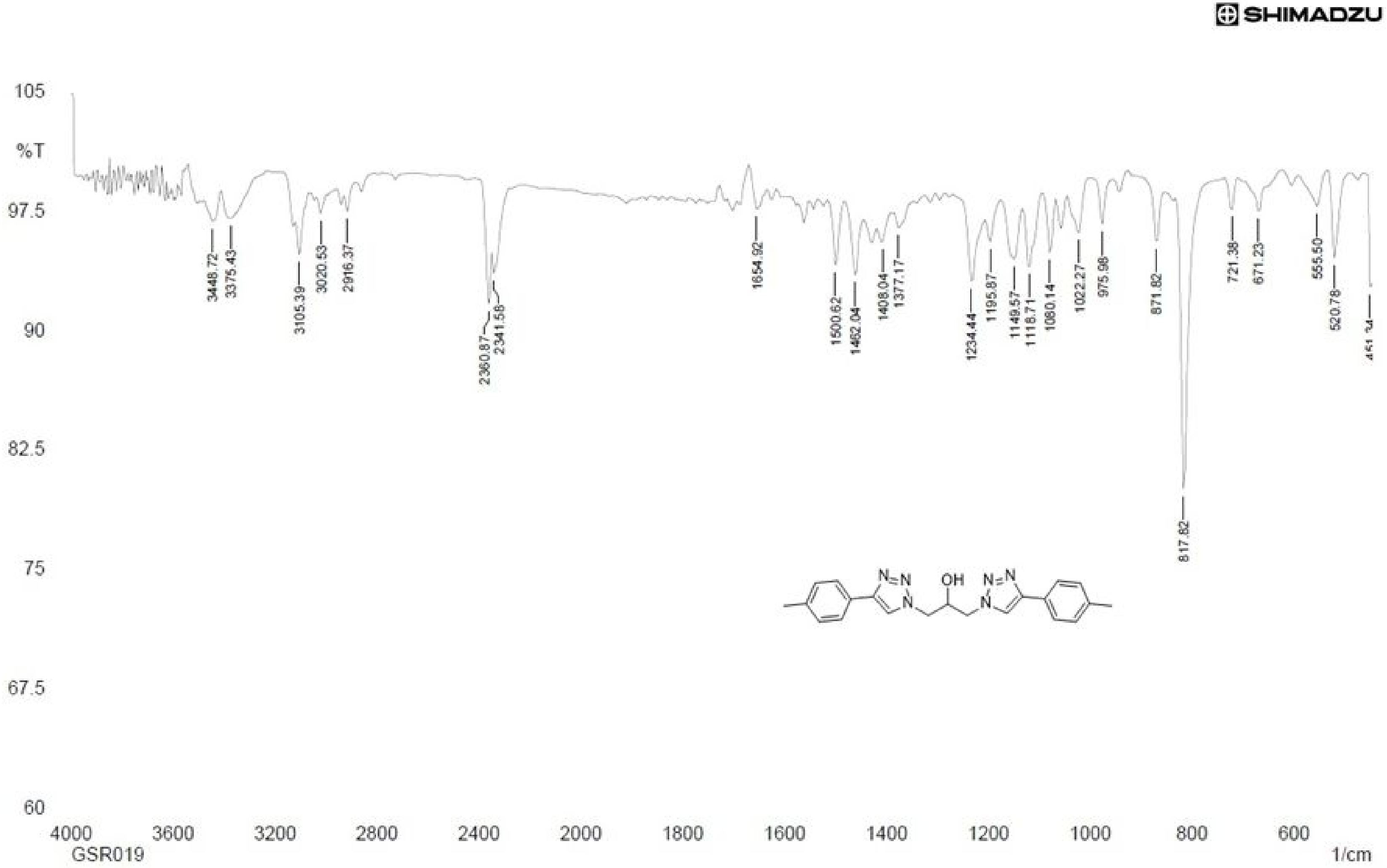

Figure 25S. FT-IR spectra (KBr disk) of 8c.

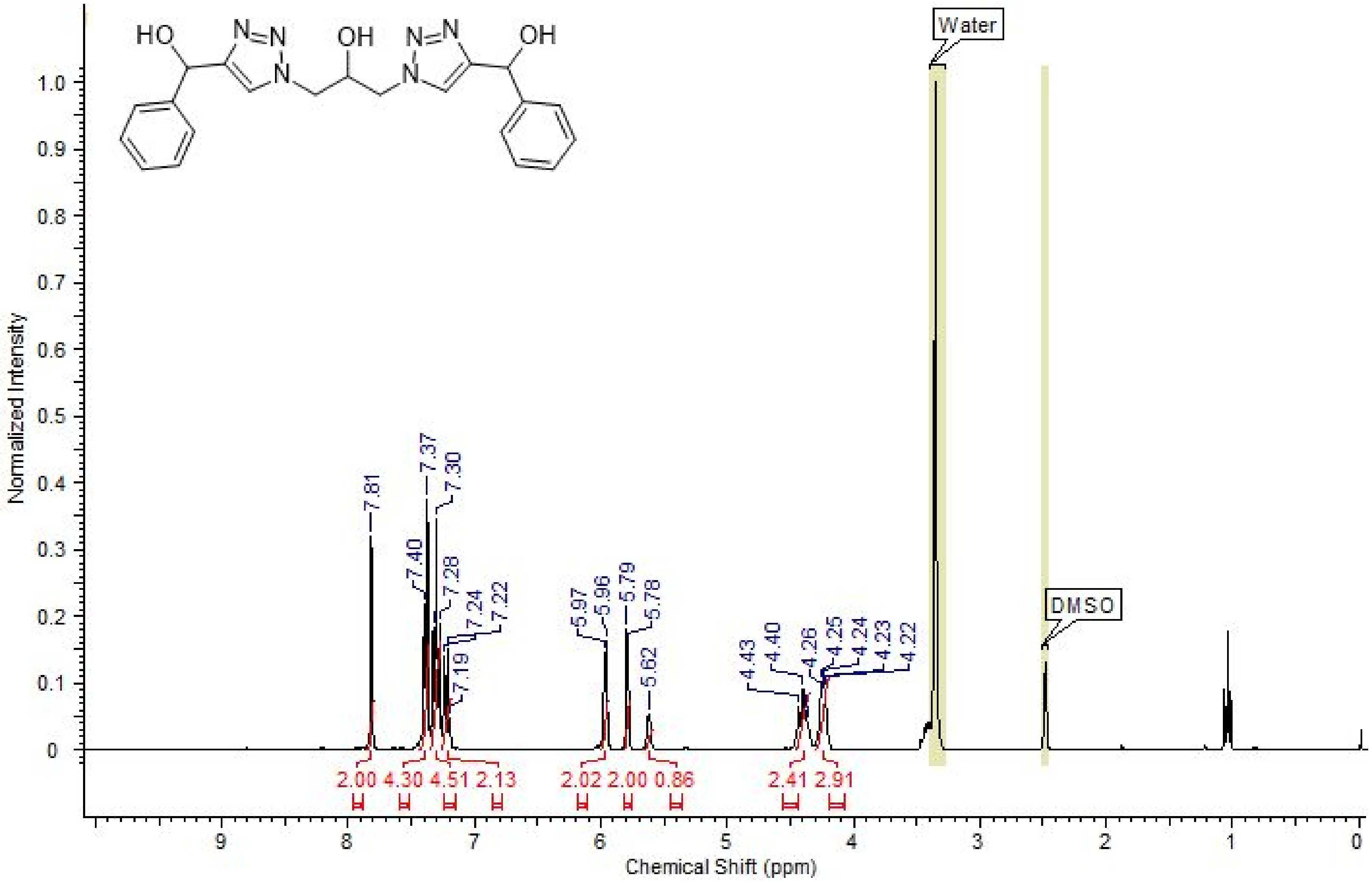

Figure 26S. ^{1}H NMR spectra (300 MHz, DMSO-d_6) of 9**.**

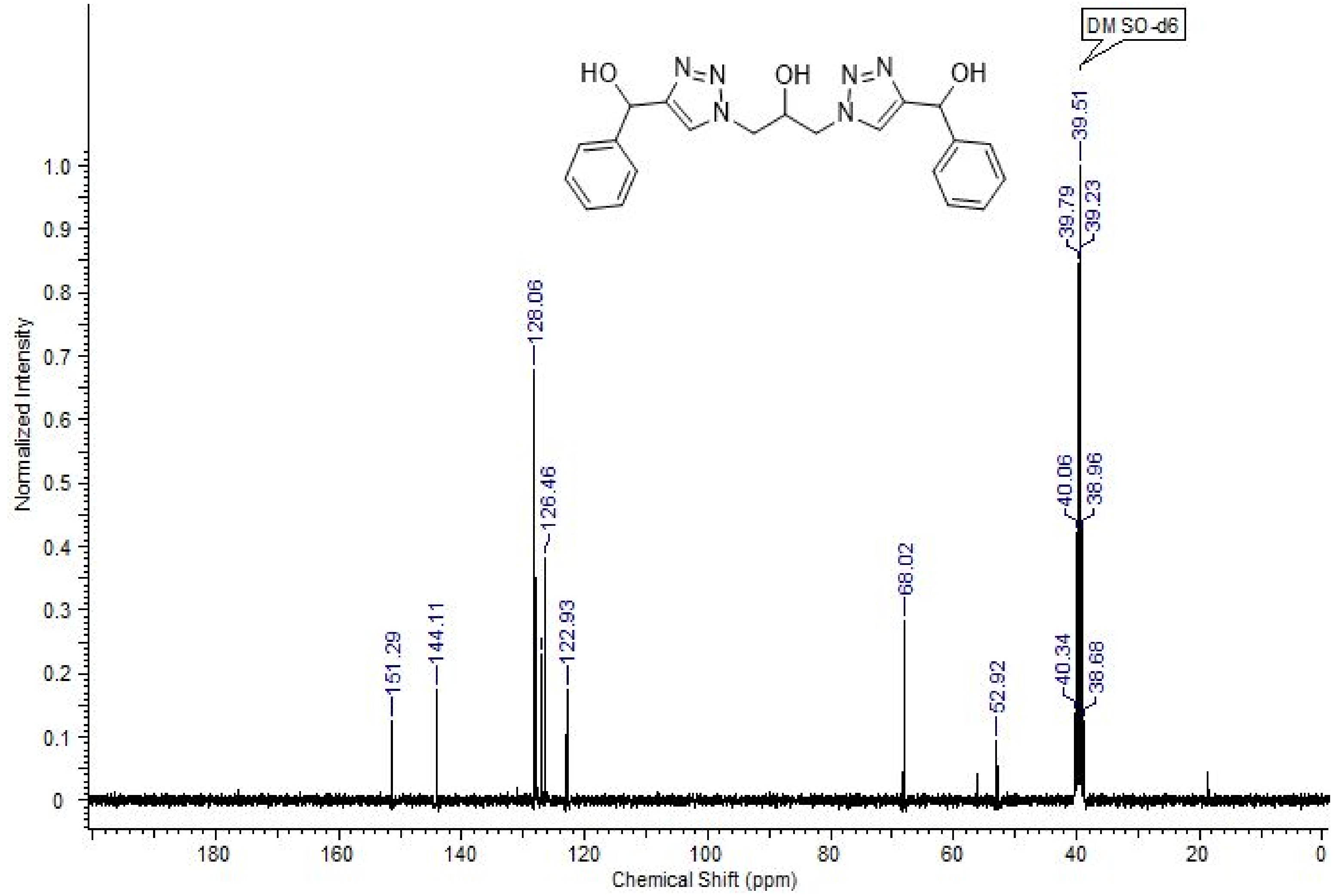

Figure 27S. ^{13}C NMR spectra (75 MHz, DMSO-d_6) of 9**.**

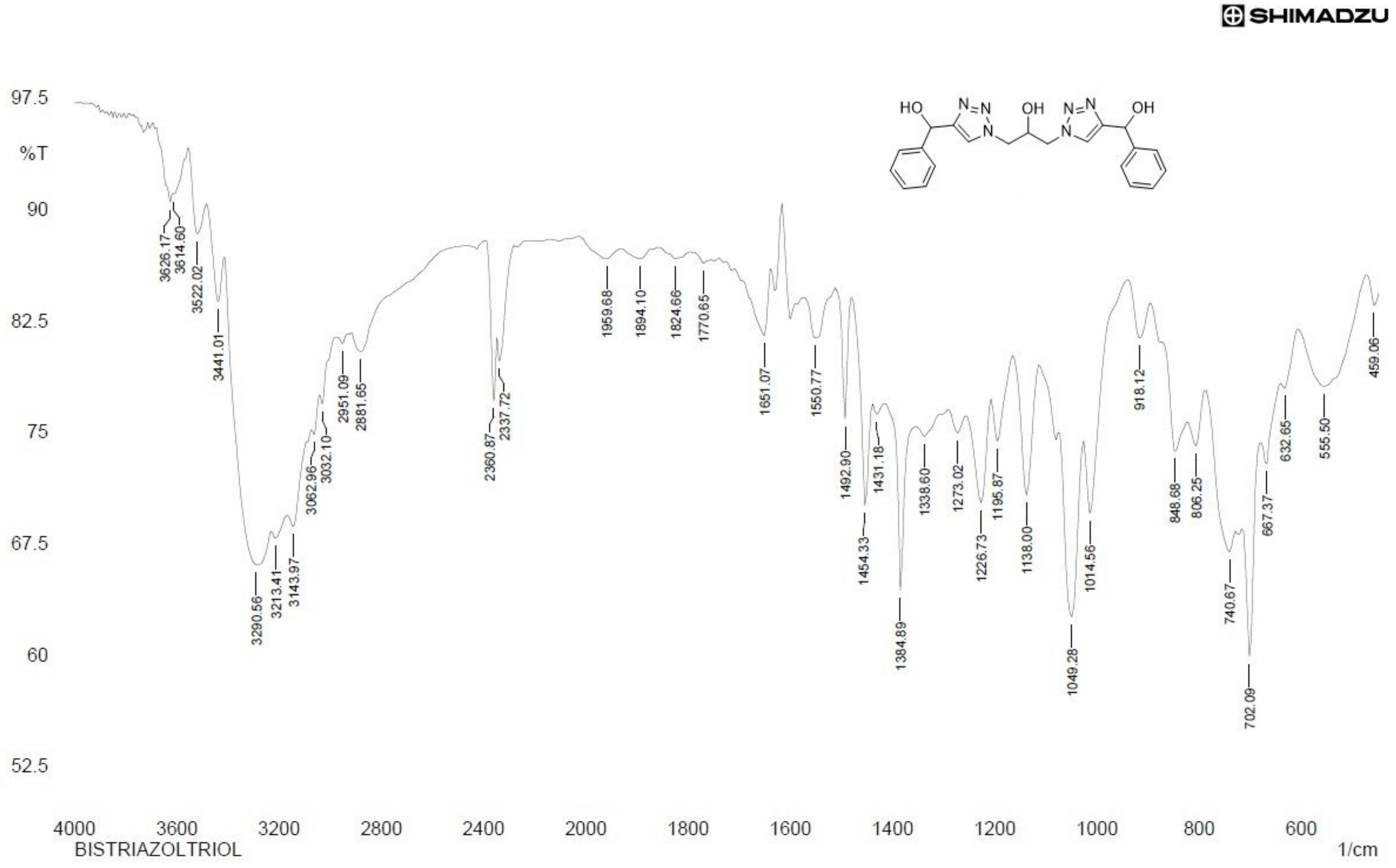

Figure 28S. FT-IR spectra (KBr disk) of 9.

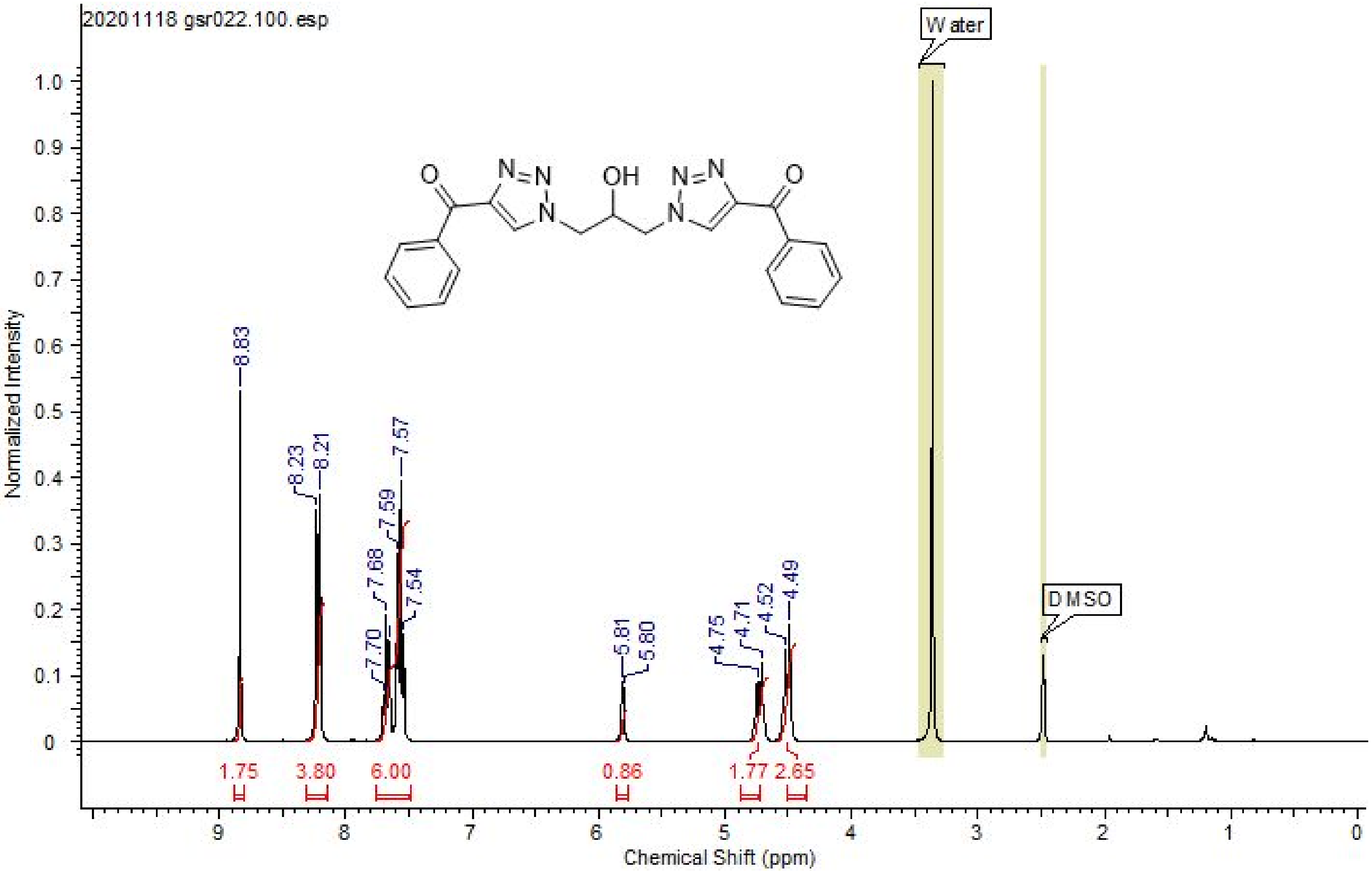

Figure 29S. ^{1}H NMR spectra (300 MHz, DMSO-d_6) of 10.

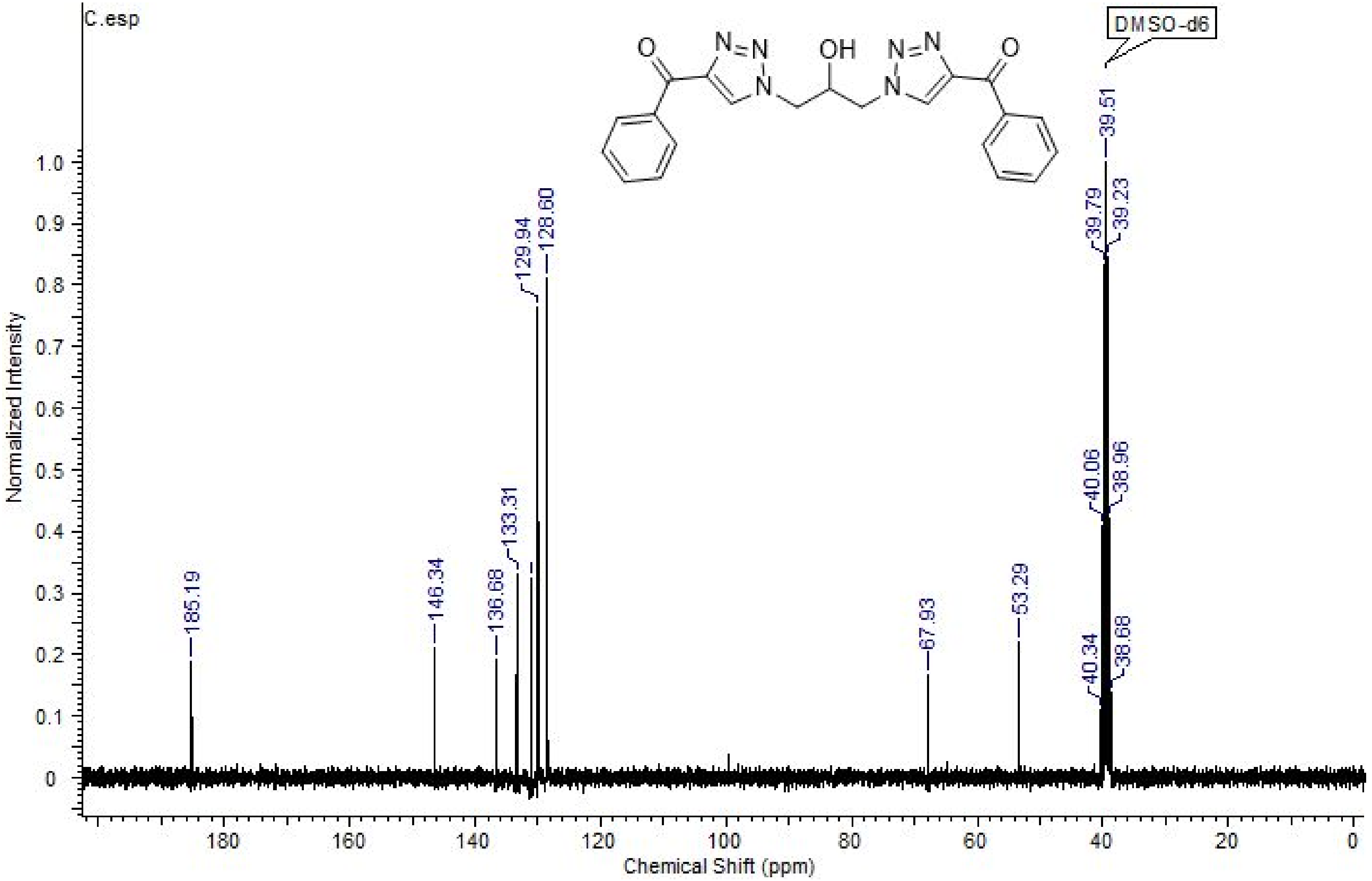

Figure 30S. ^{13}C NMR spectra (75 MHz, DMSO-d_6) of 10.

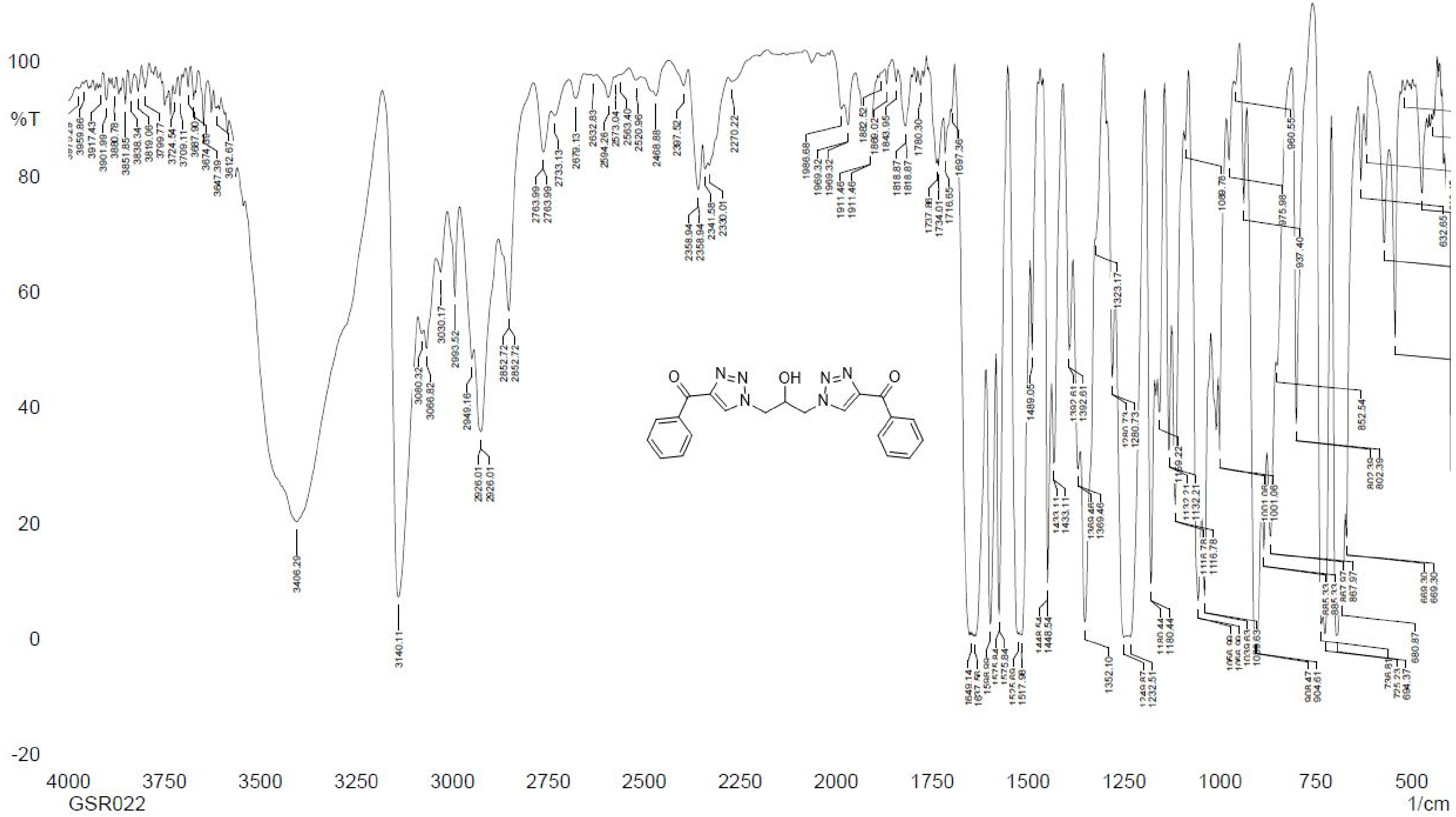

Figure 31S. FT-IR spectra (KBr disk) of 10.

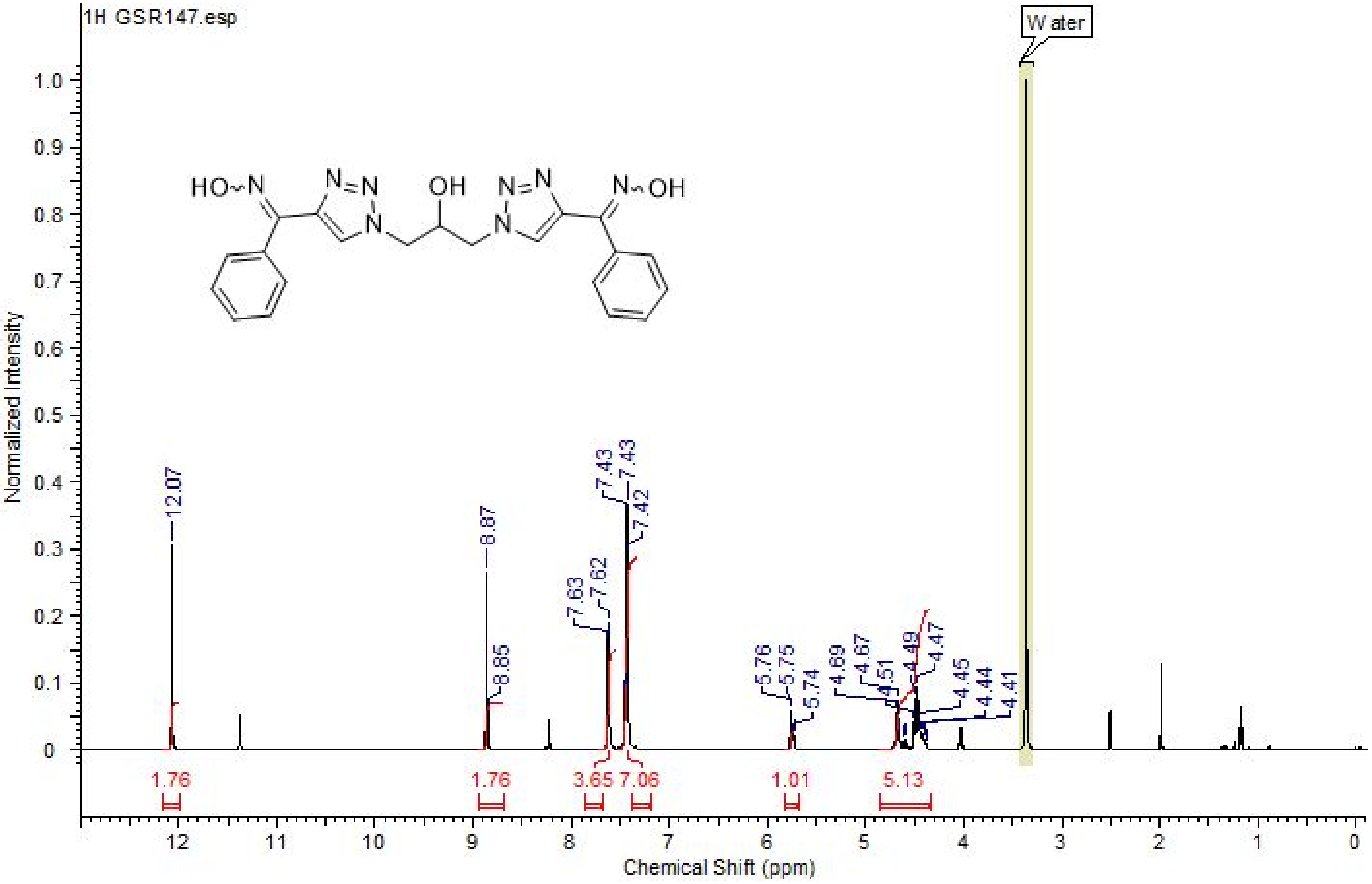

Figure 32S. ^{1}H NMR spectra (500 MHz, DMSO-d_6) of 11.

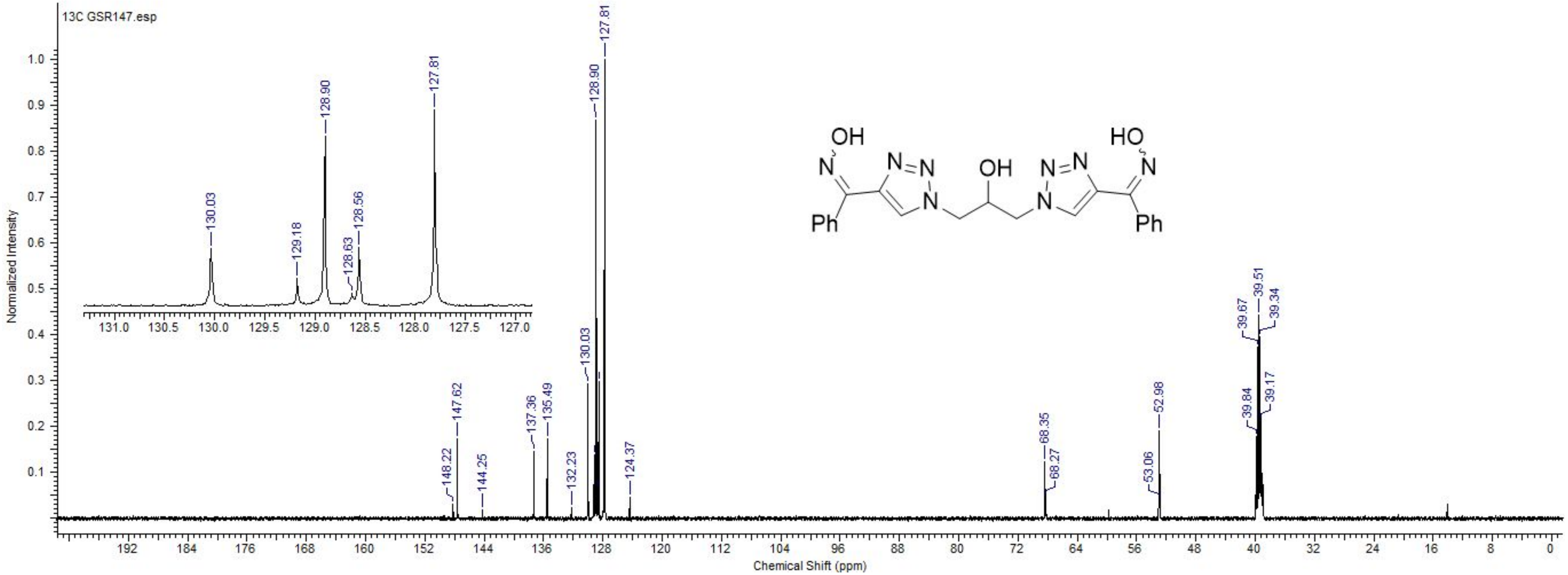

Figure 33S. ^{13}C NMR spectra (125 MHz, DMSO-d_6) of 11.

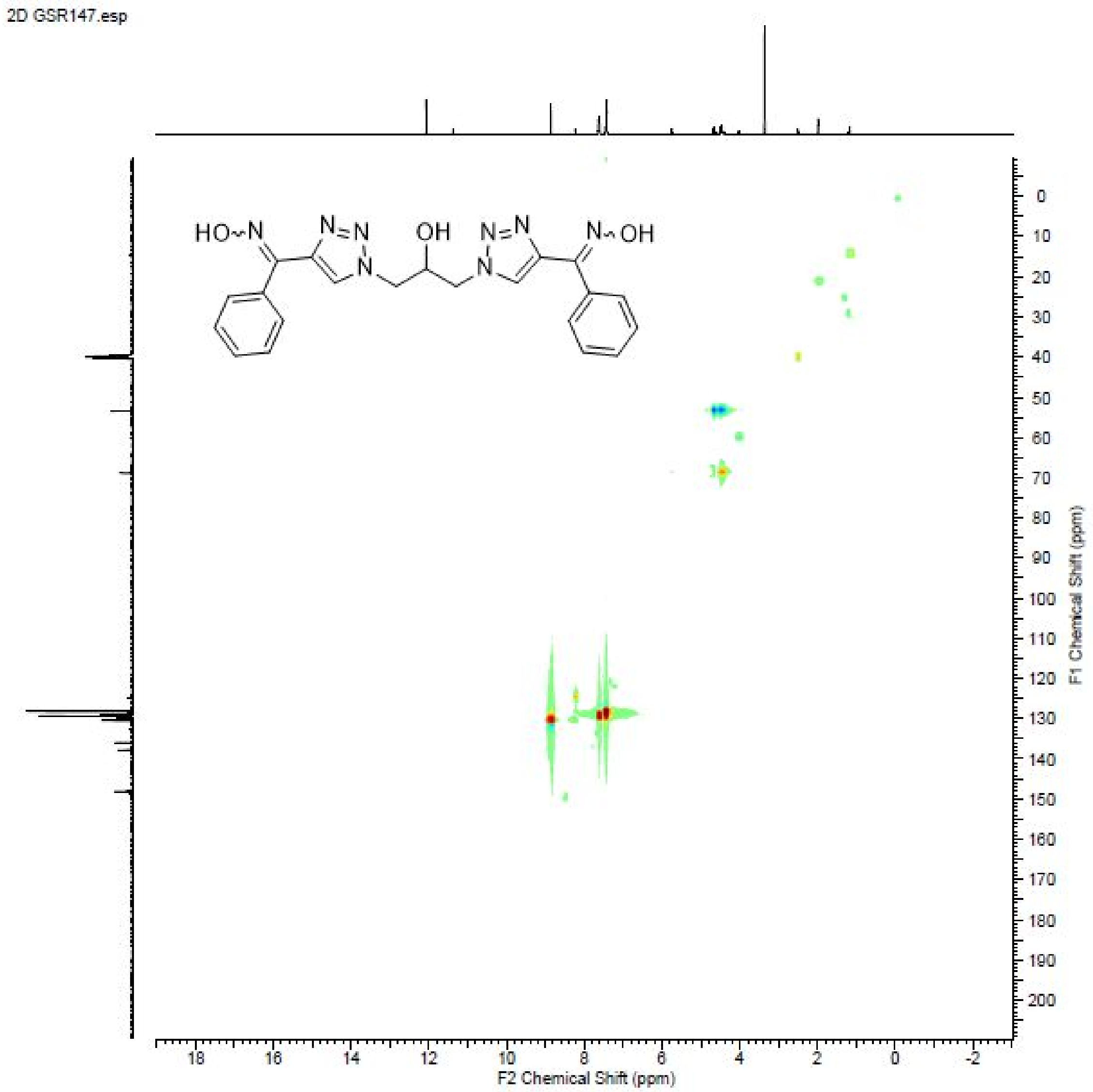

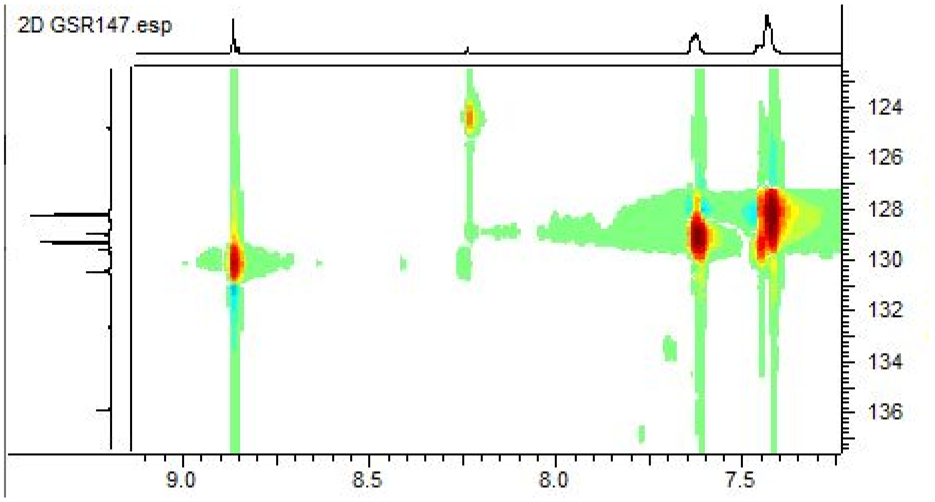

Figure 34S. HSQC spectra of 11.

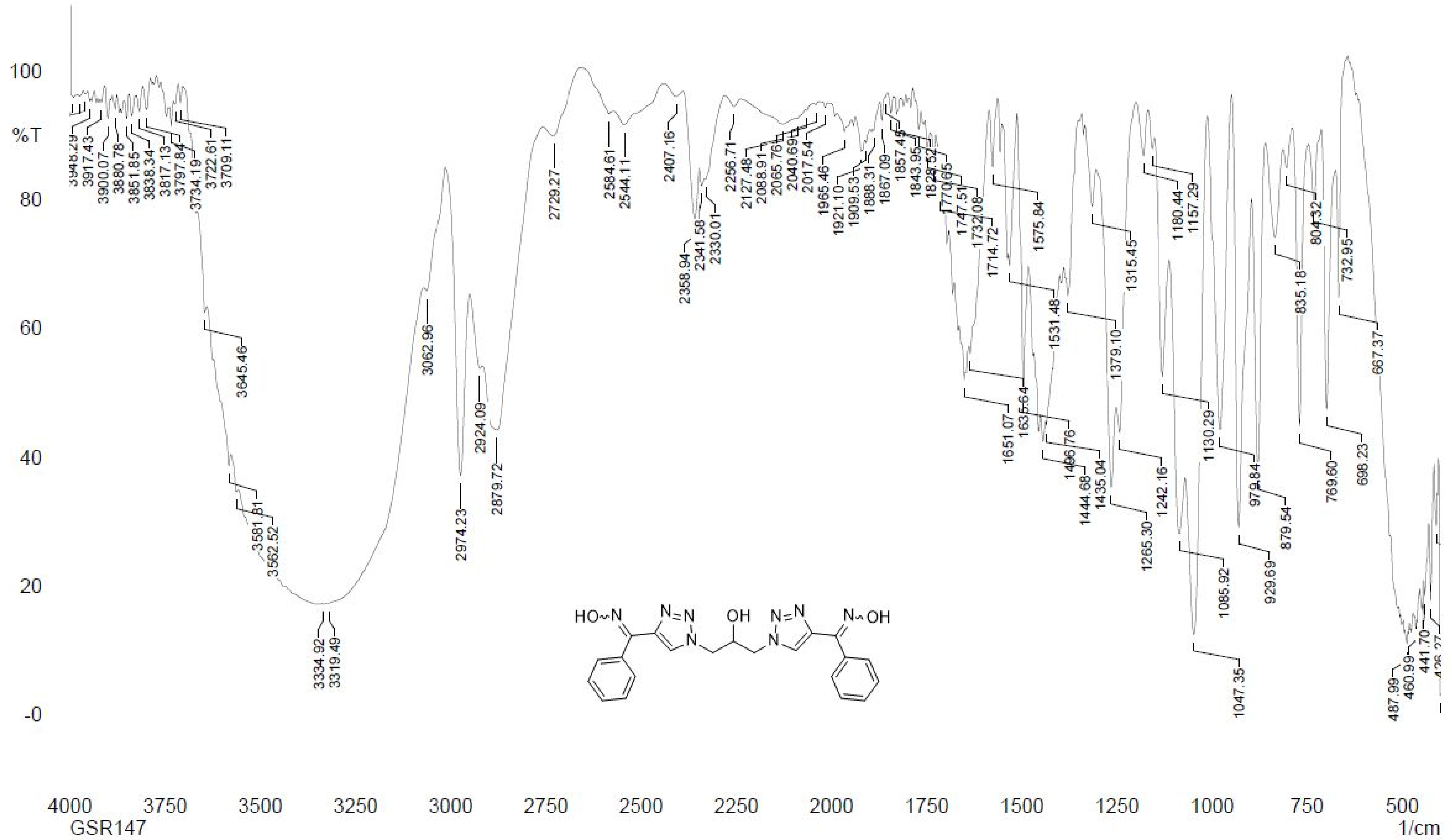

Figure 35S. FT-IR spectra (KBr disk) of 11.

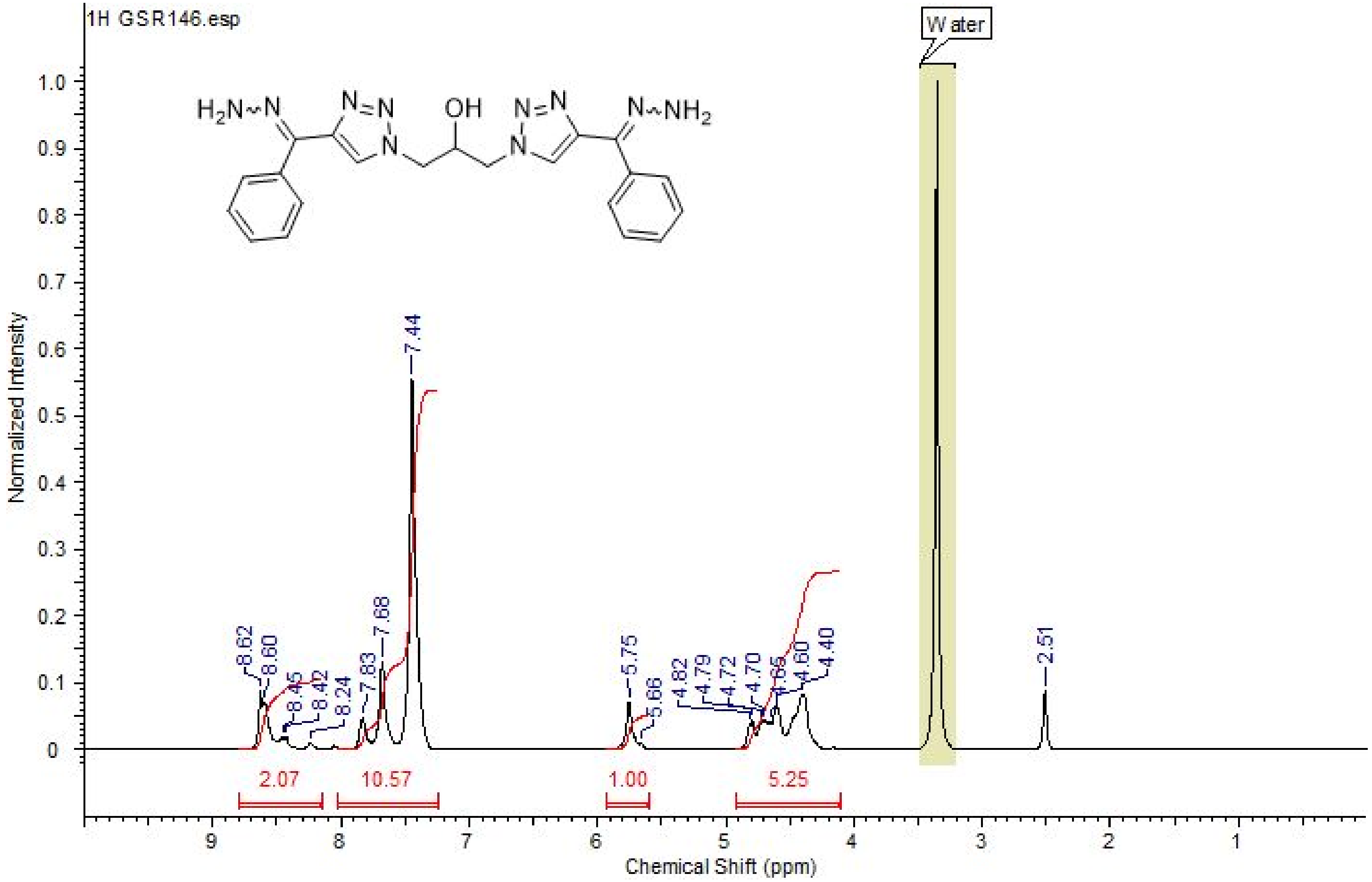

Figure 36S. ^{1}H NMR spectra (500 MHz, DMSO-d_6) of 12a**.**

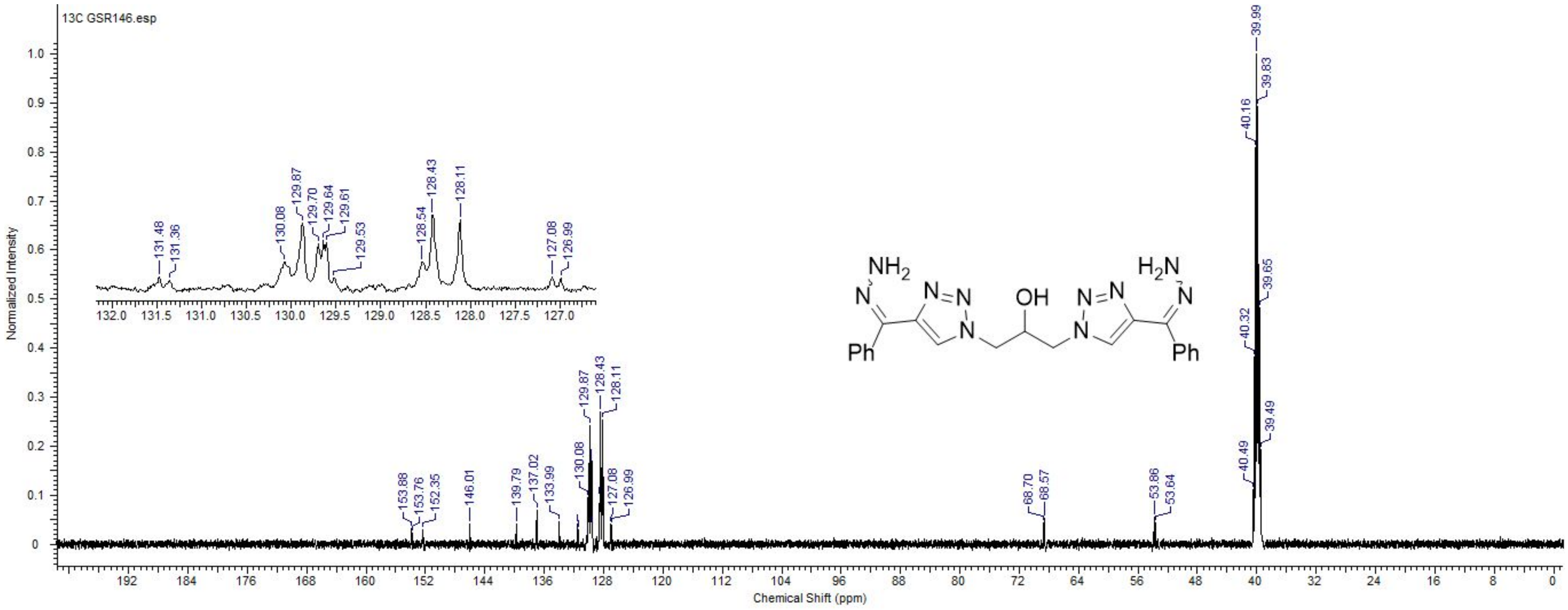

Figure 37S. ^{13}C NMR spectra (125 MHz, DMSO-d_6) of 12a.

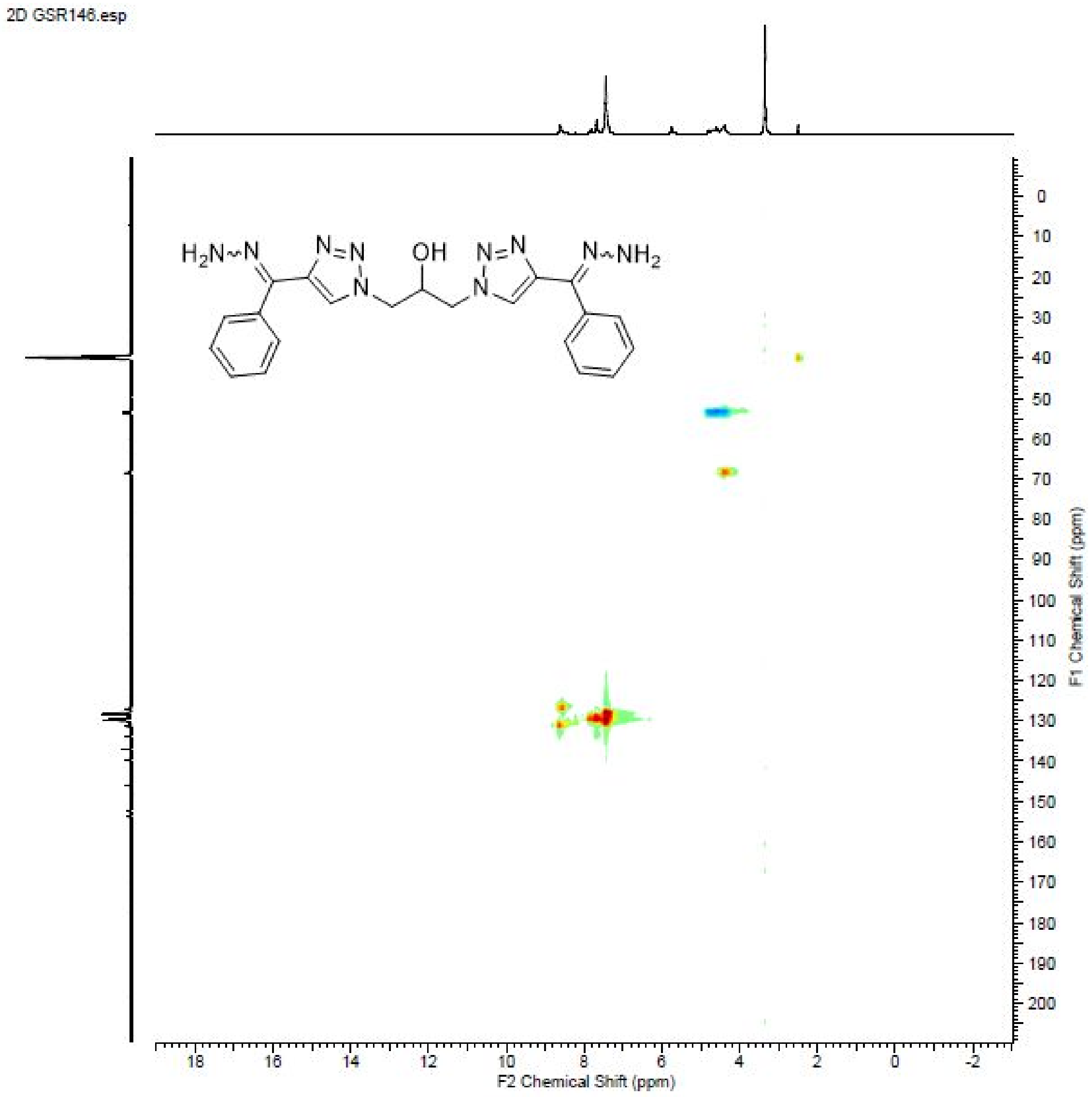

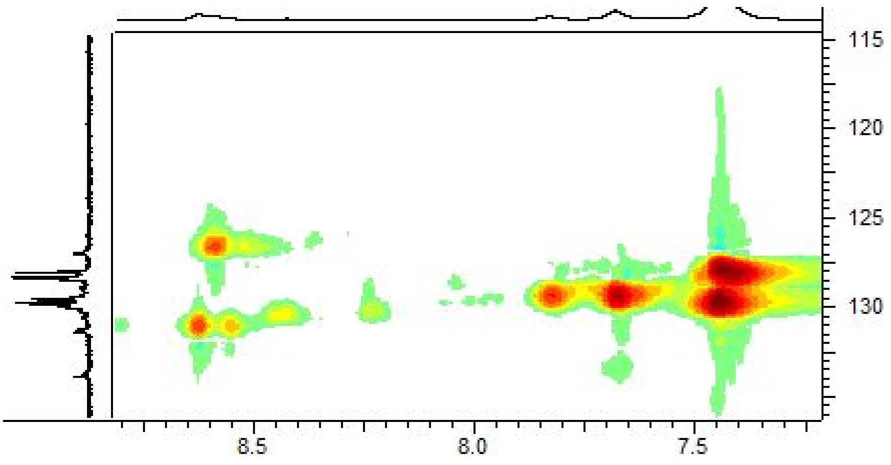

Figure 38S. HSQC spectra of 12a.

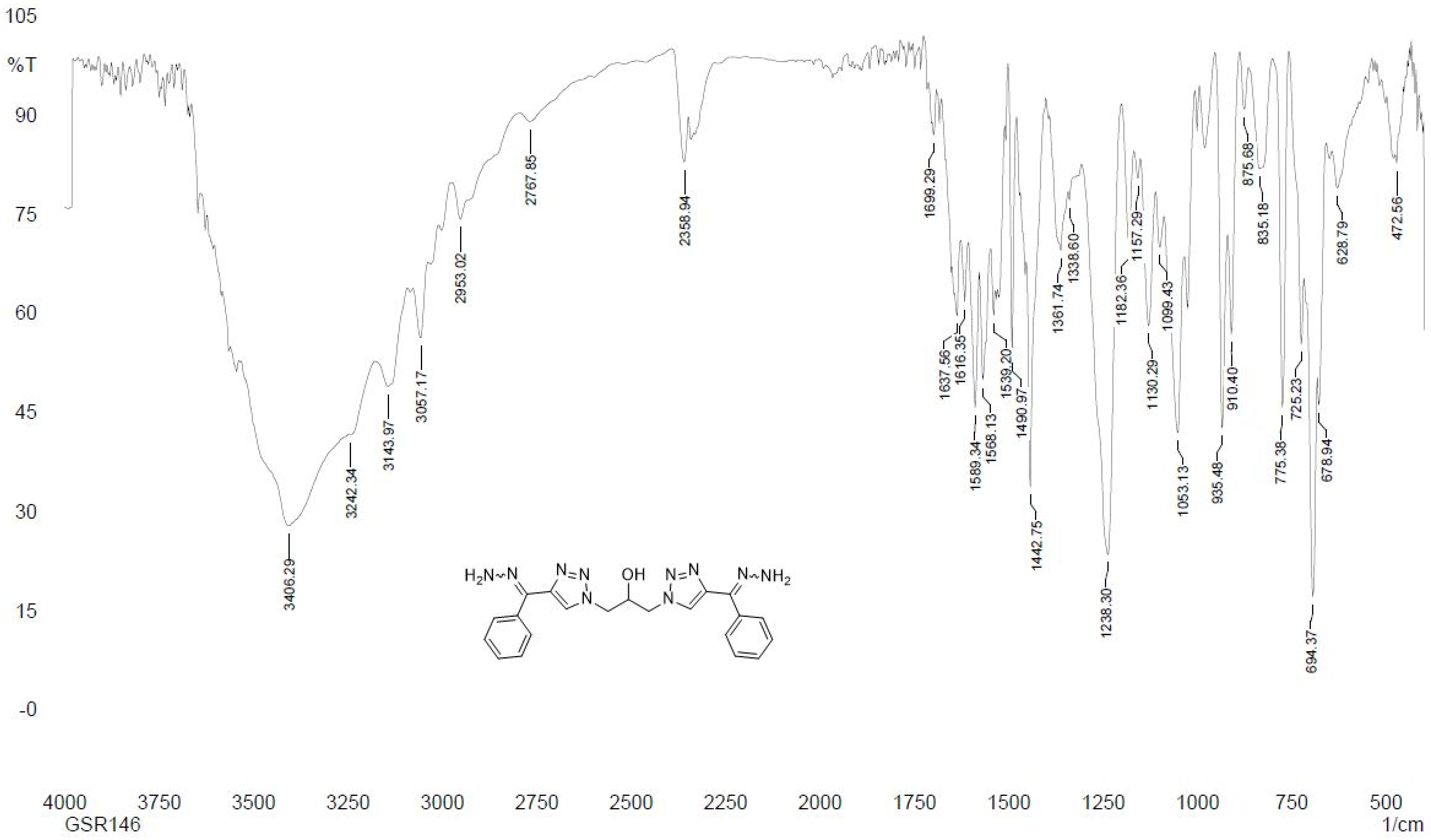

Figure 39S. FT-IR spectra (KBr disk) of 12a.

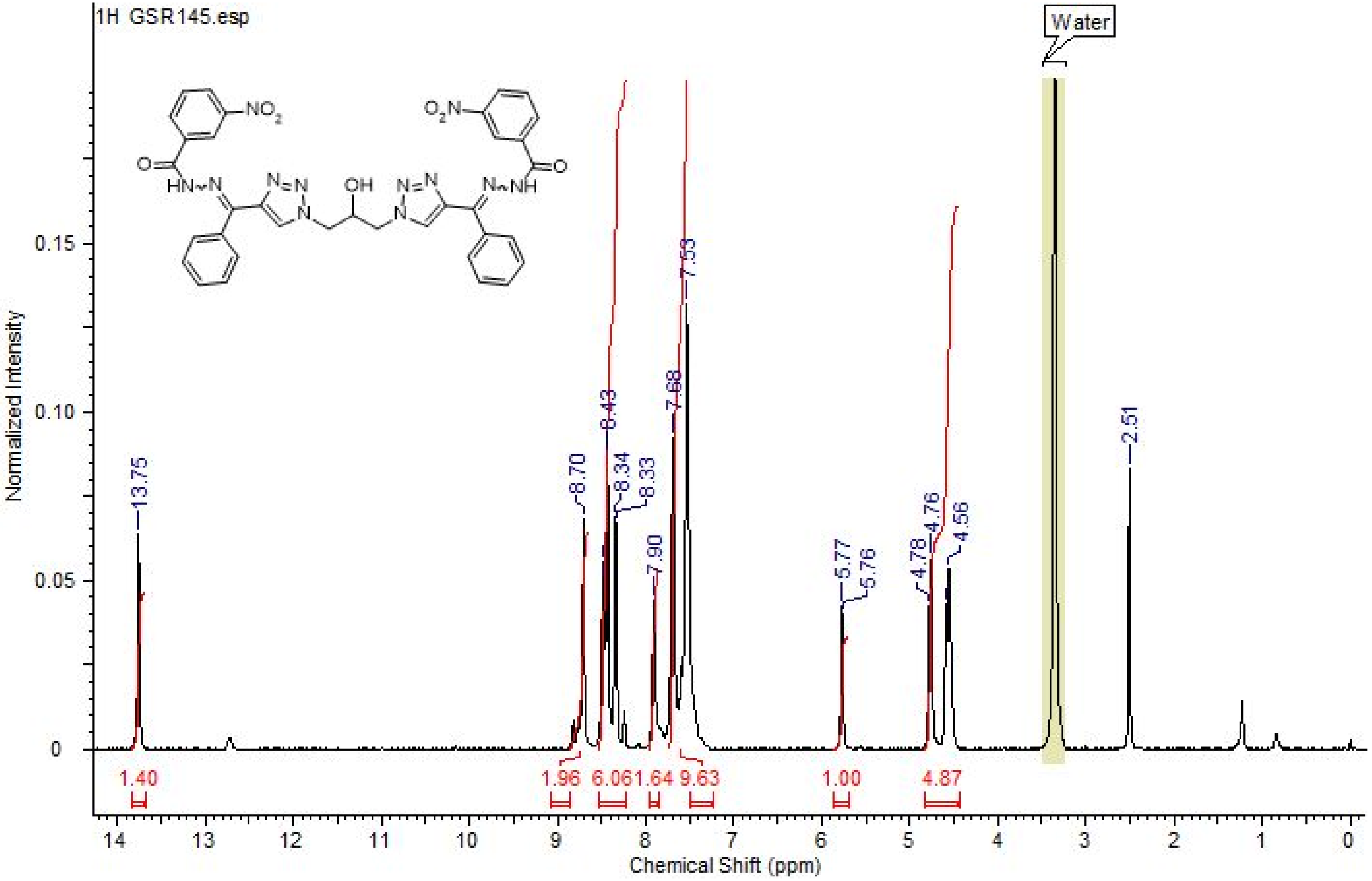

Figure 40S. ^{1}H NMR spectra (500 MHz, DMSO-d_6) of 12b.

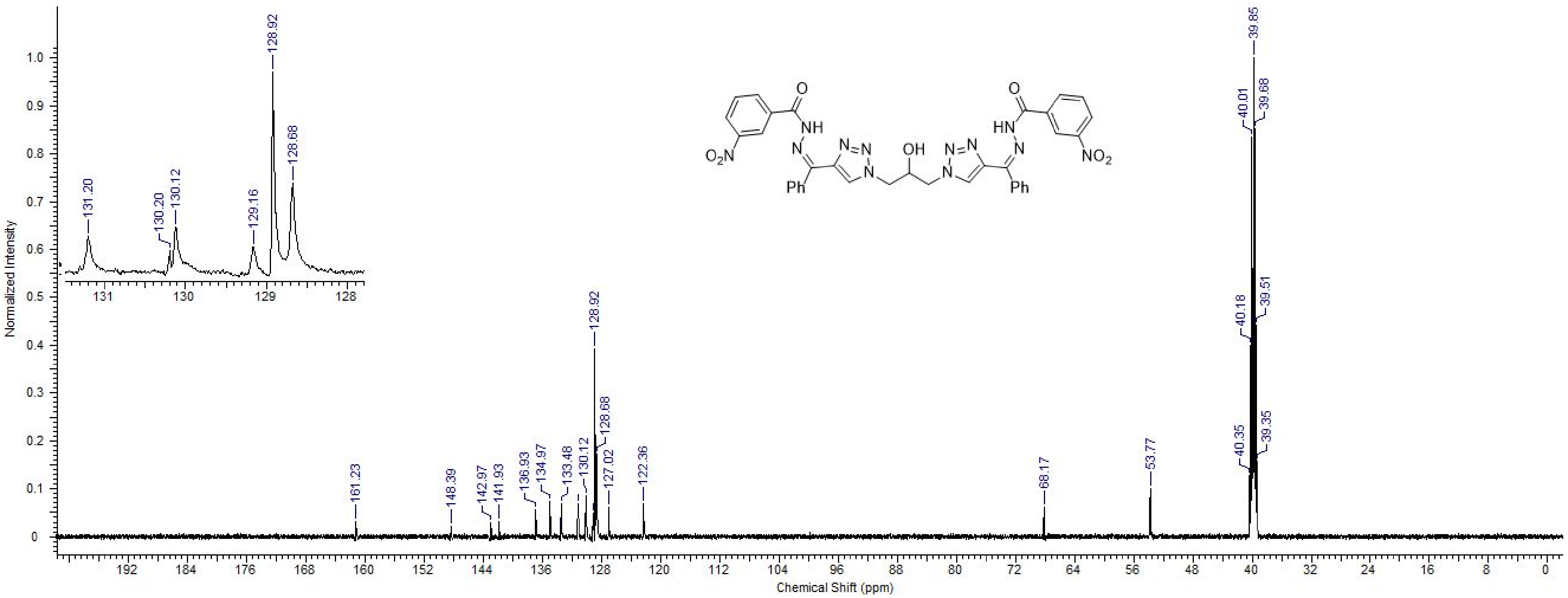

Figure 41S. ^{13}C NMR spectra (125 MHz, DMSO-d$_6$) of 12b**.**

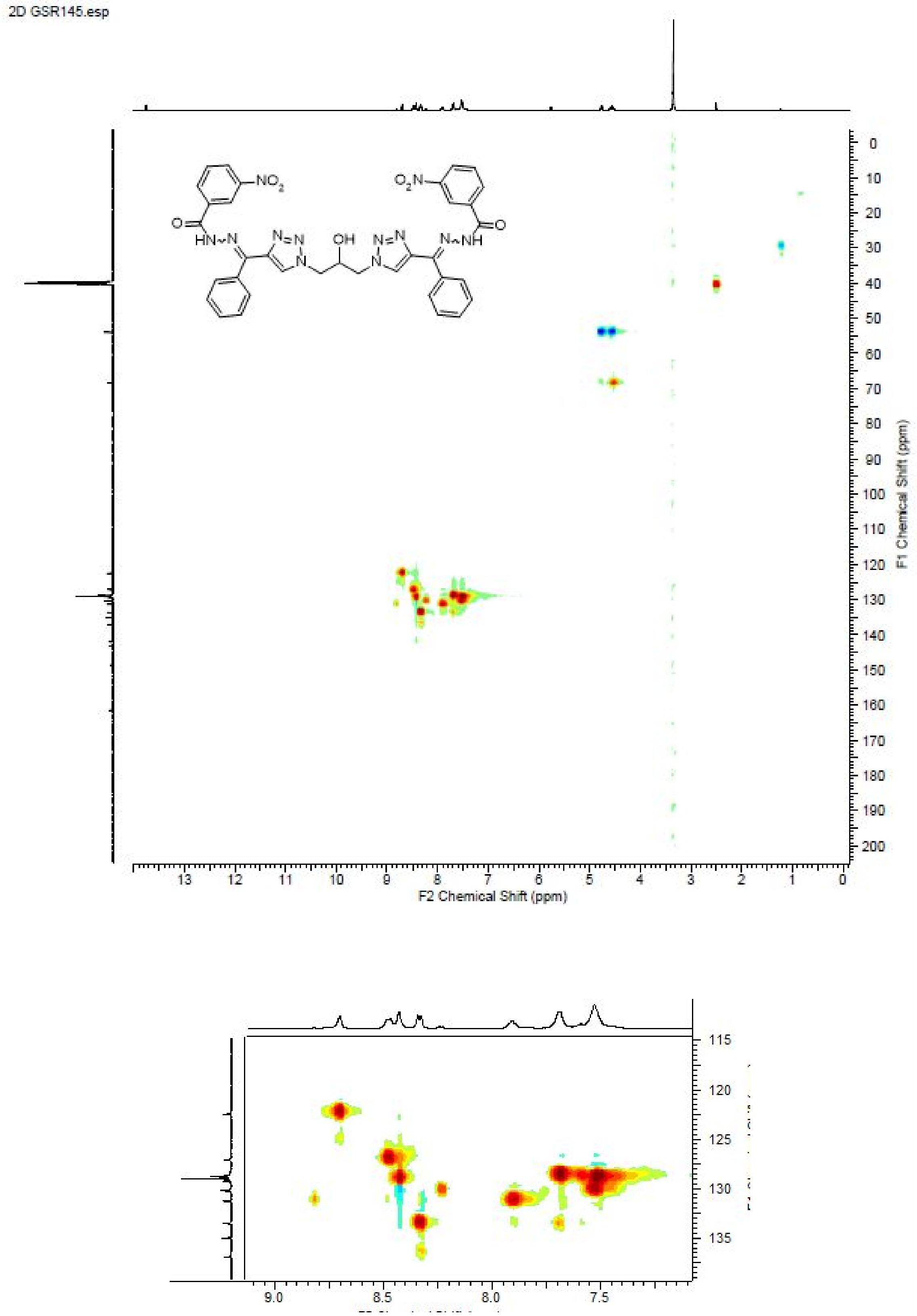

Figure 42S. HSQC spectra of 12b.

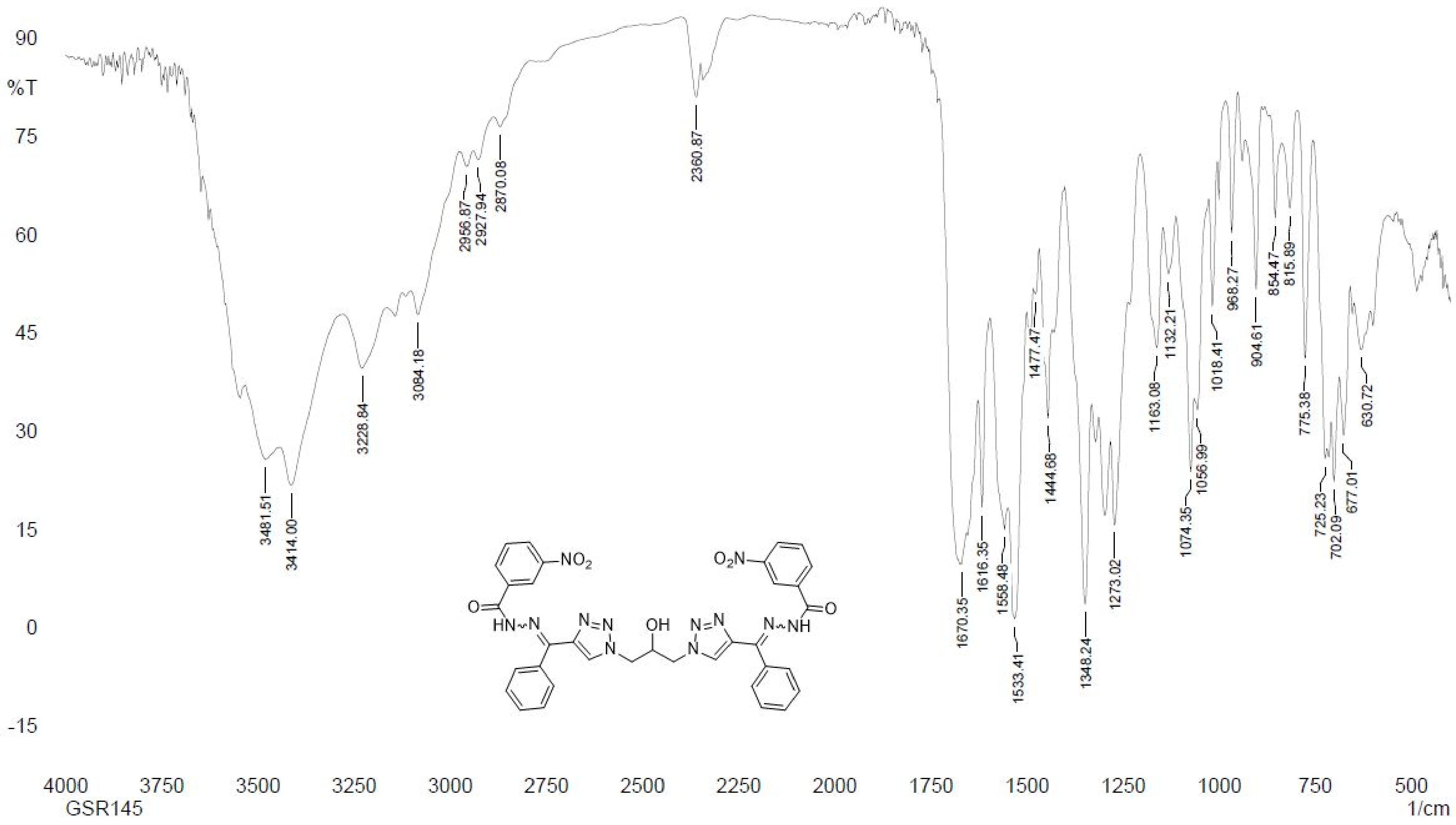

Figure 43S. FT-IR spectra (KBr disk) of 12b.

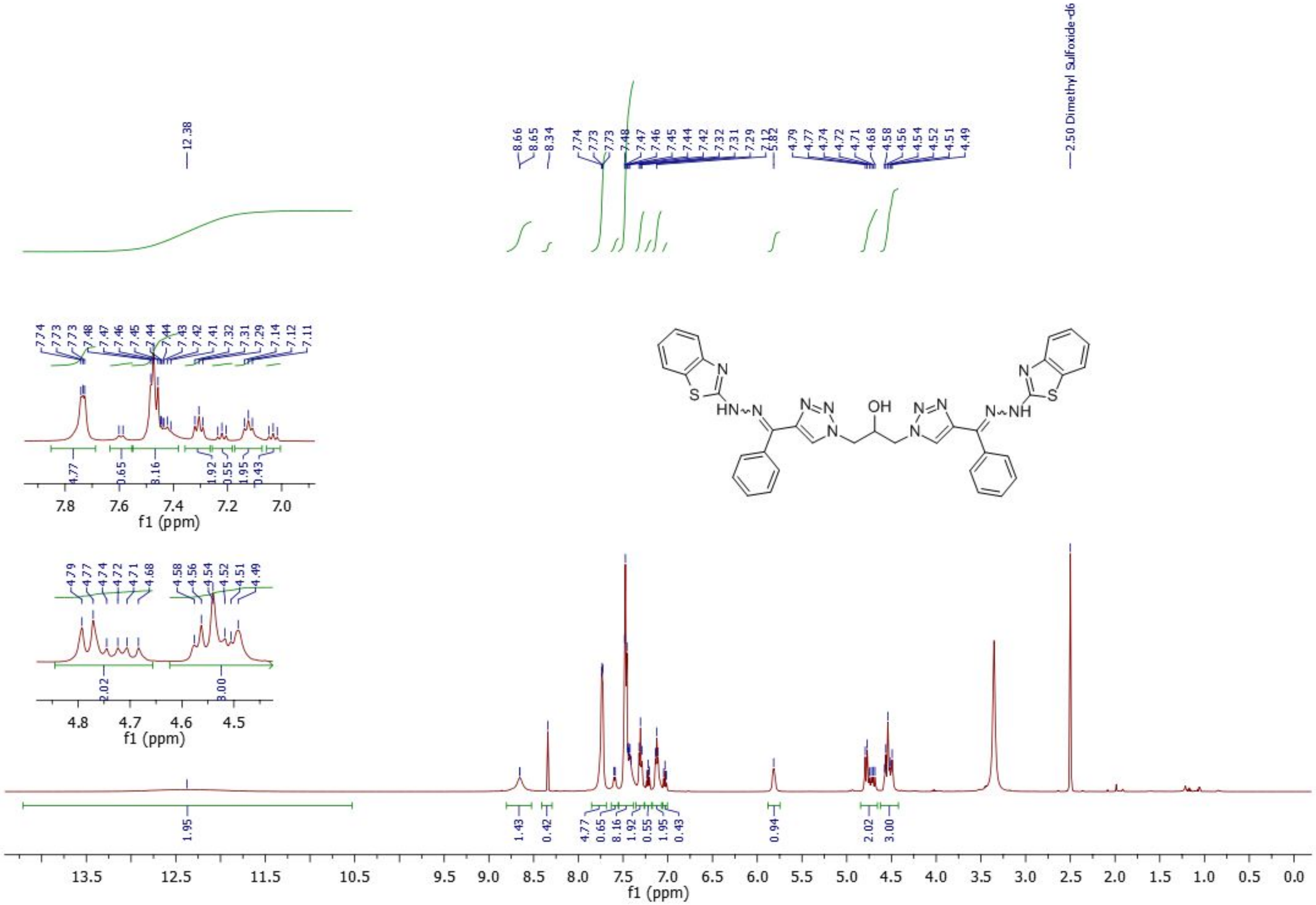

Figure 44S. ^{1}H NMR spectra (500 MHz, DMSO-d_6) of 12c.

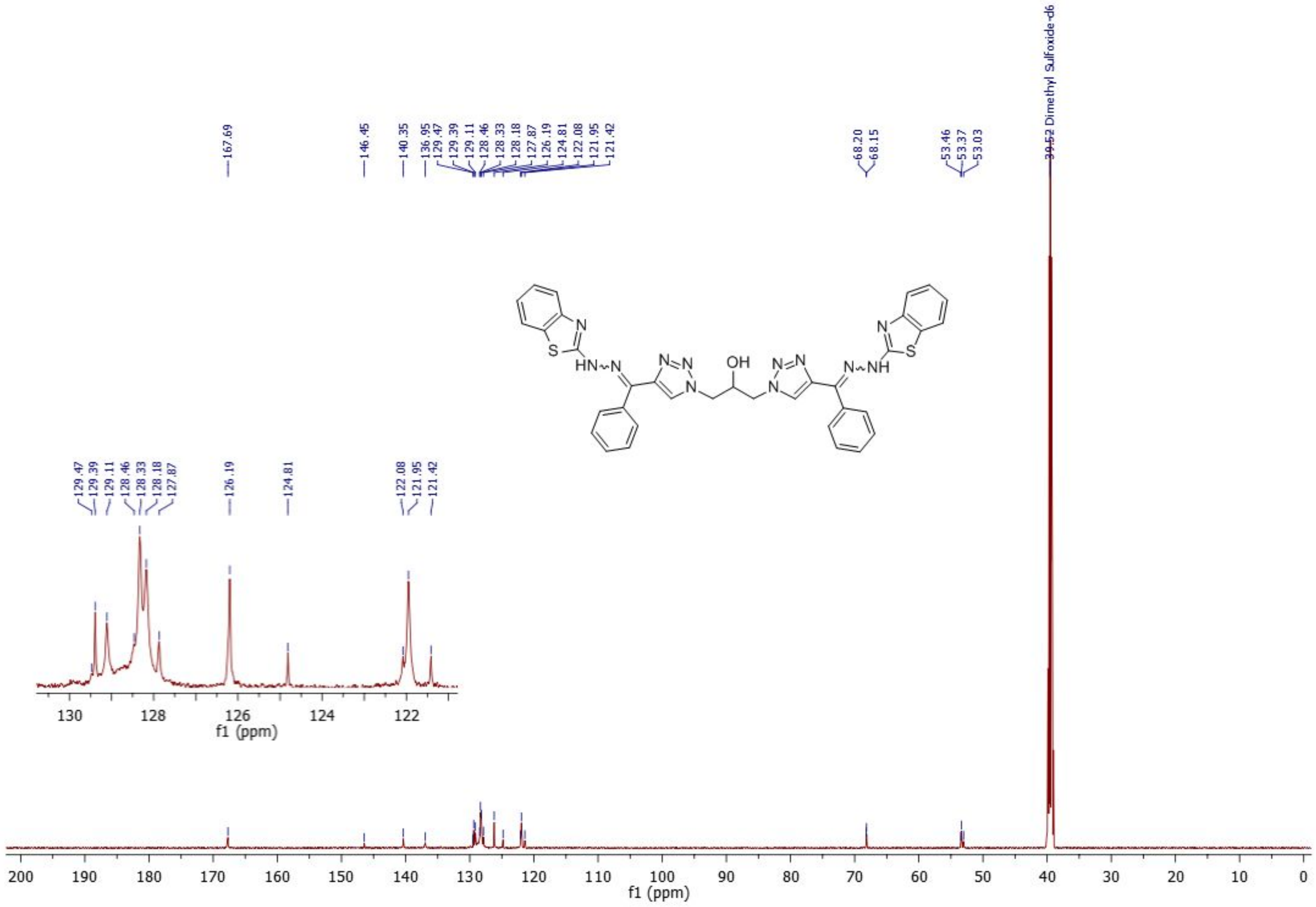

Figure 45S. ^{13}C NMR spectra (125 MHz, DMSO-d_6) of 12c**.**

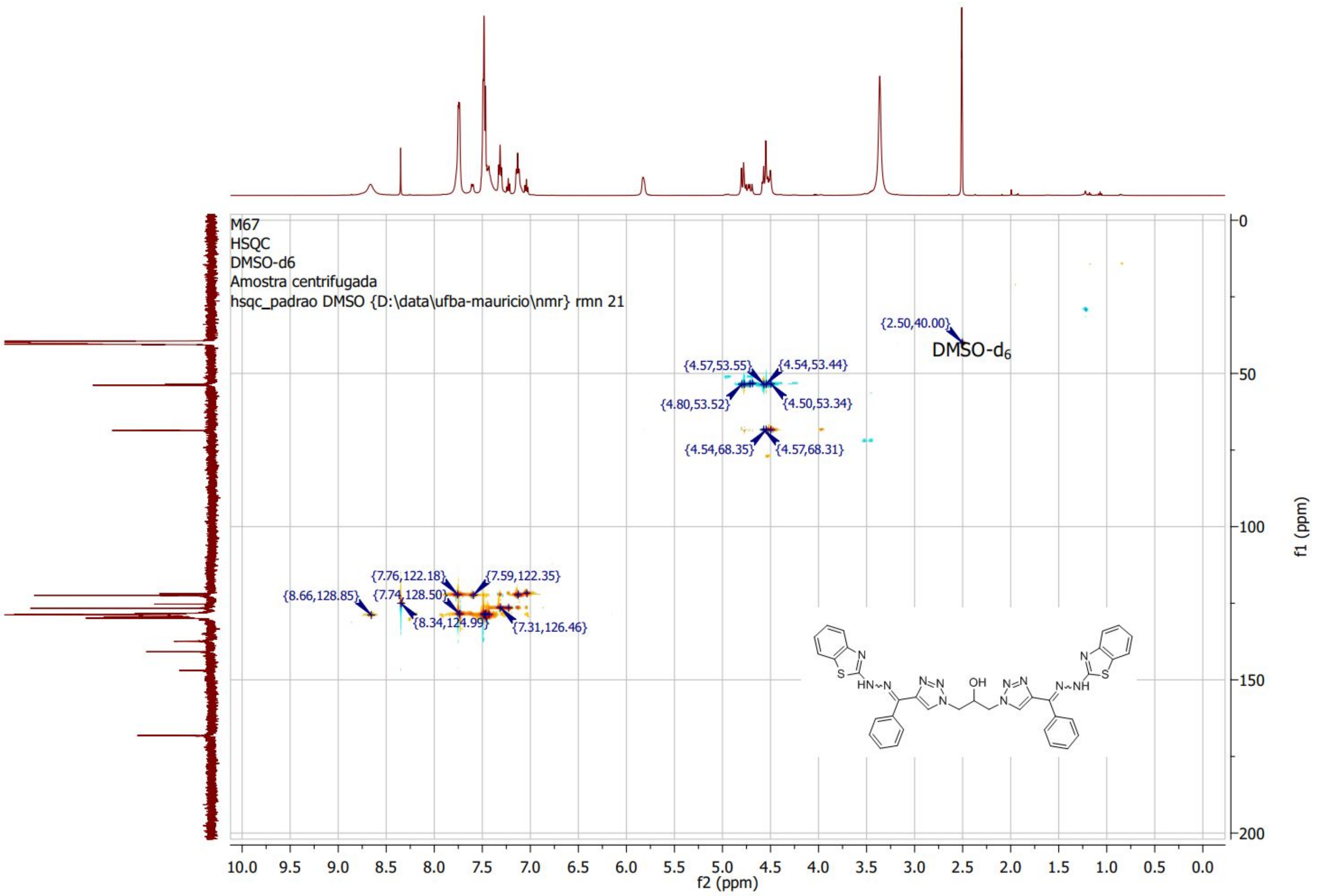

Figure 46S. HSQC spectra of 12c.

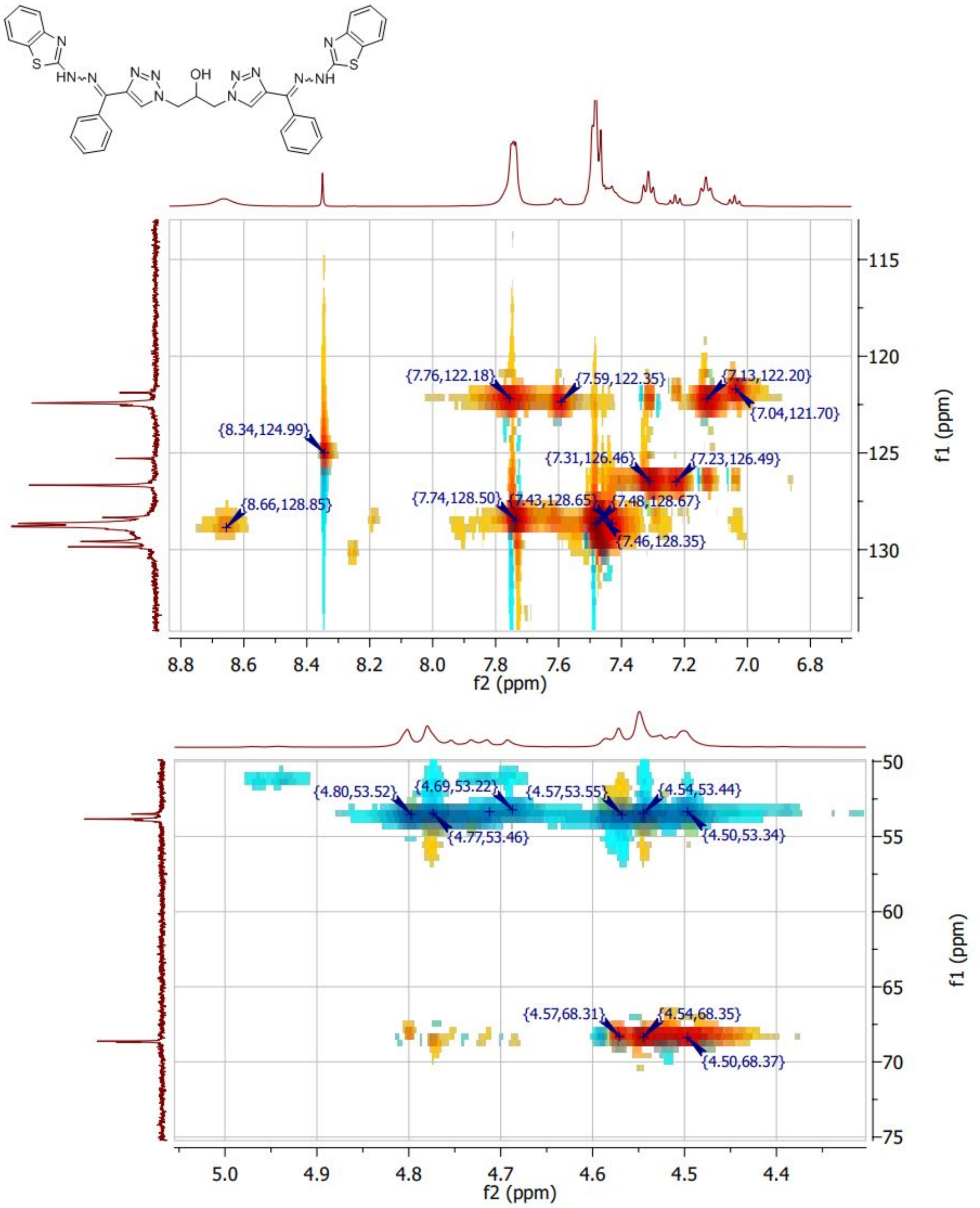

Figure 47S. Expanded HSQC spectra of 12c.

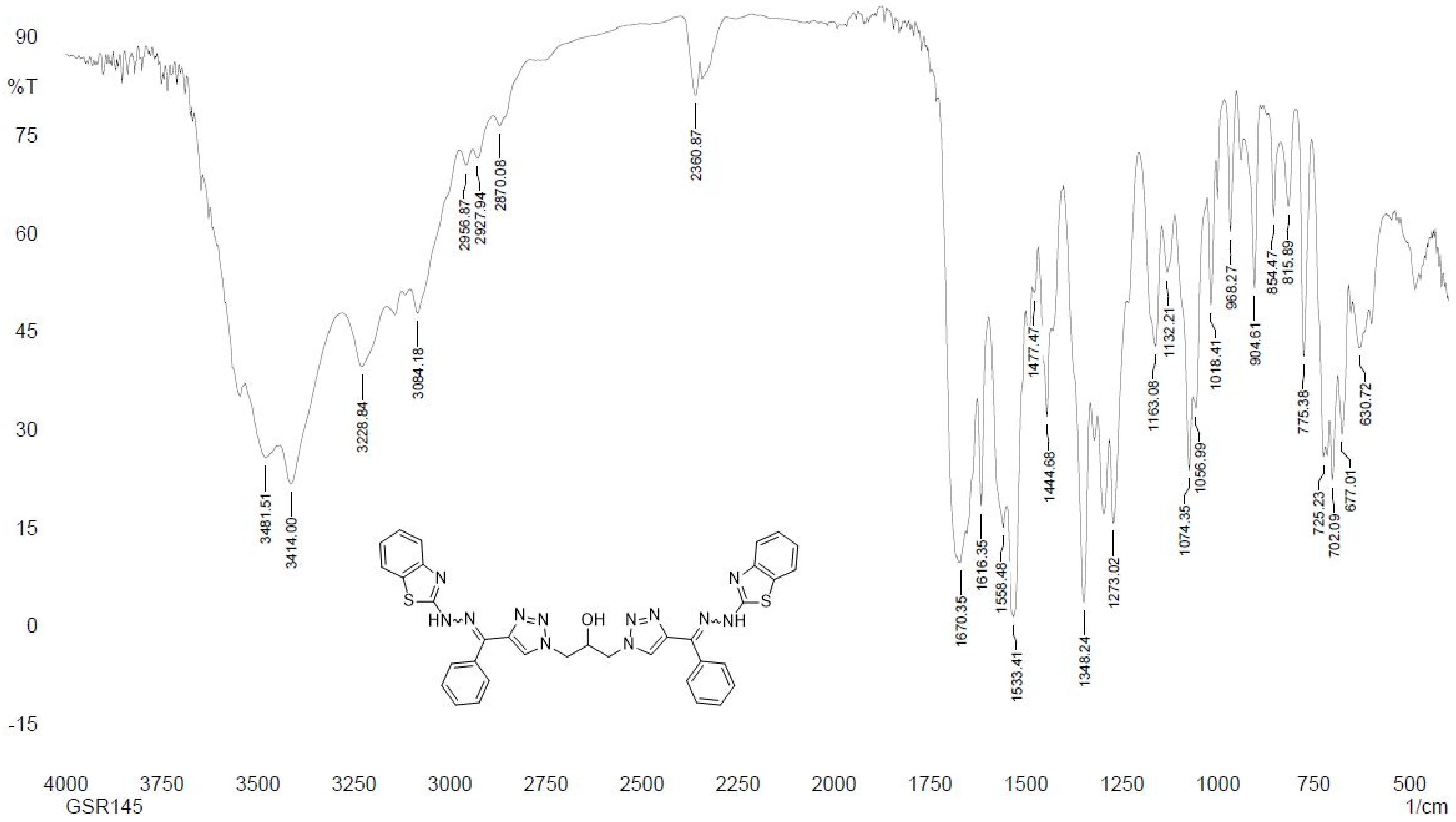

Figure 48S. FT-IR spectra (KBr disk) of 12c.
